# Supplementary material for: Total synthesis of cyrneines A–B and glaucopine C
Source: Nat Commun. 2018 Jun 1;9:2148. doi: 10.1038/s41467-018-04480-6 (PMC5984623; doi:10.1038/s41467-018-04480-6)
Supplement: Supplementary file 1 — Supplementary Information [file 41467_2018_4480_MOESM1_ESM.pdf]

Supplementary Information

for

Total Synthesis of Cyrneines A−B and Glaucopine C

Wu *et al.*

Table of Contents

Part I. Synthesis of Cyrneines A−B and Glaucopine C

1. **General Information……………………………………………………………S3**
2. **Experimental Procedures and Characterization Data………………………………..S4**
3. **Comparison of Spectroscopic Data of Natural and Synthetic Cyrneine A……….. S19**
4. **Copies of NMR Spectra…………………………………..…………………...S25**
5. **X-Ray Single Crystal Diffraction Data for Cyrneine A (1)………………………….S47**
6. **HPLC Charts for compound 12…………………………………………..…..S54**

Part II. Cross-coupling of Enol Triflates

7. **Experimental Procedures and Characterization Data……………………………...S55**
8. **Copies of NMR Spectra of Coupling Products………………………………………S69**
9. **Supplementary References…………………………………………………………...S91**

Part I. Synthesis of Cyrneines A, B, and Glaucopine C

1. General Information

Unless otherwise stated, all oxygen or moisture sensitive reactions were conducted in flame-dried glassware under an atmosphere of nitrogen or argon. All solvents were purified and dried according to standard methods prior to use. Reagents were purchased from commercial sources and were used without further purification.

Analytical thin layer chromatography (TLC) was performed on 0.2 mm thick silica gel 60-F254 plates (Merck) and visualized by exposure to ultraviolet light, or an ethanolic solution of phosphomolybdic acid. Chromatographic purification of products was accomplished using forced-flow chromatography on 230-400 mesh silica gel. The ^{1}H-NMR spectra were recorded on Bruker AV at 300, 400, or 600 MHz and ^{13}C-NMR spectra were recorded at 103 MHz, 126 MHz, or 151 MHz. Chemical shifts are given relative to TMS or the appropriate solvent peak. High resolution mass spectra (HRMS) were obtained on an IonSpec Ultima 7.0 T FT-ICR-MS (IonSpec, USA) using ESI as ionization method. HPLC analysis was performed on Shimadzu LC solution (CTO-10AS, LC-20AD, RID-10A). X-ray crystallographic analysis was performed on a Bruker D8 ADVANCE diffractometer with Cu-$K\alpha$ radiation (λ = 1.54178 Å).

2. Experimental Procedures and Spectroscopic Data of the Synthesized Compounds

2.1 Synthesis of compound 12

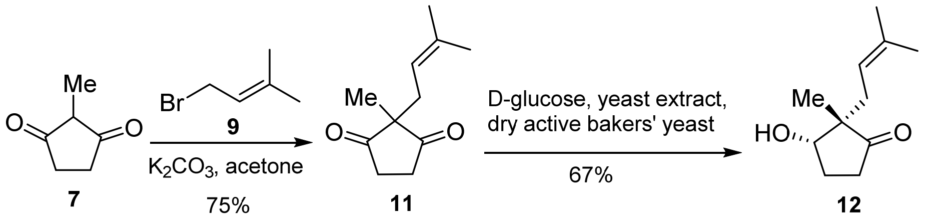

Supplementary Figure 1. Synthesis of compound **12**

To a solution of **7** (22.43 g, 200 mmol) in acetone (500 mL) was added **9** (46.54 mL, 400 mmol, 2.0 equiv), and K_2CO_3 (41.46 g, 300 mmol, 1.5 equiv). The mixture was stirred at room temperature overnight and then concentrated under reduced pressure. The crude residue was partitioned between water (100 mL) and CH_2Cl_2 (100 mL). The organic phase was separated, and the aqueous layer was extracted with CH_2Cl_2 (2 × 100 mL). The combined extracts were dried over Na_2SO_4 and concentrated under reduced pressure. The crude residue was purified by flash chromatography on silica gel (eluent: petroleum ether/EtOAc = 5/1) to afford **11** (27.02 g, 75%) as a light brown oil.

To a solution of D-glucose (90.0 g) and yeast extract (3.0 g) in distilled water (600 mL) at 35-40 °C was added dry active bakers' yeast (80.0 g, Angel Yeast Co., Ltd). The mixture was stirred open to the air for 30 min, maintaining the temperature between 30 and 35 °C, after which the dione **11** (6.0 g, 33.29 mmol) was added dropwise over 5 min. The mixture was vigorously stirred open to the air at 25 °C for 36 h, then diluted with 150 mL of water, and extracted with ethyl acetate (4 × 400 mL). The organic extract was evaporated to provide a crude product consisting of ketol, unreacted dione, and a trace of diol. The components of the mixture were purified by chromatography on silica gel (eluent: petroleum ether/EtOAc = 5/1 →2/1) to afford **11** (4.1 g, 67%) as a white solid. R_f = 0.3 (petroleum ether/EtOAc = 3/2); $[\alpha]_D^{23}$ = + 99.0 (*c* = 1.0 in $CHCl_3$); ^1H NMR (300 MHz, $CDCl_3$) δ 5.24–5.14 (m, 1H), 4.16–4.09 (m, 1H), 2.57–2.41 (m, 5H), 2.03–1.93 (m, 2H), 1.73 (s, 3H), 1.66 (s, 3H), 1.00 (s, 3H); ^{13}C NMR (126 MHz, $CDCl_3$) δ 221.4, 135.1, 119.3, 77.6, 53.8, 34.3, 29.5, 27.7, 26.2, 19.9, 18.0; HRMS (ESI) m/z calcd for $C_{11}H_{18}NaO_2^+$ $[M+Na]^+$: 205.1199, found 205.1198; HPLC chiralcel OZ-H column (hexane/isopropanol = 95/5, 1.0 mL/min), Retention time: 8.386 min (major), 15.956 min (minor): 98.9% ee.

2.2 Synthesis of compound 13

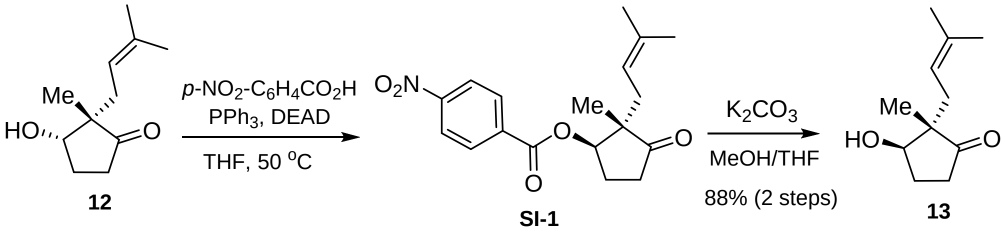

Supplementary Figure 2. Synthesis of compound **13**

To a stirred solution of **12** (3.28 g, 18.0 mmol) in THF (70 mL) containing triphenylphophine (9.44 g, 36.0 mmol, 2.0 equiv) and *p*-nitrobenzoic acid (6.02 g, 36.0 mmol, 2.0 equiv) was added DEAD (5.67 mL, 36.0 mmol, 2.0 equiv) dropwise over a 10-min period at 0 °C under argon, and the reaction mixture was stirred at 50 °C overnight. The solvent was removed under reduced pressure to leave an oil, which was purified by column chromatography (eluent: petroleum ether/EtOAc = 15/1→10/1) to give product **SI-1** (5.37 g, 90%) as a white solid. R_f = 0.2 (petroleum ether/EtOAc = 10/1); $[\alpha]_D^{28}$ = – 57.0 (*c* = 1.0 in $CHCl_3$); ^{1}H NMR (300 MHz, $CDCl_3$) δ 8.31 (d, *J* = 9.0 Hz, 1H), 8.18 (d, *J* = 9.0 Hz, 1H), 5.55–5.48 (m, 1H), 5.09 (t, *J* = 7.8 Hz, 1H), 2.60–2.03 (m, 6H), 1.70 (s, 3H), 1.60 (s, 3H), 1.14 (s, 3H); ^{13}C NMR (126 MHz, $CDCl_3$) δ 218.6, 164.1, 150.8, 136.3, 135.5, 130.8, 123.8, 117.8, 79.3, 53.3, 35.0, 34.0, 26.2, 25.6, 18.1, 16.3.

To a solution of 4-Nitrobenzoate **SI-1** (5.37 g, 16.20 mmol) in the mixed solvents of THF (60 mL) and MeOH (30 mL) was added K_2CO_3 (4.48 g, 32.4 mmol) and the reaction mixture was stirred at 0 °C for 30 min. The solvent was removed and the crude residue was partitioned between water (100 mL) and CH_2Cl_2 (100 mL). The organic layer was separated, and the aqueous layer was further extracted with CH_2Cl_2 (2 × 100 mL). The combined organic layers were dried with Na_2SO_4, filtered, and then concentrated in vacuo. The crude residue was purified by flash chromatography on silica gel (eluent: petroleum ether/EtOAc = 5/1→5/2) to afford **13** (2.89 g, 98% yield) as a white solid. R_f = 0.3 (petroleum ether/EtOAc = 2/1); $[\alpha]_D^{28}$ = + 88.0 (*c* = 1.0 in $CHCl_3$); ^{1}H NMR (300 MHz, $CDCl_3$) δ 5.15–5.05 (m, 1H), 4.23–4.15 (m, 1H), 2.52–2.40 (m, 1H), 2.32–2.09 (m, 4H), 1.92–1.80 (m,1H), 1.72 (s, 3H), 1.63 (s, 3H), 1.59 (s, 1H), 1.01 (s, 3H). ^{13}C NMR (101 MHz, $CDCl_3$) δ 220.8, 135.4, 118.8, 75.6, 53.8, 35.1, 33.8, 27.7, 26.1, 18.0, 15.2; HRMS (ESI) m/z calcd for $C_{11}H_{18}NaO_2^+$ $[M+Na]^+$: 205.1199, found 205.1198.

2.3 Synthesis of compound 15a

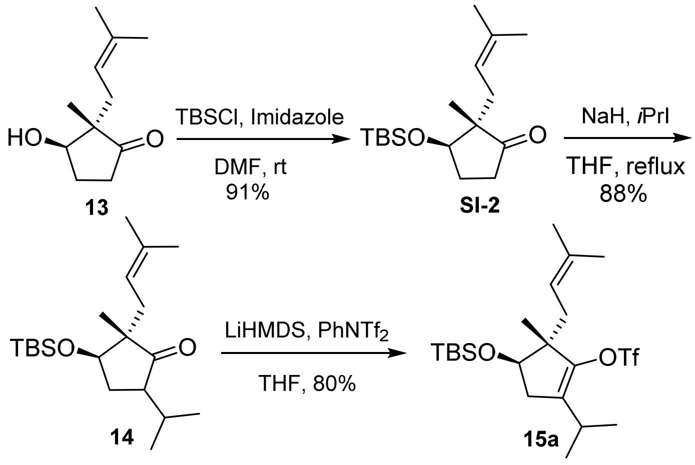

Supplementary Figure 3. Synthesis of compound **15a**

To a solution of compound **13** (5.70 g, 31.27 mmol) in DMF (60 ml) was added imidazole (2.56 g, 37.52 mmol, 1.2 equiv) and TBSCl (5.66 g, 37.52 mmol, 1.2 equiv). The mixture was stirred overnight at room temperature. The reaction mixture was poured into sodium bicarbonate solution (50 mL) and extracted with ethyl acetate (3 × 120 mL). The combined organic layers were washed with brine (5 × 30 mL), dried over Na_2SO_4 and evaporated to dryness. The resulting crude residue was purified by flash column chromatography (eluent: petroleum ether/EtOAc = 30/1) to afford **SI-2** (8.45 g, 91%) as a colorless oil. R_f = 0.4 (petroleum ether/EtOAc = 15/1); $[\alpha]_D^{[illegible]}$ = + 8.0 (*c* = 0.98 in $CHCl_3$); ^1H NMR (300 MHz, $CDCl_3$) δ 5.07–4.97 (m, 1H), 4.17–4.10 (m, 1H), 2.48–2.33 (m, 1H), 2.22–2.02 (m, 4H), 1.88–1.74 (m, 1H), 1.70 (s, 3H), 1.60 (s, 3H), 0.95 (s, 3H), 0.89 (s, 9H), 0.07 (s, 3H), 0.05 (s, 3H); ^{13}C NMR (126 MHz, $CDCl_3$) δ 221.2, 134.5, 119.2, 75.8, 54.4, 35.2, 33.7, 28.8, 26.1, 25.9, 18.2, 18.1, 16.1, -4.2, -4.9; HRMS (ESI) m/z calcd for $C_{17}H_{32}NaO_2Si^+$ $[M+Na]^+$: 319.2064, found 319.2064.

To a stirred suspension of NaH (60% dispersion in mineral oil, 5.7 g, 142.5 mmol, 5.0 equiv) in THF (160 mL) was added dropwise a solution of **SI-2** (8.45 g, 28.5 mmol) in THF (40 mL) at 0 °C, then 2-iodopropane (28.5 mL, 285.0 mmol, 10.0 equiv) was added. The reaction mixture was refluxed overnight. The resulting mixture was quenched at 0 °C by adding H_2O. Then 2 M HCl (80 mL) was added, and the mixture stirred at room temperature for 1.5 h. Then brine was added to the reaction mixture, and the layers were separated. The aqueous layer was extracted with ethyl acetate (3 × 100 mL), and the combined organic layers were washed with brine (50 mL), dried over Na_2SO_4 and concentrated under reduced pressure. The resulting crude residue was purified by flash column chromatography (eluent: petroleum ether/CH_2Cl_2 = 3/1) to afford **14** (8.46 g, 88% yield) as a colorless oil.

Ketone **14** (8.57 g, 25.31 mmol) was dissolved in dry THF (50 mL) and the mixture was cooled to -78 °C. LiHMDS (1.0 M in THF, 32.9 mL, 32.90 mmol, 1.3 equiv) was added and the clear solution was stirred at this temperature for 1 h. Then $PhNTf_2$ (11.75 g, 32.90 mmol, 1.3 equiv) dissolved in dry THF (50 mL) was slowly added and the mixture was allowed to warm to room temperature and stirred for 3 h. Then brine (50 mL) was added to the reaction mixture, and the layers were separated. The aqueous layer was extracted with ethyl acetate (3 × 50 mL), and the combined organic layers were dried over Na_2SO_4 and concentrated under reduced pressure. The residue was purified by flash chromatography (eluent: petroleum ether/CH_2Cl_2 = 50/1) to afford **15a** (9.52 g, 80% yield) as a colorless oil. R_f = 0.4 (petroleum ether/CH_2Cl_2 = 50/1); $[\alpha]_D^{25}$ = – 22.5 (c = 1.0 in $CHCl_3$); ^1H NMR (300 MHz, $CDCl_3$) δ = 5.09–4.99 (m, 1H), 4.04 (t, J = 6.6 Hz, 1H), 2.82 (sept, J = 6.9 Hz, 1H), 2.46 (dd, J = 15.3, 7.2 Hz, 1H), 2.20–1.95 (m, 3H), 1.70 (s, 3H), 1.60 (s, 3H), 1.03 (s, 3H), 1.01(d, J = 6.9 Hz, 3H), 1.01 (d, J = 6.9 Hz, 3H), 0.89 (s, 9H), 0.05 (s, 3H), 0.04 (s, 3H); ^{13}C NMR (101 MHz, $CDCl_3$) δ 143.9, 134.9, 134.0, 118.8 (d, J = 320 Hz), 119.8, 73.8, 50.6, 35.1, 34.6, 26.1, 25.9, 25.6, 20.6, 20.1, 18.1, 17.4, -4.1, -4.9; HRMS (ESI) m/z calcd for $C_{21}H_{38}F_3O_4SSi^+$ $[M+H]^+$: 471.2207, found 471.2197.

2.4 Synthesis of compound 17a

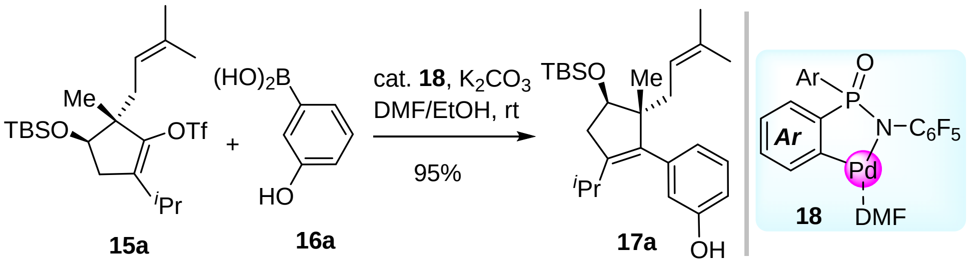

Supplementary Figure 4. Synthesis of compound **17a**

To a solution of vinyl triflate **15a** (6.60 g, 14.0 mmol) in the mixed solvents of DMF (60 mL) and EtOH (60 mL) was added arylboronic acid **16a** (3.86 g, 28.0 mmol, 2.0 equiv), palladacycle **18** (0.40 g, 5 mol %), K_2CO_3 (3.87 g, 28.0 mmol, 2.0 equiv) at room temperature under nitrogen. The resulting mixture was stirred at the same temperature until the vinyl triflate had disappeared as monitored by TLC analysis. The reaction mixture was then poured into water (60 mL) and extracted with ethyl acetate (3 × 60 mL). The organic layers were combined, washed with brine (5 × 15 mL), dried over Na_2SO_4 and concentrated under vacuum. The residue was purified by silica gel column chromatography (eluent: petroleum ether/EtOAc = 15/1) to afford the desired cross-coupling product **17a** (5.50 g, 95%). R_f = 0.4

(petroleum ether/EtOAc = 5/1); $[\alpha]_D^{23}$ = – 36.4 (c = 1.0 in $CHCl_3$); ^{1}H NMR (300 MHz, $CDCl_3$) δ 7.18 (t, J = 7.8 Hz, 1H), 6.73 (dd, J = 7.8, 1.5 Hz, 1H), 6.66 (d, J = 7.8 Hz, 1H), 6.57 (s, 1H), 5.10–5.00 (m,1H), 4.63 (s, 1H), 4.12 (t, J = 6.9 Hz, 1H), 2.51 (dd, J = 15.6, 7.2 Hz, 1H), 2.41 (sept, J = 6.9 Hz, 1H), 2.17 (dd, J = 15.6, 6.6 Hz, 1H), 2.05–1.81 (m, 2H), 1.66 (s, 3H), 1.49 (s, 3H), 1.01 (s, 3H), 0.97 (d, J = 6.9 Hz, 3H), 0.93 (s, 9H), 0.89 (d, J = 6.9 Hz, 3H), 0.08 (s, 3H), 0.06 (s, 3H); ^{13}C NMR (101 MHz, $CDCl_3$) δ 155.0, 141.9, 140.7, 140.2, 132.2, 128.9, 122.4, 121.7, 116.5, 113.3, 76.6, 54.3, 37.4, 36.4, 27.6, 26.2, 26.0, 21.7, 21.3, 19.4, 18.3, 18.1, -4.0, -4.9; HRMS (ESI) m/z calcd for $C_{26}H_{42}NaO_2Si^+$ $[M+Na]^+$: 437.2846, found 437.2840.

2.5 Synthesis of compound 23

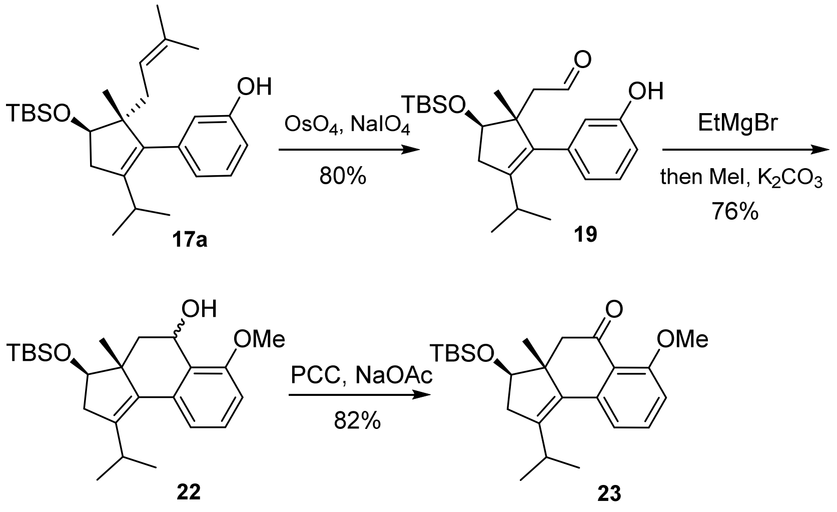

Supplementary Figure 5. Synthesis of compound **23**

To a solution of **17a** (6.43 g, 15.5 mmol) in the mixed solvents of dioxane (125 mL) and H_2O (25 mL) was added $NaIO_4$ (16.58 g, 77.5 mmol, 5.0 equiv), pyridine (3.74 mL, 46.5 mmol, 3.0 equiv) and 15.5 mL of OsO_4 solution (0.04 M in H_2O). The mixture was stirred at 80 °C until compound **17** had disappeared as monitored by TLC. H_2O (100 mL) and EtOAc (200 mL) were added to the mixture. The organic phase was separated, and the aqueous layer was extracted with EtOAc (3 × 60 mL). The combined organic layer were dried over Na_2SO_4 and concentrated under vacuum. The resulting crude residue was purified by flash column chromatography (eluent: petroleum ether/EtOAc = 10/1) to afford crude **19** (4.8 g, 80%) as a brown oil.

To a solution of **19** (4.8 g) in THF (120 mL) was added EtMgBr (1.0 M in THF, 13.6 mL, 13.6 mmol) slowly at -78 °C, and the mixture was then warmed up to 40 °C, and stirred at the same temperature overnight. Then, K_2CO_3 (3.41 g, 24.7 mmol), MeI (3.8 mL, 61.0 mmol) and

DMF (120 mL) was added. The mixture was stirred at 55 °C for additional 10 h. The reaction mixture was then poured into water (100 mL) and extracted with ethyl acetate (4 × 125 mL), washed with brine (5 × 30 mL). The organic layer was dried and concentrated under vacuum. The residue was purified by silica gel column chromatography (eluent: petroleum ether/CH_2Cl_2/Et_2O = 30/3/1) to afford **22** (3.25 g, 65%) as a brown oil.

Compound **22** (3.8 g) was dissolved in CH_2Cl_2 (40 mL) and added to a mixture of PCC (8.19 g, 38.0 mmol), NaOAc (3.12 g, 38.0 mmol) and celite (~5 g) in CH_2Cl_2 (60 mL). The mixture was reacted at room temperature for 6 h and filtered through celite and washed repeatedly with Et_2O. The solvent was removed under reduced pressure to leave an oil, which was purified by column chromatography (eluent: petroleum ether/EtOAc = 20/1→10/1) to give product **23** (3.11 g, 82% yield) as a brown oil. R_f = 0.2 (petroleum ether/EtOAc = 6/1); $[\alpha]_D^{[illegible]}$ = + 185.9 (*c* = 1.0 in $CHCl_3$); ^1H NMR (300 MHz, $CDCl_3$) δ 7.45 (t, *J* = 8.1 Hz, 1H), 7.11 (d, *J* = 7.8 Hz, 1H), 6.89 (d, *J* = 8.4 Hz, 1H), 4.08 (t, *J* = 8.1 Hz, 1H), 3.92 (s, 3H), 3.23 (sept, *J* = 6.9 Hz, 1H), 2.73 (d, *J* = 15.9 Hz, 1H), 2.66 (dd, *J* = 15.6, 7.8 Hz, 1H), 2.55 (d, *J* = 15.9 Hz, 1H), 2.35 (dd, *J* = 15.6, 8.7 Hz, 1H), 1.18 (d, *J* = 6.9 Hz, 3H), 1.04 (d, *J* = 6.9 Hz, 3H), 0.97 (s, 3H), 0.91 (s, 9H), 0.09 (s, 3H), 0.09 (s, 3H); ^{13}C NMR (101 MHz, $CDCl_3$) δ 197.8, 160.3, 143.2, 139.6, 133.8, 132.8, 120.8, 120.2, 110.8, 80.3, 56.2, 55.4, 51.2, 38.3, 27.4, 26.0, 21.6, 20.9, 18.2, 17.9, -4.3, -4.6; HRMS (ESI) m/z calcd for $C_{24}H_{37}O_3Si^+$ $[M+H]^+$: 401.2506, found 401.2511.

2.6 Synthesis of compound 24

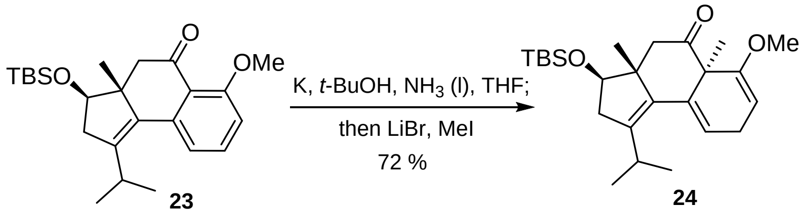

Supplementary Figure 6. Synthesis of compound **24**

A stirred solution of **23** (1.60 g, 4 mmol) and *t*-butyl alcohol (0.38 mL, 4 mmol, 1.0 equiv) in dry Et_2O (25 mL) and dry ammonia (25 mL) under N_2 at -78 °C was treated with potassium metal (0.39 g, 10 mmol, 2.5 equiv). After being stirred for 10 min, a solution of dry LiBr (0.86 g, 10 mmol) in dry THF (10 mL) was added dropwise. By stirring for an additional 20 min, methyl iodide (1.25 mL, 20 mmol, 5.0 equiv) was added. The reaction mixture was stirred at -78 °C for 1 h, and then warmed to room temperature over a period of 1 h while the ammonia was evaporated. Water (50 mL) was added to the residue, and the organic phase was

separated. The aqueous layer was extracted with EtOAc (2 × 50 mL). The combined organic layer were dried over Na_2SO_4 and concentrated under vacuum. The residue was purified by column chromatography (eluent: petroleum ether/CH_2Cl_2/Et_2O = 50/4/1) to afford compound **24** (1.2 g, 72%) as a light yellow solid. R_f = 0.5 (petroleum ether/CH_2Cl_2/Et_2O = 9/3/1); $[\alpha]_D^{28}$ = – 2.5 (*c* = 0.4 in $CHCl_3$); ^1H NMR (300 MHz, $CDCl_3$) δ 5.54–5.47 (m, 1H), 4.82–4.77 (m, 1H), 4.12 (t, *J* = 8.4 Hz, 1H), 3.6 (s, 3H), 2.97–2.82 (m, 3H), 2.67 (d, *J* = 12.3 Hz, 1H), 2.49 (dd, *J* = 15.0, 7.5 Hz, 1H), 2.29 (d, *J* = 12.3 Hz, 1H), 2.20 (dd, *J* = 15.0, 8.4 Hz, 1H), 1.48 (s, 3H), 0.99 (d, *J* = 6.9 Hz, 3H), 0.99 (d, *J* = 6.9 Hz, 3H), 0.89 (s, 9H), 0.85 (s, 3H), 0.06 (s, 3H), 0.05 (s, 3H). ^{13}C NMR (101 MHz, $CDCl_3$) δ 208.9, 154.6, 140.3, 136.0, 133.9, 123.3, 92.2, 82.3, 54.6, 54.6, 52.6, 50.9, 37.1, 26.6, 26.4, 25.9, 24.5, 21.5, 21.4, 18.2, 17.6, -4.4, -4.7; HRMS (ESI) m/z calcd for $C_{25}H_{41}O_3Si^+$ $[M+H]^+$: 417.2819, found 417.2816.

2.7 Synthesis of compound 27

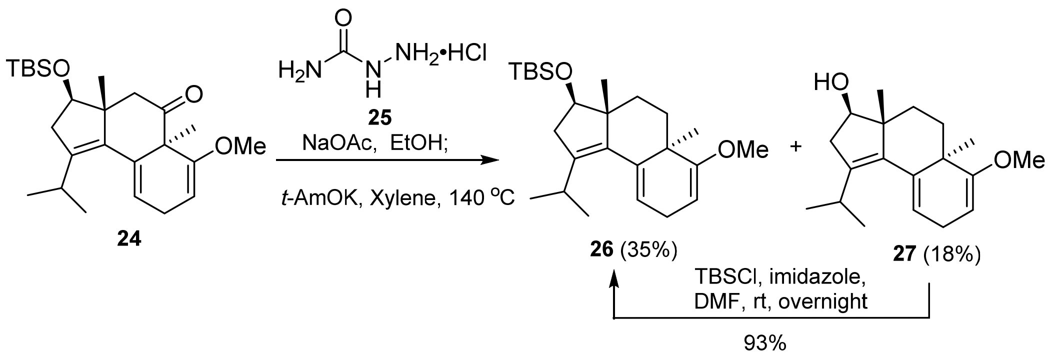

Supplementary Figure 7. Synthesis of compound **26**

To a solution of **24** (0.15 g, 0.36 mmol) in EtOH (8.0 mL) was added semicarbazide hydrochloride **25** (0.20 g, 1.8 mmol, 5.0 equiv) and NaOAc (0.15 g, 1.8 mmol, 5.0 equiv) under argon atmosphere. The mixture was stirred at 35 °C for 3 h. Reaction mixture was concentrated to dryness under reduced pressure and the residue was dissolved in ethyl acetate. Water (15 mL) and EtOAc (30 mL) were added to the residue, and the organic phase was separated. The aqueous layer was extracted with EtOAc (2 × 20 mL). The combined organic layers were washed with saturated $NaHCO_3$ solution (10 mL), water (10 mL) and brine (10 mL), then dried over Na_2SO_4 and evaporated under reduced pressure. A mixture of semicarbazone of **24**, degassed xylene (5.0 mL) and *t*-AmOK (0.23 g, 1.8 mmol) was heated at 140 °C for 1.8 h under argon atmosphere. Then water was added, and the xylene solution was separated. The aqueous layer was extracted with EtOAc (3 × 20 mL). The combined organic layers were dried over Na_2SO_4. The solvent was removed under reduced pressure. Purification of the residue by column chromatography (eluent: petroleum ether/CH_2Cl_2 = 25/1)

afforded **26** (51 mg, 35%) as a colorless oil and **27** (19 mg, 18%) as a white solid.

26: R_f = 0.3 (petroleum ether/CH_2Cl_2 = 10/1); ^1H NMR (300 MHz, $CDCl_3$) δ 5.36–5.31 (m, 1H), 4.56–4.50 (m, 1H), 3.92 (t, *J* = 8.4 Hz, 1H), 3.51 (s, 3H), 2.92–2.80 (m, 3H), 2.38 (dd, *J* = 15.0, 7.8 Hz, 1H), 2.13 (dd, *J* = 14.7, 9.0 Hz, 1H), 1.93–1.83 (m, 1H), 1.74–1.58 (m, 2H), 1.53–1.40 (m, 1H), 1.10 (s, 3H), 0.96 (d, *J* = 6.9 Hz, 3H), 0.92 (d, *J* = 6.9 Hz, 3H), 0.90 (s, 9H), 0.84 (s, 3H), 0.05 (s, 3H), 0.05 (s, 3H); ^{13}C NMR (151 MHz, $CDCl_3$) δ 160.7, 138.1, 137.6, 137.3, 119.3, 88.5, 82.8, 54.3, 49.6, 39.1, 37.0, 35.5, 33.1, 26.4, 26.2, 26.0, 23.4, 21.6, 21.5, 18.3, 16.5, -4.3, -4.8.

27: R_f = 0.3 (petroleum ether/EtOAc = 6/1);^1H NMR (300 MHz, $CDCl_3$) δ 5.39–5.33 (m, 1H), 4.56–4.51 (m, 1H), 4.02 (t, *J* = 8.7 Hz, 1H), 3.52 (s, 3H), 2.93–2.82 (m, 3H), 2.59 (dd, *J* = 15.0, 7.8 Hz, 1H), 2.14 (dd, *J* = 15.0, 9.3 Hz, 1H), 1.96–1.88 (m, 1H), 1.75–1.63 9m, 2H), 1.62–1.47 (m, 1H), 1.11 (s, 3H), 0.97 (d, *J* = 6.9 Hz, 3H), 0.93 (d, *J* = 6.9 Hz, 3H), 0.89 (s, 3H). **27** was converted to **26** by executing the same procedure for synthesis of **SI-2** in 93% yield.

2.8 Synthesis of compound 29

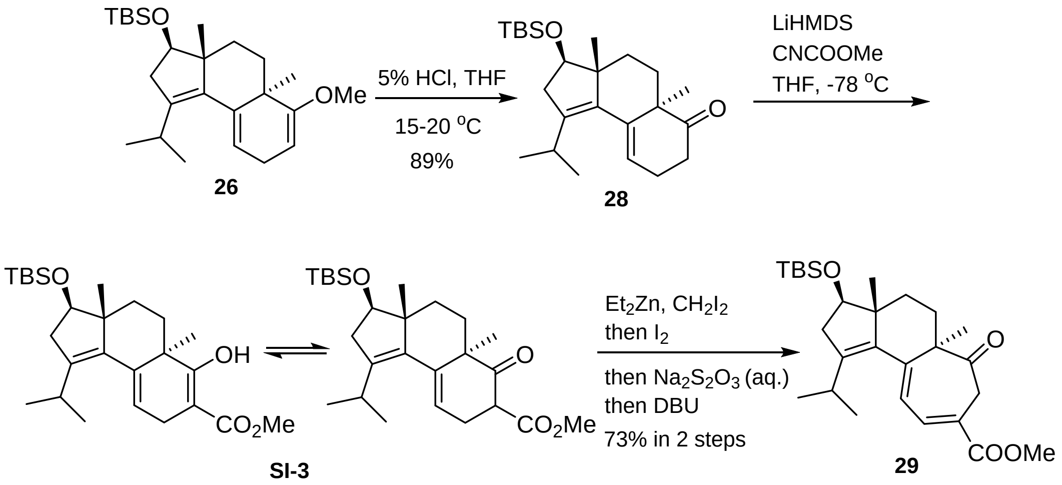

Supplementary Figure 8. Synthesis of compound **29**

To a solution of **26** (200 mg, 0.5 mmol) in degassed THF (6.0 mL) was added 5% HCl (1.5 mL) and the mixture was stirred at 15–20 °C for 40 min. Brine (20 mL) and ethyl acetate (20 mL) was added to the reaction mixture and the layers were separated. The aqueous layer was extracted with EtOAc (2 × 20 mL). The combined organic layers were washed with brine (20 mL), dried over Na_2SO_4, and concentrated in vacuo. The residue was chromatographed on silica gel (eluent: petroleum ether/Et_2O = 20/1) to give **28** (172 mg, 89%) as a white solid . R_f = 0.3 (petroleum ether/Et_2O = 10/1); $[\alpha]_D^{27}$ = + 229.0 (*c* = 0.8 in $CHCl_3$); ^1H NMR (300 MHz,

$CDCl_3$) δ 5.55–5.49 (m, 1H), 3.93 (t, *J* = 8.1 Hz, 1H), 2.88 (sept, *J* = 6.9 Hz, 1H), 2.74–2.65 (m, 1H), 2.53–2.36 (m, 4H), 2.15 (dd, *J* = 15.0, 9.0 Hz, 1H), 1.77–1.64 (m, 3H), 1.55–1.42 (m, 1H), 1.13 (s, 3H), 0.98 (d, *J* = 6.9 Hz, 3H), 0.95 (d, *J* = 6.9 Hz, 3H), 0.90 (s, 9H), 0.82 (s, 3H), 0.06 (s, 3H), 0.06 (s, 3H); ^{13}C NMR (151 MHz, $CDCl_3$) δ 215.6, 139.4, 139.1, 136.6, 121.7, 82.4, 49.7, 48.3, 37.1, 35.8, 34.7, 31.2, 26.3, 26.0, 25.6, 22.8, 21.6, 21.5, 18.2, 16.5, -4.3, -4.6; HRMS (ESI) m/z calcd for $C_{24}H_{40}NaO_2Si^+$ $[M+Na]^+$: 411.2690, found 411.2682.

To a solution of compound **28** (200 mg, 0.515 mmol) in dry THF (4 mL) at -78 °C was added LiHMDS (1M in THF, 2.06 mL, 2.06 mmol, 4 equiv) under N_2 atmosphere and the resulting solution was stirred for 15 min at -78 °C and then 20 min at 0 °C. After that methyl carbonocyanidate (0.164 mL, 2.06 mmol, 4 equiv) was added at -78 °C and the mixture was stirred for 1 h at the same temperature. The reaction mixture was quenched by addition of water. The organic phase was collected, and the aqueous layer was extracted with EtOAc. The combined organic layers were dried over Na_2SO_4 and concentrated under reduced pressure. Purification of the residue by flash chromatography yielded crude β-keto ester **SI-3**.

A 50-mL flask was equipped with a magnetic stirrer bar and charged with 3 mL CH_2Cl_2 and diethyl zinc (1.0 M in PhMe, 3.5 mL, 3.5 mmol) under an atmosphere of N_2 at 0 °C. Methylene iodide (0.282 mL, 3.5 mmol, 7 equiv.) was added. After the mixture was stirred for 5 min, β-keto ester SI-1 was added as a solution in 4 mL of CH_2Cl_2. The mixture was stirred for 2 h at room temperature. Then iodine (1.02 g, 4 mmol) was added to the reaction mixture in a single portion and the solution was allowed to stir until a pink color persisted for 10 min. A saturated solution of sodium thiosulfate was added and the mixture was stirred until the pink color had disappeared. To this solution was added DBU (1.49 mL, 10 mmol). The mixture was stirred for 20 min, washed with saturated aqueous ammonium chloride, and extracted three times with diethyl ether. The combined organic layers were dried with Na_2SO_4 and concentrated in vacuo. The residue was chromatographed on silica (eluent: petroleum ether/CH_2Cl_2/Et_2O = 100/10/1) to yield **29** (175 mg, 73% for 2 steps). R_f = 0.4 (petroleum ether/CH_2Cl_2/Et_2O = 9/3/1); $[\alpha]_D^{[illegible]}$ = + 20.0 (*c* = 0.14 in $CHCl_3$); ^1H NMR (300 MHz, $CDCl_3$) δ 7.29 (d, *J* = 6.0 Hz, 1H), 6.01 (d, *J* = 6.0 Hz, 1H), 3.98 (t, *J* = 8.4 Hz, 1H), 3.86 (d, *J* = 11.4 Hz, 1H), 3.80 (s, 3H), 3.36 (d, *J* = 11.4 Hz, 1H), 2.92 (sept, *J* = 6.9 Hz, 1H), 2.48 (dd, *J* = 15.0, 7.8 Hz, 1H), 2.22 (dd, *J* = 15.0, 8.7 Hz, 1H), 2.08–1.98(m, 1H), 1.63–1.52 (m,2H), 1.43 (dt, *J* =13.5, 4.8 Hz, 1H), 1.42 (s, 3H), 1.03 (d, *J* = 6.9 Hz, 3H), 0.98 (d, *J* = 6.9 Hz, 3H), 0.90 (s, 3H), 0.90 (s, 9H), 0.07 (s, 3H), 0.06 (s, 3H); ^{13}C NMR (101 MHz, $CDCl_3$) δ 206.8, 166.2, 148.3, 141.6, 138.8, 136.1, 124.0, 122.7, 81.1, 56.7, 52.4, 50.6, 41.5, 37.3, 34.5, 33.9, 26.6, 26.0, 23.4, 21.7, 21.3, 18.2, 17.5, -4.3, -4.7; HRMS (ESI) m/z calcd for $C_{27}H_{42}NaO_4Si^+$

[M+Na]$^+$: 481.2745, found 481.2740.

2.9 Synthesis of compound Cyrneine (1)

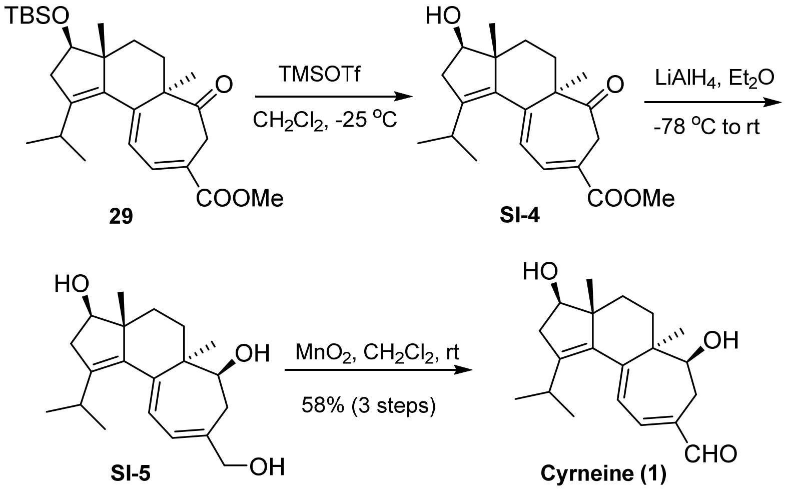

Supplementary Figure 9. Synthesis of cyrneine (**1**)

To a solution of **29** (20 mg, 0.043 mmol) in dry CH_2Cl_2 (2.5 ml) was added TMSOTf (39 µL, 0.215 mol, 5.00 equiv) at -25 °C. The clear solution was stirred at this temperature for 15 min and quenched by the addition of 1 mL of satd. aq. $NaHCO_3$. The aqueous layer was extracted with CH_2Cl_2. The combined organic layers were dried over Na_2SO_4, filtered and concentrated. Flash chromatography on silica gel (eluent: petroleum ether/EtOAc = 3/1) gave compound **SI-4** (12 mg, 81%).

To a solution of **SI-4** (12 mg) in Et_2O (3 mL) was added LAH (8 mg, 0.21 mmol, 6.0 equiv) at -78 °C. The reaction was stirred for 10 min at -78 °C under nitrogen and the solution was warmed to room temperature. The solution was quenched by the addition of H_2O (2 mL). Brine (10 mL) and Et_2O (20 mL) was added to the reaction mixture and the layers were separated. The aqueous layer was extracted with Et_2O (2 × 10 mL). The combined organic layers were washed with brine (10 mL), dried over Na_2SO_4, and concentrated in vacuo. The solvent was removed in vacuo and the residue was purified by flash chromatography (eluent: petroleum ether/EtOAc = 1/1) to give crude compound **SI-5**.

To a solution of **SI-5** in CH_2Cl_2 (3 mL) was added MnO_2 (26 mg, 0.3 mmol), and the resultant mixture was stirred at room temperature overnight. The solution was filtered, dried over Na_2SO_4, and concentrated under reduced. Flash chromatography of the residue on silica gel (eluent: petroleum ether/EtOAc = 10/1) gave pure Cyrneine A (8 mg, 58% for 3 steps). R_f = 0.3 (petroleum ether/EtOAc = 4/1); $[\alpha]_D^{\text{[illegible]}}$ = + 1049 (c = 0.307 in CH_2Cl_2); + 1093 (c = 0.14 in CH_2Cl_2); + 1126 (c = 0.105 in $CHCl_3$); ^{1}H NMR (400 MHz, $CDCl_3$) δ 9.46 (s, 1H), 6.82 (dd,

J = 8.0, 2.8 Hz, 1H), 6.00 (d, J = 8.4 Hz, 1H), 4.05 (t, J = 8.4 Hz, 1H), 3.81–3.73 (m, 1H), 3.16 (dd, J = 18.4, 6.0 Hz, 1H), 2.89 (sept, J = 6.8 Hz, 1H), 2.70 (dd, J = 15.6, 7.6 Hz, 1H), 2.56 (bd, J = 18.4 Hz, 1H), 2.42 (td, J = 13.2, 4.4 Hz, 1H), 2.25 (dd, J =15.6, 9.2 Hz, 1H), 1.82 (dt, J =13.6, 4.0 Hz, 1H), 1.61 (td, J = 12.8, 4.4 Hz, 1H), 1.41 (dt, J = 14.0, 4.0 Hz, 1H), 1.04 (d, J = 6.8 Hz, 3H), 0.96 (d, J = 6.8 Hz, 3H), 0.96 (s, 3H), 0.94 (s, 3H); ^{13}C NMR (101 MHz, $CDCl_3$) δ 194.2, 153.7, 144.3, 143.7, 140.7, 138.3, 120.1, 81.1, 74.4, 50.1, 47.9, 37.6, 34.8, 33.6, 29.7, 26.8, 26.5, 21.7, 21.3, 16.4. HRMS (ESI) m/z calcd for $C_{20}H_{28}NaO_3^+$ $[M+Na]^+$: 339.1931, found 339.1924.

2.10 Synthesis of compound 30

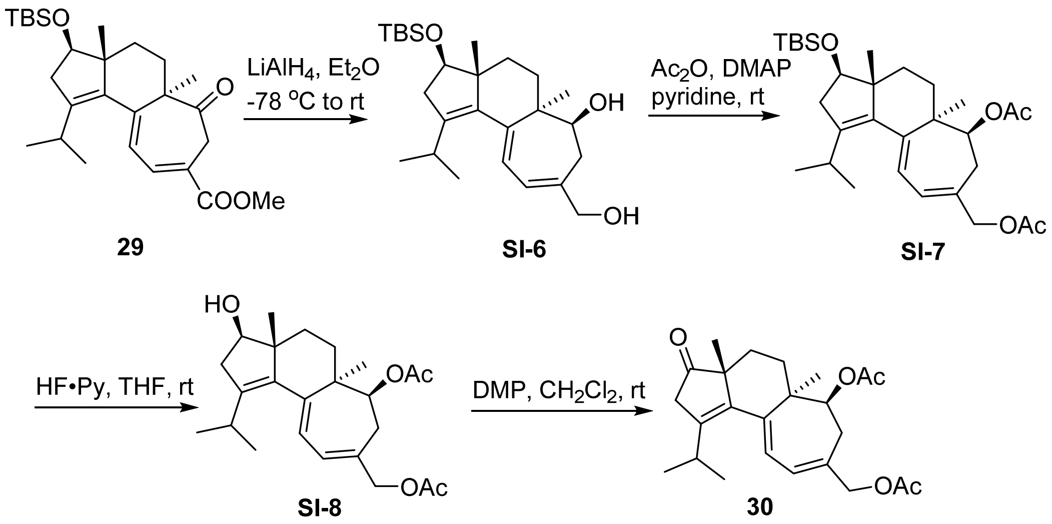

Supplementary Figure 10. Synthesis of compound **30**

To a solution of **29** (175 mg) in Et_2O (8 mL) was added $LiAlH_4$ (86 mg, 2.27 mmol, 6.0 equiv) at -78 °C. The reaction was stirred for 20 min at -78 °C under nitrogen atmosphere and then warmed slowly to room temperature. The solution was quenched by the addition of H_2O (2 mL). Brine (10 mL) and Et_2O (30 mL) was added to the reaction mixture and the organic layer was separated. The aqueous layer was extracted with Et_2O (2 × 10 mL). The combined organic layer was washed with brine (30 mL), dried over Na_2SO_4, and concentrated in vacuo. The filtered and the solvent was evaporated under reduce pressure. The residue was purified by flash chromatography (eluent: petroleum ether/ acetone = 10/1) to give crud compound **SI-6** (112 mg, 68%). R_f = 0.3 (petroleum ether/acetone = 10/3).

To a stirred solution of **SI-6** (112 mg, 0.259 mmol) in pyridine (1.5 mL) containing DMAP (6 mg, 0.2 equiv) was added Ac_2O (0.37 mL, 3.88 mmol, 15 equiv) dropwise at 0 °C under argon. The reaction mixture was stirred at rt for 30 min. The reaction mixture was

poured into sodium bicarbonate solution (10 mL) and extracted with ethyl acetate (3 × 15 mL). The combined organic layer was washed with brine (4 × 15 mL), dried over Na_2SO_4 and evaporated to dryness. The resulting crude residue was purified by flash column chromatography (eluent: petroleum ether/EtOAc = 50/1→10/1) to afford **SI-7** (118 mg, 88%). R_f = 0.3 (petroleum ether/EtOAc = 8/1).

To a stirred solution of **SI-7** (118 mg, 0.228 mmol) in THF (3 mL) was added HF·Py (75%, 0.9 mL) dropwise at 0 °C under argon, and the reaction mixture was stirred at rt for 2 h. The reaction mixture was poured into sodium bicarbonate solution (10 mL) and extracted with ethyl acetate (3 × 20 mL). The combined organic layer was washed with sodium bicarbonate solution (5 × 15 mL) and brine (15 mL), dried over Na_2SO_4 and evaporated to dryness. The resulting crude residue was purified by flash column chromatography (eluent: petroleum ether/acetone = 50/1→10/1) to afford **SI-8** (90 mg, 98%). R_f = 0.3 (petroleum ether/acetone = 4/1).

To a stirred solution of **SI-8** (90 mg, 0.223 mmol) in CH_2Cl_2 (3 mL) was added DMP (156 mg, 1.6 equiv) at rt under argon. The reaction mixture was stirred for 30 min. The reaction mixture was quenched by addition of saturated aqueous Na_2SO_3/$NaHCO_3$. The organic phase was separated, and the aqueous layer was extracted with CH_2Cl_2 (2 × 10 mL). The combined organic layer was dried over Na_2SO_4 and concentrated under reduced pressure. Purification of the residue by flash chromatography (eluent: petroleum ether/acetone = 20/1 →10/1) yielded **30** (78 mg, 87%) as a colorless oil. R_f = 0.4 (petroleum ether/acetone = 4/1); $[\alpha]_D$[illegible] = + 362 (c = 0.093 in CH_2Cl_2); ^1H NMR (400 MHz, $CDCl_3$) δ 5.99 (d, J = 7.6 Hz, 1H), 5.66 (d, J = 7.6 Hz, 1H), 4.81–4.77 (m, 1H), 4.57 (d, J = 12.4 Hz, 1H), 4.48 (d, J = 12.4 Hz, 1H), 3.04 (d, J = 22.8 Hz, 1H), 3.01 (sept, J = 6.8 Hz, 1H), 2.93 (d, J = 22.8 Hz, 1H), 2.75 (dd, J = 17.2, 6.8 Hz, 1H), 2.58 (d, J = 17.6 Hz, 1H), 2.12–2.01 (m, 1H), 2.08 (s, 3H), 2.04 (s, 3H), 1.69–1.62 (m, 2H), 1.39 (dt, J = 13.6, 3.6 Hz, 1H), 1.07 (d, J = 6.8 Hz, 3H), 1.04 (s, 3H), 1.02 (s, 3H), 0.97 (s, 3H). ^{13}C NMR (151 MHz, $CDCl_3$) δ 220.6, 170.9, 170.7, 141.4, 137.9, 134.1, 124.8, 121.6, 76.4, 69.8, 53.4, 45.4, 39.8, 31.2, 30.9, 28.7, 26.4, 25.9, 21.8, 21.2, 21.1, 21.1, 19.7. HRMS (ESI) m/z calcd for $C_{24}H_{32}NaO_5^+$ $[M+Na]^+$: 423.2142, found 423.2143.

2.11 Synthesis of Glaucopine C (3)

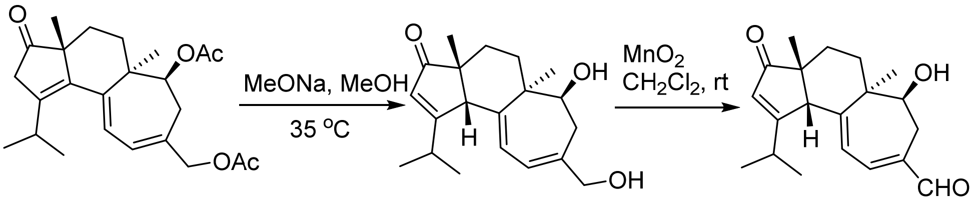

30 **31** **Glaucopine C (3)**

Supplementary Figure 11. Synthesis of compound glaucopine C (**3**)

To a stirred solution of **30** (20 mg, 0.05 mmol) in MeOH (1.3 mL) was added MeONa (30% w/w in MeOH, 0.05 mL) dropwise at rt under argon. The reaction mixture was stirred at 35 °C for 4 h. The reaction mixture was poured into brine (10 mL) and extracted with ethyl acetate (3 × 10 mL). The combined organic layer was dried over Na_2SO_4 and evaporated to dryness. The resulting crude residue was purified by flash column chromatography (eluent: petroleum ether/CH_2Cl_2/acetone = 3/1/1) to afford **31** (11.5 mg, 73%). R_f = 0.3 (petroleum ether/CH_2Cl_2/acetone = 1/1/1). $[\alpha]_D^{23}$ = – 137.2 (c = 0.767 in CHCl3); 1H NMR (500 MHz, CDCl3) δ 6.07–5.99 (m, 3H), 4.12 (d, J = 13.5 Hz, 1H), 4.07 (d, J = 13.5 Hz, 1H), 3.60 (bs, 1H), 3.25 (bd, J = 2.0 Hz, 1H), 2.69–2.57 (m, 2H), 2.48 (dd, J = 19.5, 4.5 Hz, 1H), 1.91–1.85 (m, 1H), 1.77 (td, J = 14.0, 3.0 Hz, 1H), 1.27 (td, J = 14.5, 3.6 Hz, 1H), 1.18 (d, J = 6.5 Hz, 3H), 1.15 (d, J = 6.5 Hz, 3H), 1.09 (s, 3H), 0.78 (3H). 13C NMR (151 MHz, CDCl3) δ 214.8, 190.0, 144.2, 140.3, 126.8, 125.3, 119.9, 78.1, 68.6, 62.7, 49.4, 44.6, 37.2, 37.1, 33.5, 28.9, 27.9, 24.0, 22.6, 20.4. HRMS (ESI) m/z calcd for C20H28NaO3+ $[M+Na]^+$: 339.1936, found 339.1932.

To a solution of **31** in CH_2Cl_2 (1.5 mL) was added MnO_2 (47 mg, 0.54 mmol). The reaction mixture was stirred at room temperature for 3 h. The resulting reaction mixture was filtered to remove insoluble matters and the precipitated was washed with CH_2Cl_2. The organic filtrate was dried over Na_2SO_4, and concentrated under reduced. Flash chromatography of the residue on silica gel (eluent: petroleum ether/EtOAc = 3/1) gave pure Glaucopine C (7 mg, 82%). R_f = 0.3 (petroleum ether/ EtOAc = 1/1). $[\alpha]_D^{23}$ = – 1.95 (*c* = 0.02 in CH_2Cl_2); ^1H NMR (600 MHz, $CDCl_3$) δ 9.46 (s, 1H), 6.80 (dd, *J* = 7.8, 2.4 Hz, 1H), 6.37 (d, *J* = 8.4 Hz, 1H), 6.07 (bs, 1H), 3.80–3.76 (m, 1H), 3.40 (bs, 1H), 2.93 (dd, *J* = 19.8, 3.6 Hz, 1H), 2.61 (dt, *J* = 20.4, 2.4 Hz, 1H), 2.57 (sept, *J* = 6.6 Hz, 1H), 1.94 (dt, *J* = 13.8, 3.6 Hz, 1H), 1.82 (td, *J* = 13.8, 2.4 Hz, 1H), 1.77 (dt, *J* = 14.4, 3.6 Hz, 1H), 1.35 (td, *J* = 14.4, 3.0 Hz, 1H), 1.21 (d, *J* = 6.6 Hz, 3H), 1.20 (d, *J* = 6.6 Hz, 3H), 1.13 (s, 3H), 0.79 (s, 3H). ^{13}C NMR (151 MHz, $CDCl_3$) δ 214.0, 194.6, 188.5, 156.4, 142.2, 139.6, 127.4, 124.8, 77.4, 63.3, 49.9, 45.7, 37.5, 33.4, 32.8, 29.1, 28.5, 24.1, 22.7, 20.4. HRMS (ESI) m/z calcd for $C_{20}H_{27}O_3^+$

[M+H]$^+$: 315.1955, found 315.1961.

2.12 Synthesis of Cyrneine B (2)

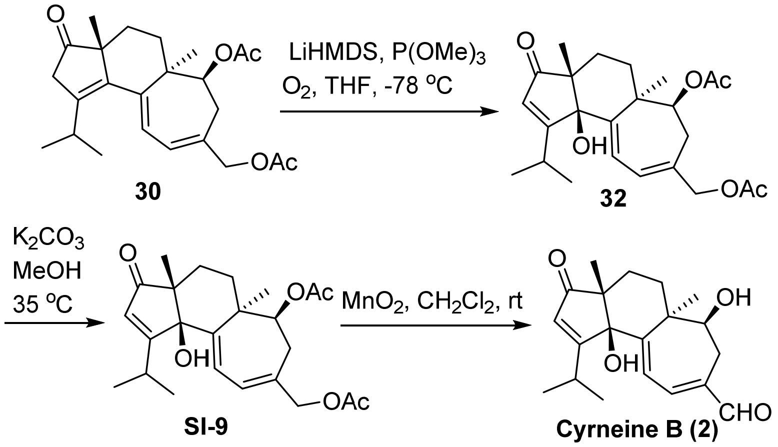

Supplementary Figure 12. Synthesis of compound cyrneine B (**2**)

To a solution of ketone **30** (26 mg, 0.065 mmol) in dry THF (1.2 mL) under argon was added LiHMDS (1 M in THF, 0.13 mL, 0.13 mmol, 2.0 equiv.) at –78 °C and the resulting solution was stirred for 15 min. $P(OMe)_3$ (0.031 mL,0.26 mmol, 4.0 equiv.) was added and the argon atmosphere was exchanged to O_2 (balloon). After 2 h the reaction mixture was quenched by water. The mixture was then warmed to rt and the aqueous phase was extracted three times with ethyl acetate (3 × 10 mL). The combined organic layers were dried over Na_2SO_4 and concentrated in vacuo. The residue was purified by flash chromatography (eluent: petroleum ether/CH_2Cl_2/EtOAc = 6/1/1) to give **32** (16 mg, 58%). R_f = 0.4 (petroleum ether/CH_2Cl_2/ EtOAc = 3/3/2).

To a stirred solution of **32** (16 mg, 0.038 mmol) in MeOH (1.5 mL) was added K_2CO_3 at rt under argon, and the reaction mixture was stirred at 35 °C for 4 h. The reaction mixture was poured into brine (10 mL) and extracted with ethyl acetate (3 × 10 mL). The combined organic layer was dried over Na_2SO_4 and evaporated to dryness. The resulting crude residue was purified by flash column chromatography (eluent: petroleum ether/CH_2Cl_2/acetone = 4/1/1) to afford **SI-9** (7.5 mg, 59%). R_f = 0.3 (petroleum ether/CH_2Cl_2/acetone = 1/1/1).

To a solution of **SI-9** in CH_2Cl_2 (1 mL) was added MnO_2 (30 mg), and the resultant mixture was stirred at room temperature for 3.5 h. The reaction mixture was filtered to remove insoluble materials and the precipitate was washed with CH_2Cl_2. The filtrate was dried over Na_2SO_4, and concentrated under reduced pressure. Flash chromatography of the residue on silica gel (eluent: petroleum ether/acetone = 6/1) gave pure Cyrneine B (6.5 mg, 87%). R_f = 0.2 (petroleum ether/acetone = 3/1). $[\alpha]_D$[illegible] = – 203 (c = 0.37 in CH_2Cl_2); ^{1}H NMR

(600 MHz, $CDCl_3$) δ 9.49 (s, 1H), 7.14 (d, *J* = 9.0 Hz, 1H), 6.96 (dd, *J* = 8.4, 2.4 Hz, 1H), 6.15 (s, 1H), 3.86–3.81 (m, 1H), 2.88 (dd, *J* = 20.4, 3.6 Hz, 1H), 2.69–2.63 (m, 2H), 2.13 (s, 1H), 1.99 (td, *J* = 13.8, 3.0 Hz, 1H), 1.93–1.87 (m, 1H), 1.83–1.77 (m, 1H), 1.66 (d, *J* = 9.0 Hz, 1H), 1.37–1.30 (m, 1H), 1.26 (d, *J* = 6.6 Hz, 3H), 1.17 (d, *J* = 6.6 Hz, 3H), 1.12 (s, 3H), 0.79 (s, 3H). ^{1}H NMR (600 MHz, C_6D_6) δ 9.35 (s), 6.77 (d, J = 8.4 Hz, 1H), 6.30 (dd, *J* = 8.4, 2.4Hz, 1H), 6.06 (s, 1H), 3.35–3.30 (m, 1H), 2.85 (dd, *J* = 20.4, 3.0 Hz, 1H), 2.56 (sept, *J* = 6.6 Hz, 1H), 2.44 (dt, *J* = 20.4, 3.0 Hz, 1H), 2.11–2.01 (m, 2H), 1.85 (s, 1H), 1.55–1.50 (m, 1H), 1.22 (d, 6.6 Hz, 3H), 1.22–1.14 (m, 1H), 1.19 (s, 3H), 0.95 (d, *J* = 6.6 Hz, 3H), 0.40 (s, 3H). ^{13}C NMR (151 MHz, $CDCl_3$) δ 212.1, 194.8, 187.5, 156.4, 142.3, 140.3, 128.5, 122.7, 85.7, 77.2, 54.0, 45.3, 37.0, 33.6, 32.7, 27.3, 26.8, 24.9, 22.3, 19.6. HRMS (ESI) m/z calcd for $C_{20}H_{26}NaO_4^+$ $[M+Na]^+$: 353.1729, found 353.1728.

3. Comparison of Spectroscopic Data of Natural and Synthetic Cyrneine A (1)

Supplementary Table 1. Comparison of the ^{1}H NMR Data of Natural and Synthetic Cyrneine A (1)

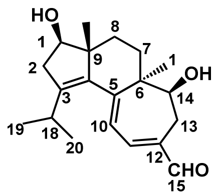

Cyrneine A (1)

Position	δ_H in ppm (mult, J in Hz)		
	Natural[1]	Gademann's Synthesis[2]	This Work
1	3.98 (t, 8.7)	4.05 (t, 8.6)	4.05 (t, 8.4)
2a	2.72 (dd, 15.6, 8.0)	2.71 (dd, 15.6, 8.0)	2.70 (dd, 15.6, 7.6)
2b	2.27 (dd, 15.6, 8.9)	2.26 (dd, 15.6, 8.9)	2.25 (dd, 15.6, 9.2)
7a	2.44 (td, 13.6, 4.5)	2.43 (td, 13.1, 4.3)	2.42 (td, 13.2, 4.4)
7b	1.34 (dt, 14.0, 4.0)	1.40 (td, 14.0, 4.2)	1.41 (dt, 14.0, 4.0)
8a	1.75 (dt, 13.3, 3.8)	1.82 (dt, 13.2, 4.2)	1.82 (dt, 13.6, 4.0)
8b	1.54 (td, 13.1, 4.3)	1.60 (td, 13.1, 4.3)	1.61 (td, 12.8, 4.4)
10	5.93 (d, 8.1)	6.00 (d, 8.1)	6.00 (d, 8.4)
11	6.75 (dd, 8.3, 2.5)	6.82 (dd, 8.2, 2.5)	6.82 (dd, 8.0, 2.8)
13a	3.18 (dd, 18.2, 5.7)	3.16 (dd, 18.3, 5.7)	3.16 (dd, 18.4, 6.0)
13b	2.57 (bd, 18.2)	2.56 (bd, 18.4)	2.56 (bd, 18.4)
14	3.79 (bd, 4.9)	3.77 (m)	3.81-3.72 (m)
15	9.48 (s)	9.46 (s)	9.46 (s)
16	0.89 (s)	0.96 (s)	0.96 (s)
17	0.87 (s)	0.93 (s)	0.94 (s)
18	2.91 (sept, 6.9)	2.89 (sept, 6.8)	2.89 (sept, 6.8)
19	0.89 (d, 6.9)	0.96 (d, 6.9)	0.96 (d, 6.8)
20	0.97 (d, 6.9)	1.04 (d, 6.9)	1.04 (d, 6.8)

Supplementary Table 2. Comparison of the ^{13}C NMR Data of Natural and Synthetic Cyrneine A (1)

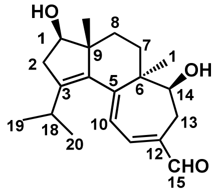

Cyrneine A (1)

Position	δC in ppm		
	Natural[1]	Gademann's Synthesis[2]	This work
1	81.3	81.0	81.1
2	37.6	37.4	37.6
3	143.9	143.6	143.7
4	140.9	132.1	140.7
5	153.9	153.5	153.7
6	48.0	48.0	47.9
7	33.7	33.5	33.6
8	34.9	34.6	34.8
9	50.3	50.0	50.1
10	120.3	120.0	12.01
11	144.7	144.1	144.3
12	138.5	138.1	138.3
13	29.7	29.5	29.7
14	74.6	74.5	74.4
15	194.5	194.1	194.2
16	26.7	26.3	26.5
17	16.6	16.3	16.4
18	26.9	26.6	26.8
19	21.9	21.6	21.7
20	21.4	21.2	21.3

Supplementary Table 3. Comparison of the ^{1}H NMR Data of Natural and Synthetic Cyrneine B (2)

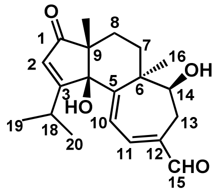

Cyrneine B (2)

Position	δ_H in ppm (mult, J in Hz)	
	Natural[1]	This Work
2	6.16 (s)	6.15 (s)
7a	1.53 (dt, 14.8, 4.0)[a]	1.55–1.50 (m)[a]
7b	1.4–1.2 (m)[a]	1.22-1.14[a]
8	2.1–2.0 (m)[a]	2.11–2.01 (m)[a]
10	6.97 (dd, 8.4, 2.2)	7.14 (d, 9.0)[b]
11	7.15 (d, 8.3)	6.96 (dd, 8.4, 2.4)[b]
13a	2.85 (dd, 20.4, 3.2)[a]	2.85 (dd, 20.4, 3.0)[a]
13b	2.41 (dt, 20.1, 3.0)[a]	2.44 (dt, 20.4, 3.0)[a]
14	3.42 (t, 3.2)[a]	3.35–3.30 (m)[a]
15	9.49 (s)	9.49 (s)
16	0.78 (s)	0.79 (s)
17	1.20 (s)	1.12 (s)
18	2.66 (sept, 6.8)[a]	2.56 (sept, 6.6)[a]
19	1.17 (d, 6.8)	1.17 (d, 6.6)
20	1.26 (d, 6.8)	1.26 (d, 6.6)

[a] Values recorded in C_6D_6.

[b] Based on the NOESY and HMBC correlations of our synthesized product, it seemed that the H_{10} and H_{11} were assigned incorrectly in the reference.

Supplementary Table 4. Comparison of the ^{13}C NMR Data of Natural and Synthetic Cyrneine B (2)

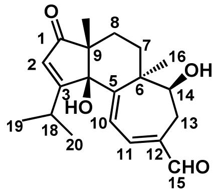

Cyrneine B (2)

Position	δ_C in ppm	
	Natural[1]	This work
1	212.8	212.1
2	127.9	128.5
3	188.0	187.5
4	85.7	85.7
5	156.9	156.3
6	45.3	45.3
7	37.4	37.0
8	32.6	32.7
9	54.3	54.0
10	123.3	122.7
11	142.9	142.3
12	140.5	140.3
13	33.8	33.6
14	77.3	77.2
15	195.2	194.8
16	26.8	26.8
17	20.0	19.6
18	27.3	27.3
19	24.4	24.9
20	22.5	22.3

Supplementary Table 5. Comparison of the ^{1}H-NMR Data of Natural and Synthetic Glaucopine C (3)

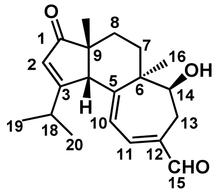

Glaucopine C (3)

Position	δ_H in ppm (mult, J in Hz)	
	Natural[3]	This Work
2	6.06 (bs)	6.07 (bs)
4	3.41 (bs)	3.40 (bs)
7a	1.35 (td, 13.9, 13.9, 2.6)	1.35 (td, 14.4, 3.0)
7b	1.78 (dt, 13.9, 3.5, 3.5)	1.77 (dt, 14.4, 3.6)
8a	1.85 (td, 14.0, 14.0, 2.5)	1.82 (td, 13.8, 2.4)
8b	1.94 (dt, 13.3, 3.3, 3.3)	1.94 (dt, 13.8, 3.6)
10	6.39 (d, 8.2)	6.37 (d, 8.4)
11	6.81(dd, 13.5, 6.6)	6.80 (dd, 7.8, 2.4)
13a	2.61 (dt, 20.2, 2.7, 2.7)	2.61 (dt, 20.4, 2.4)
13b	2.93 (dd, 20.2, 4.0)	2.93 (dd, 19.8, 3.6)
14	3.79 (bs)	3.80–3.76 (m)
15	9.48 (s)	9.46 (s)
16	0.80 (s)	0.79 (s)
17	1.12 (s)	1.13 (s)
18	2.58 (sept, 6.7)	2.57 (sept, 6.6)
19	1.22 (d, 6.7)	1.20 (d, 6.6)
20	1.29 (d, 6.7)	1.21 (d, 6.6)

Supplementary Table 6. Comparison of the ^{13}C NMR Data of Natural and Synthetic Glaucopine C (3)

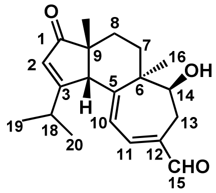

Glaucopine C (3)

Position	δ_C in ppm	
	Natural[3]	This work
1	214.3	214.0
2	127.6	127.4
3	188.9	188.5
4	63.5	63.3
5	156.5	156.4
6	45.8	45.7
7	37.7	37.5
8	33.6	33.4
9	50.1	49.9
10	125.0	124.8
11	142.6	142.2
12	139.8	139.6
13	32.9	32.8
14	77.6	77.2
15	194.9	194.6
16	28.7	28.5
17	22.8	22.7
18	29.7	29.1
19	20.6	24.1[a]
20	20.6	20.4[a]

[a] One of the carbons was incorrectly assigned in the reference.

4. Copies of NMR Spectra

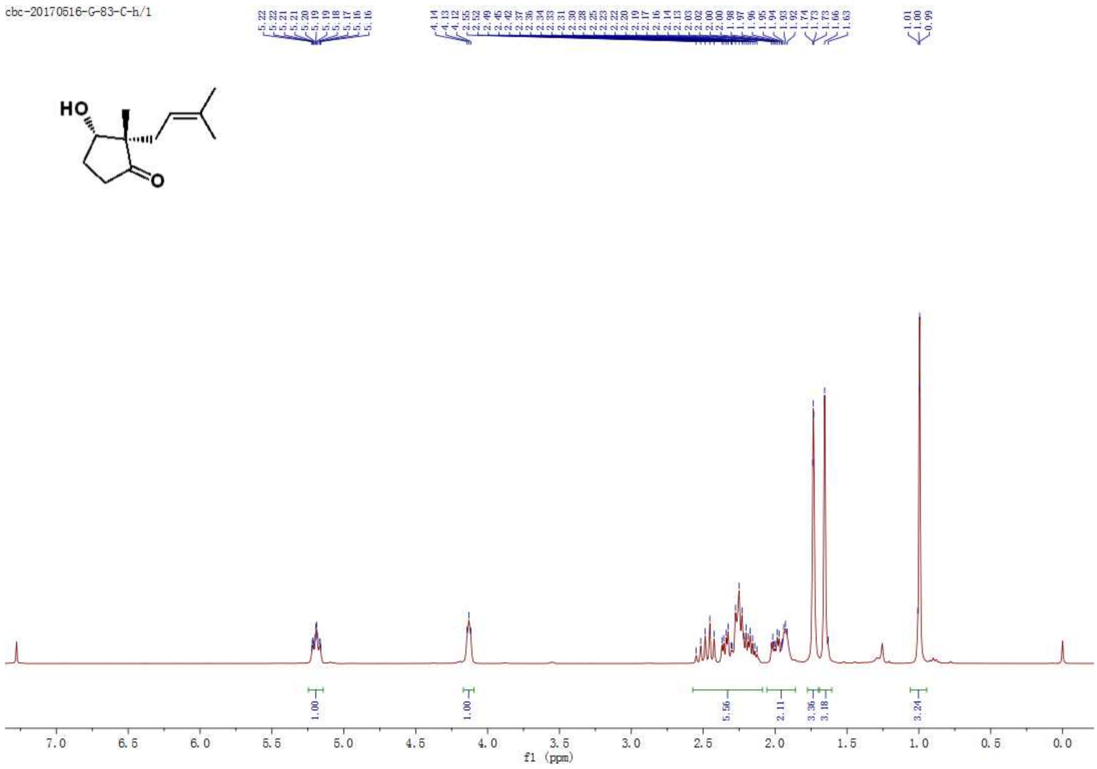

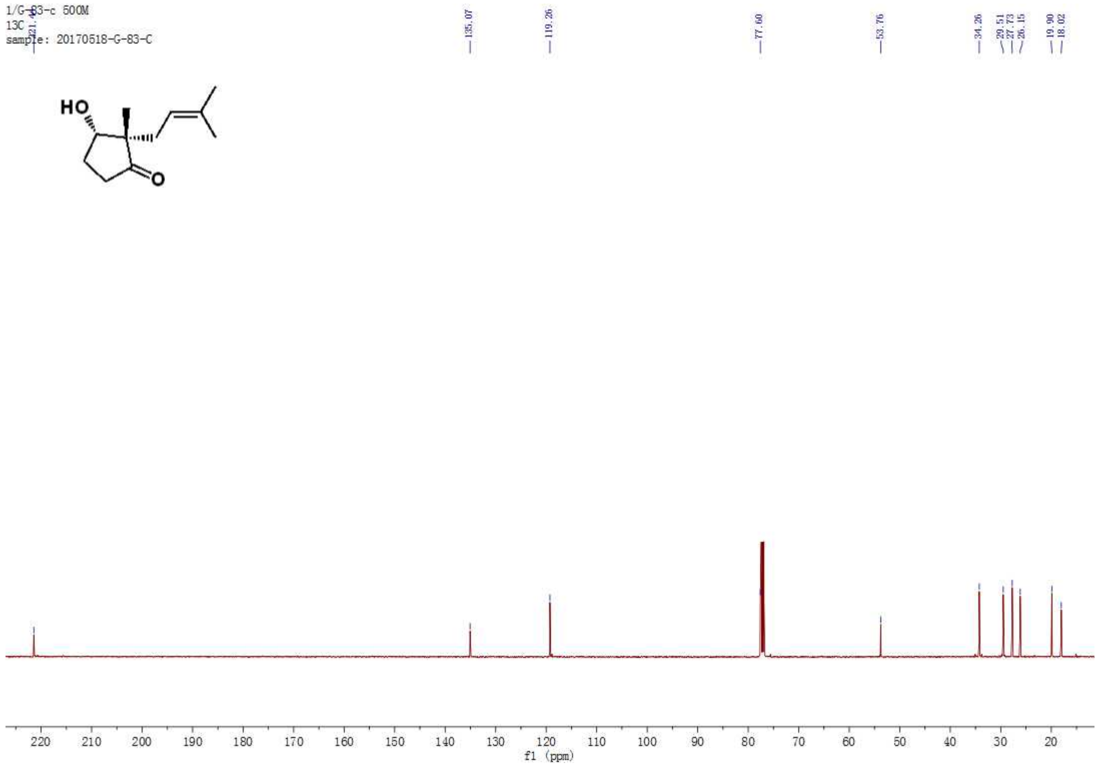

Supplementary Figure 13. ^{1}H NMR and ^{13}C NMR of compound **12**

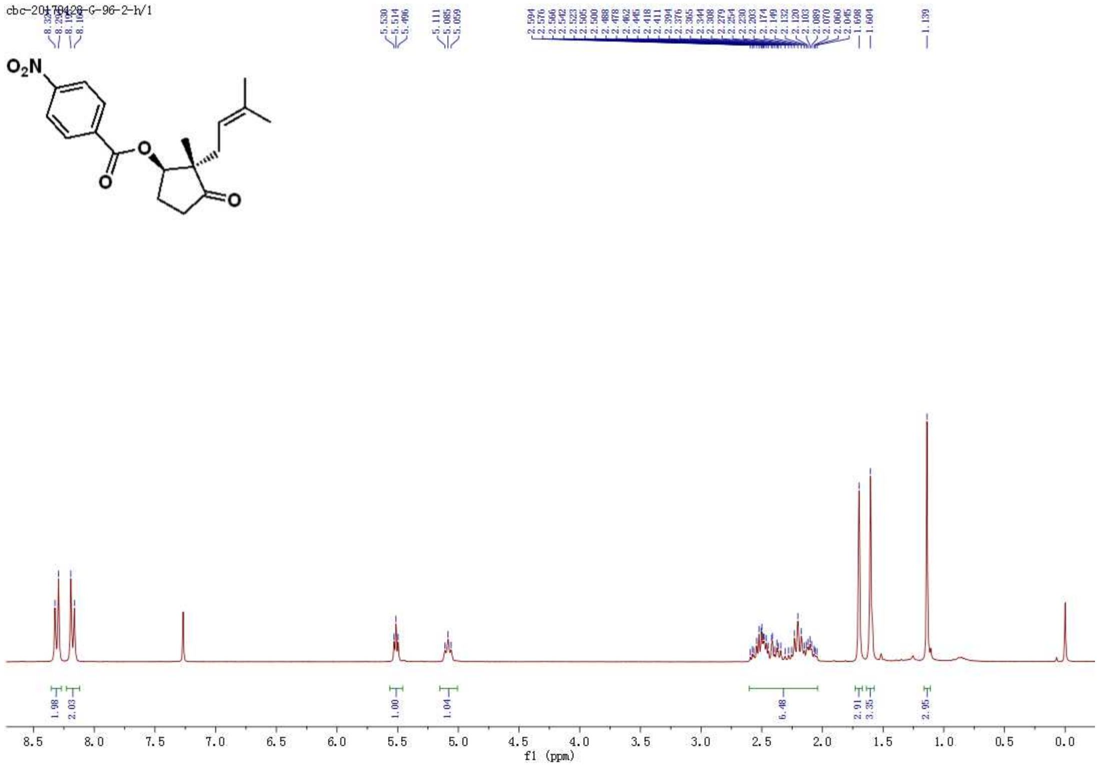

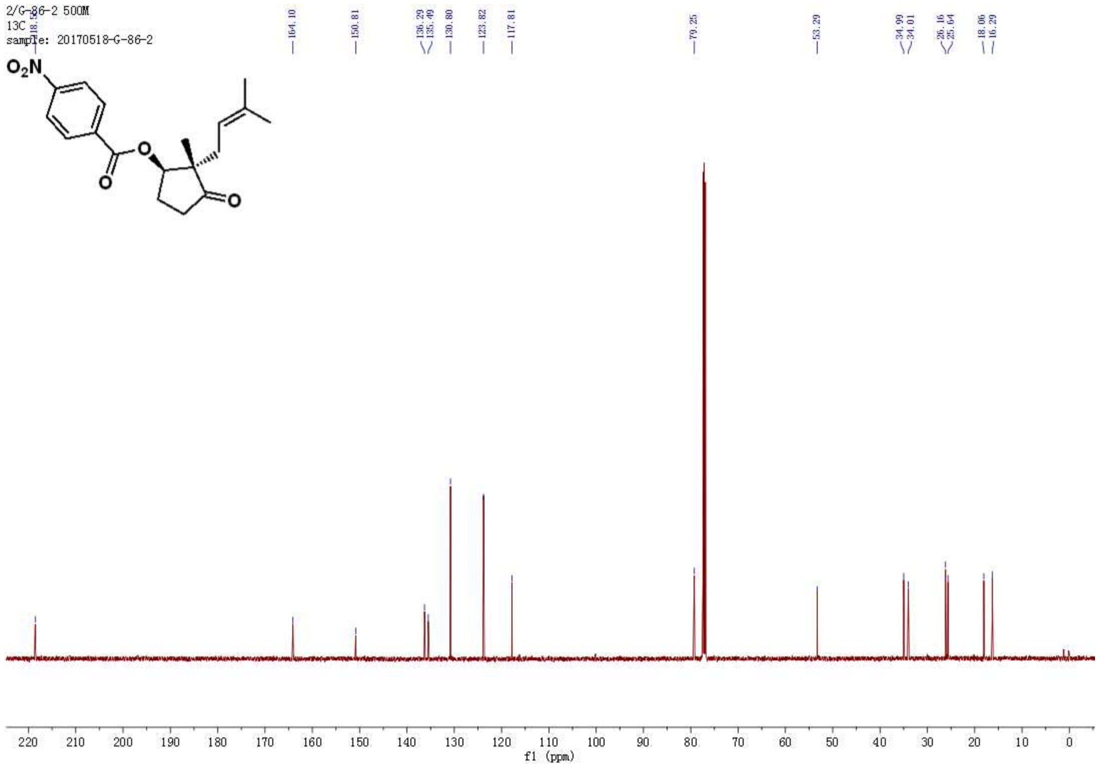

Supplementary Figure 14. ^{1}H NMR and ^{13}C NMR of compound **SI-1**

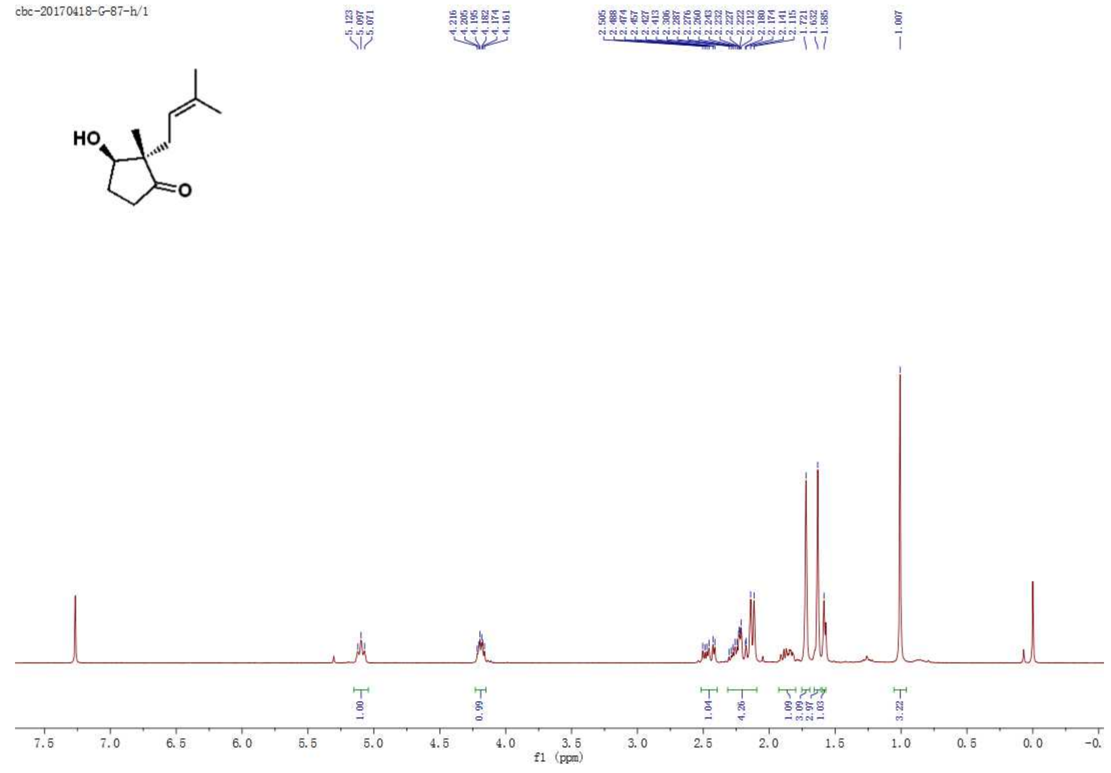

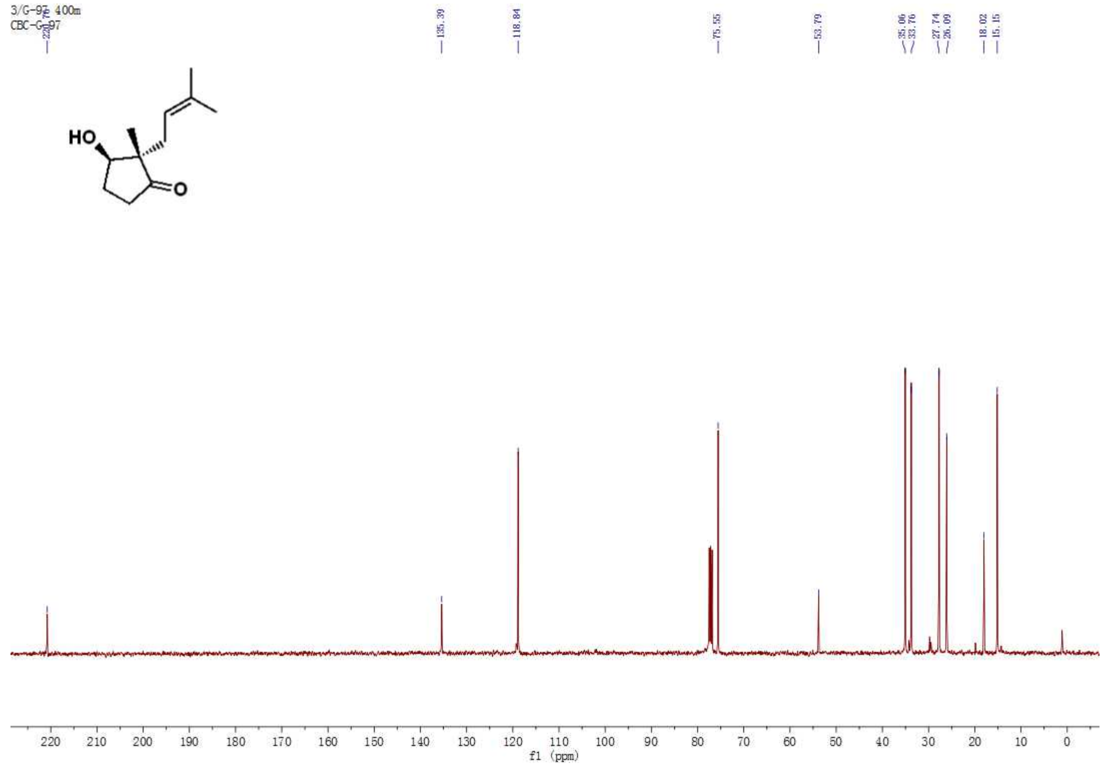

Supplementary Figure 15. ^{1}H NMR and ^{13}C NMR of compound **13**

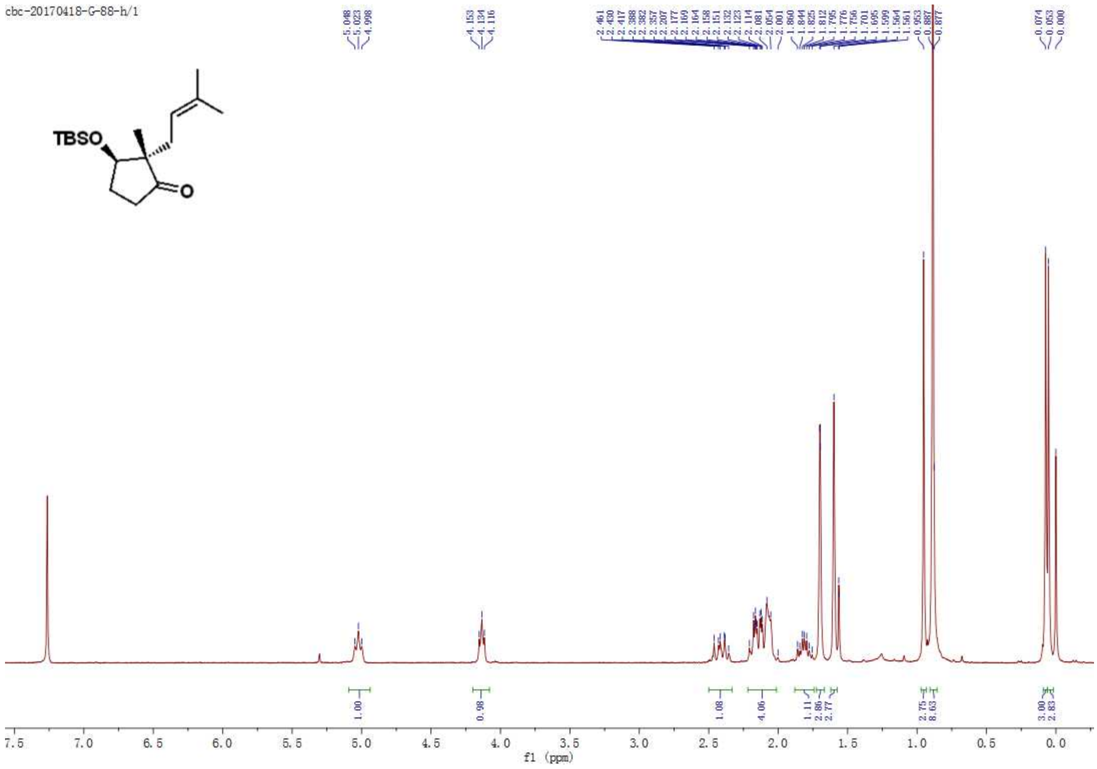

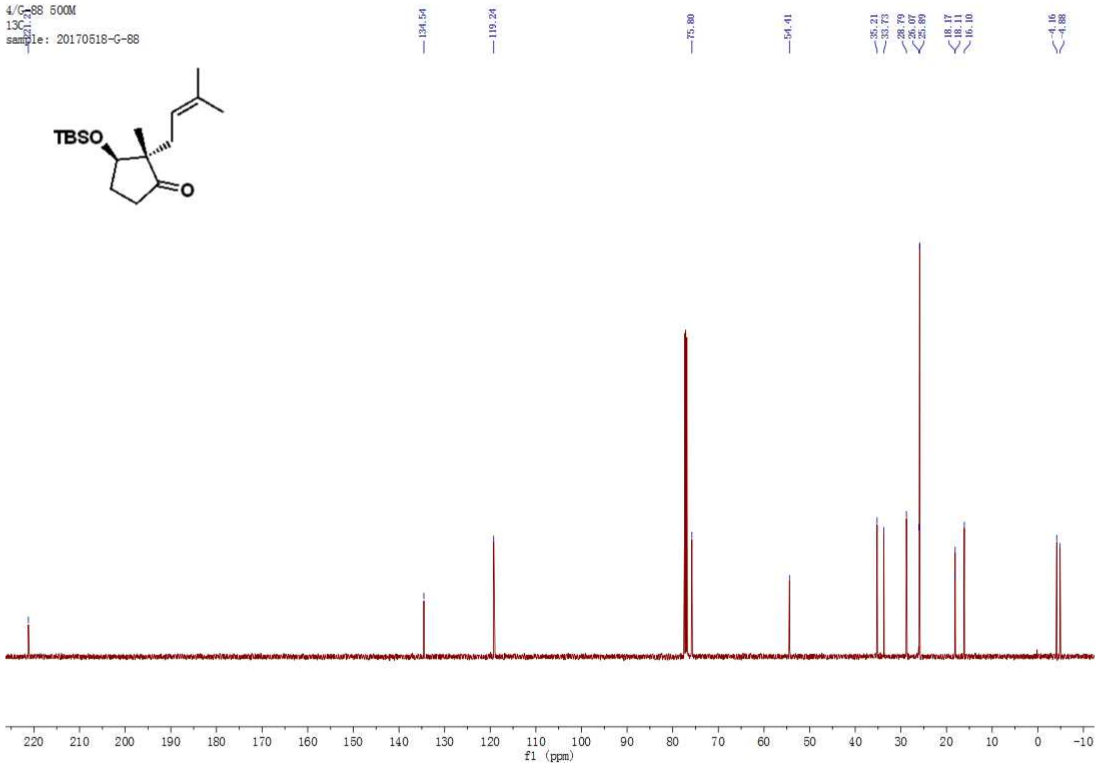

Supplementary Figure 16. ^{1}H NMR and ^{13}C NMR of compound **SI-2**

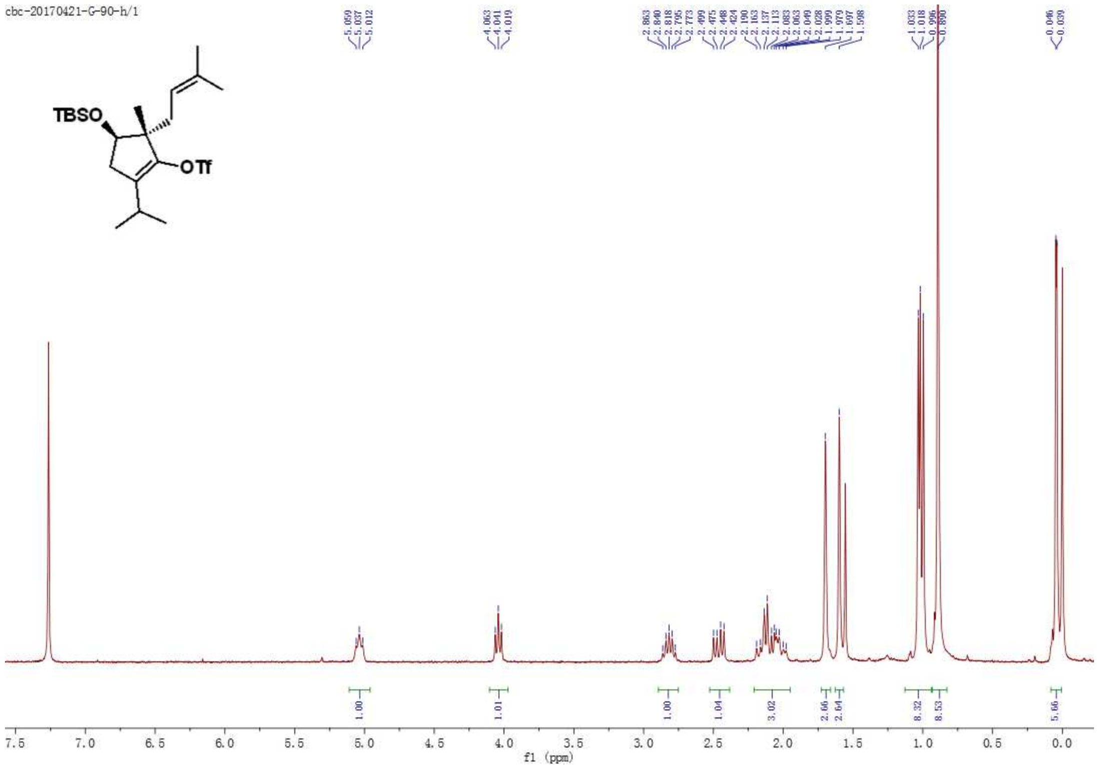

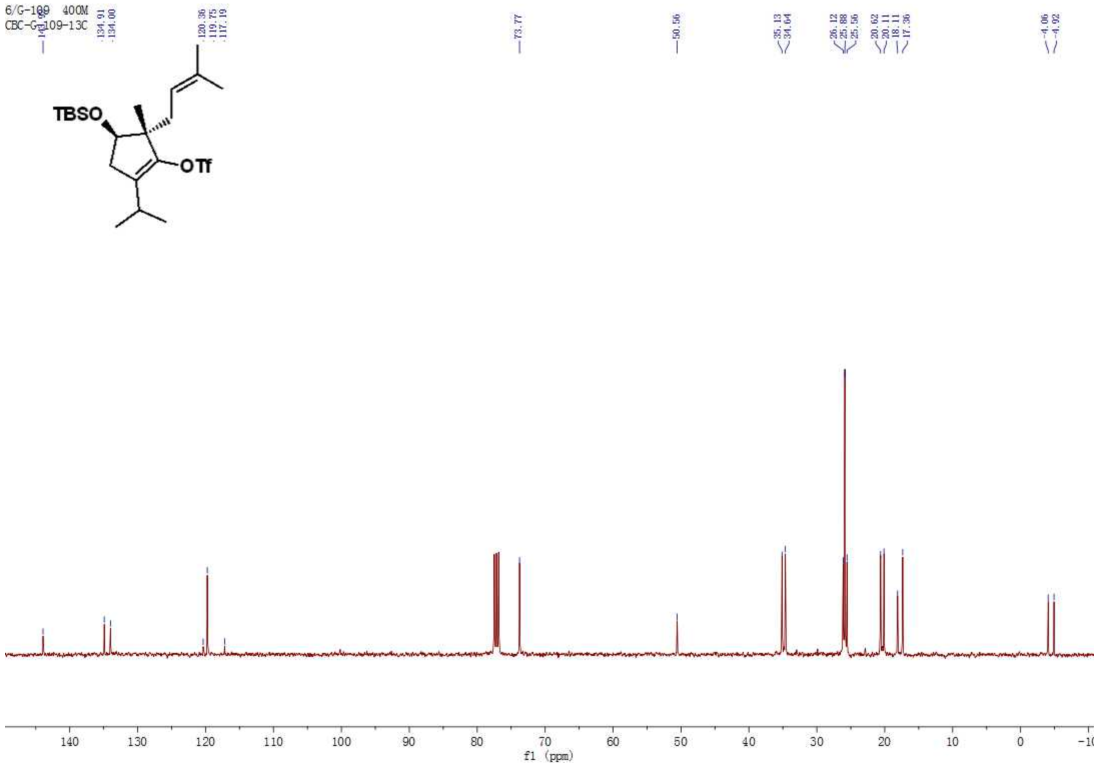

Supplementary Figure 17. ^{1}H NMR and ^{13}C NMR of compound **15a**

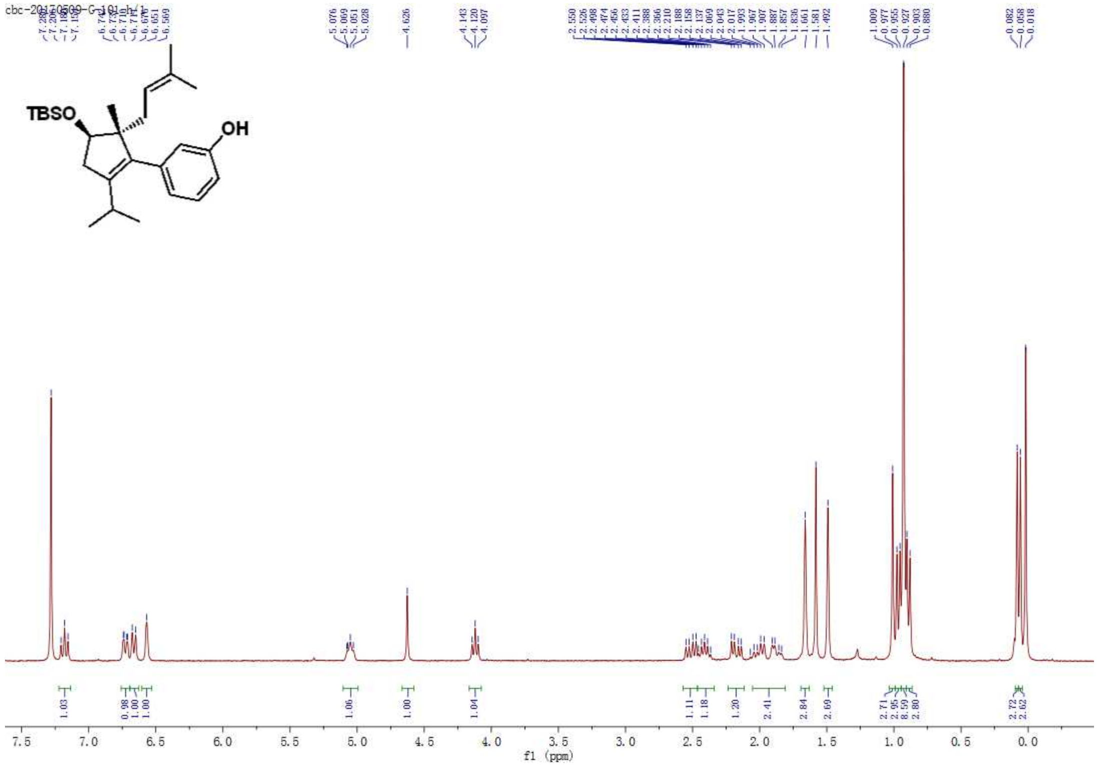

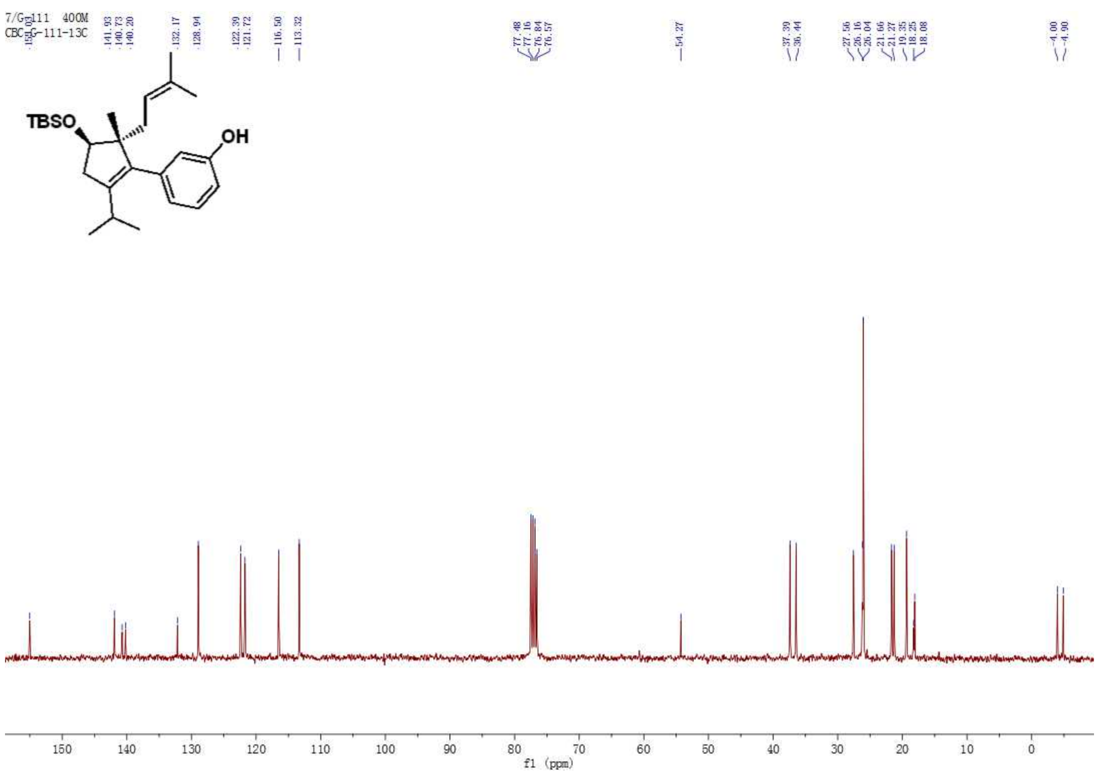

Supplementary Figure 18. ^{1}H NMR and ^{13}C NMR of compound **17a**

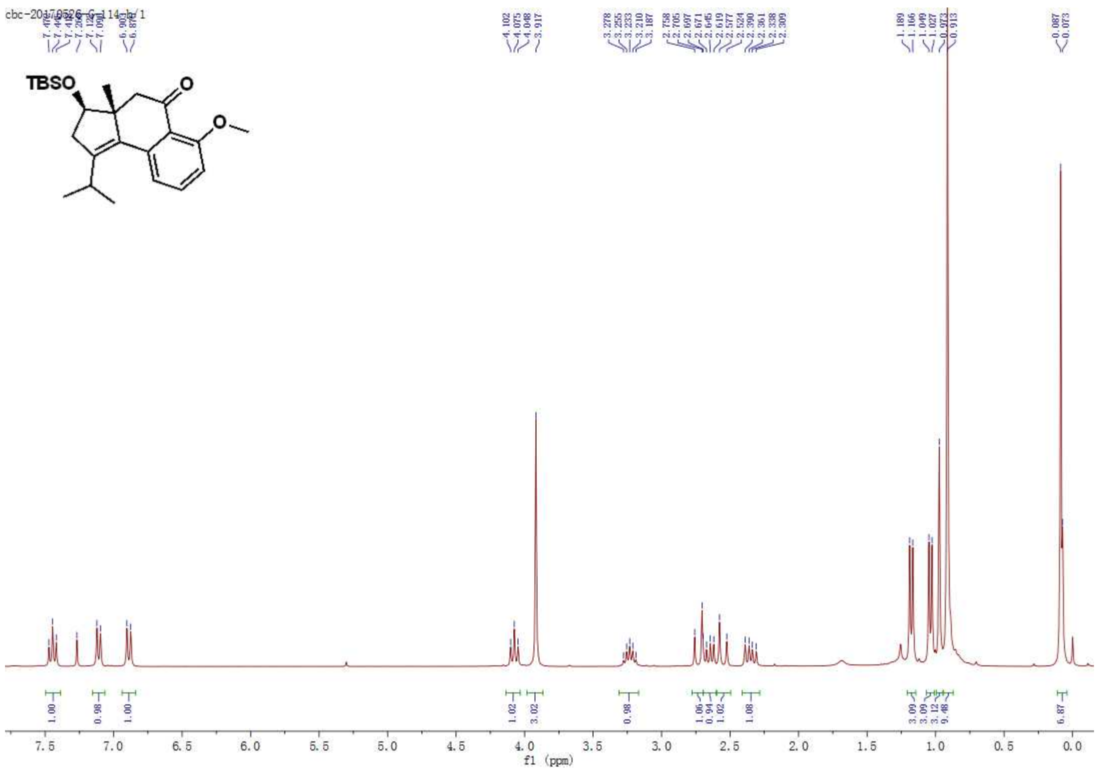

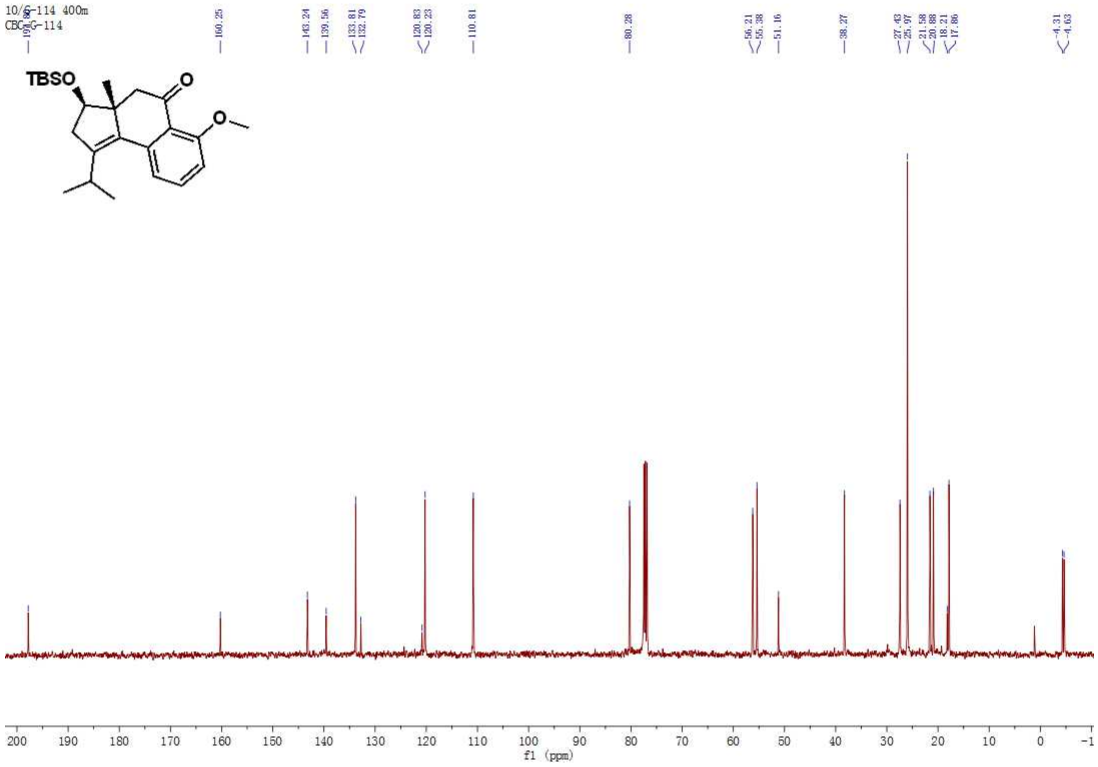

Supplementary Figure 19. ^{1}H NMR and ^{13}C NMR of compound **23**

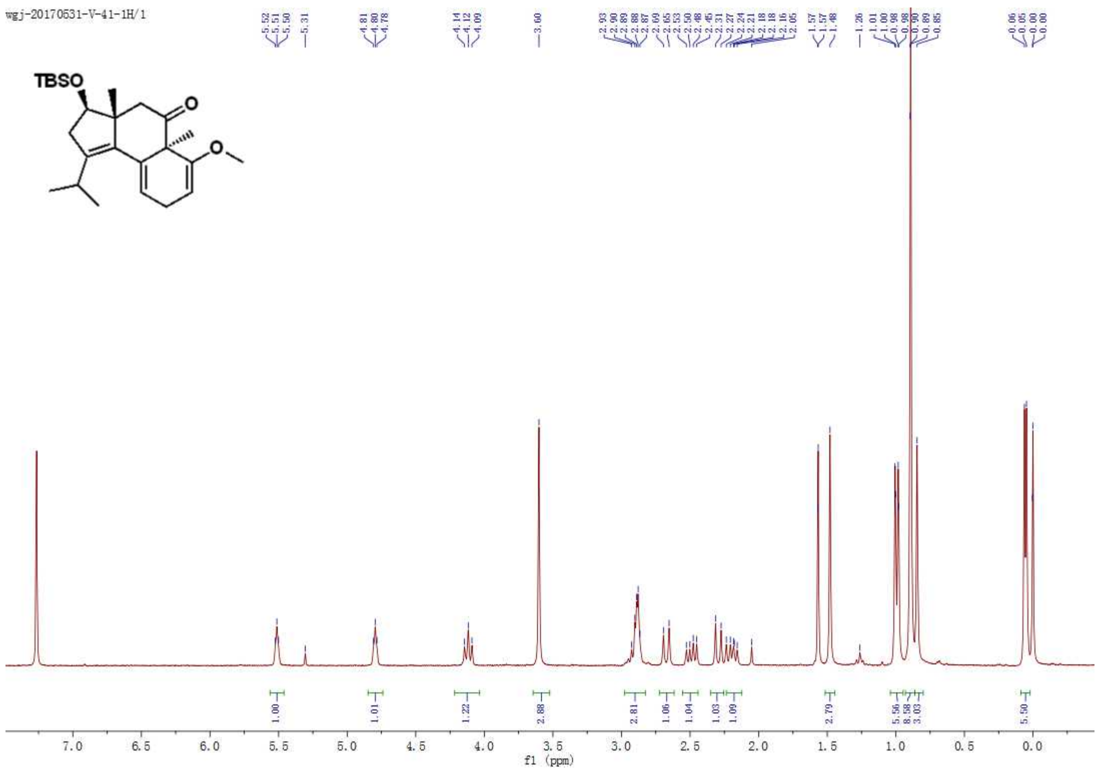

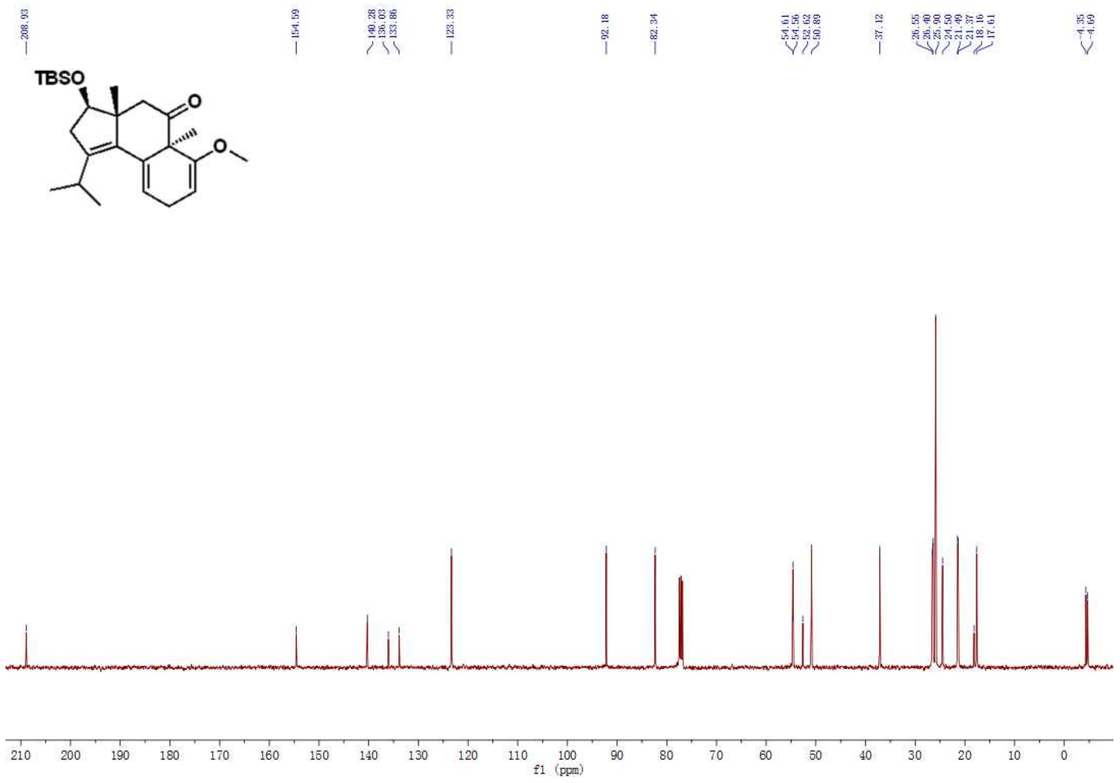

Supplementary Figure 20. ^{1}H NMR and ^{13}C NMR of compound **24**

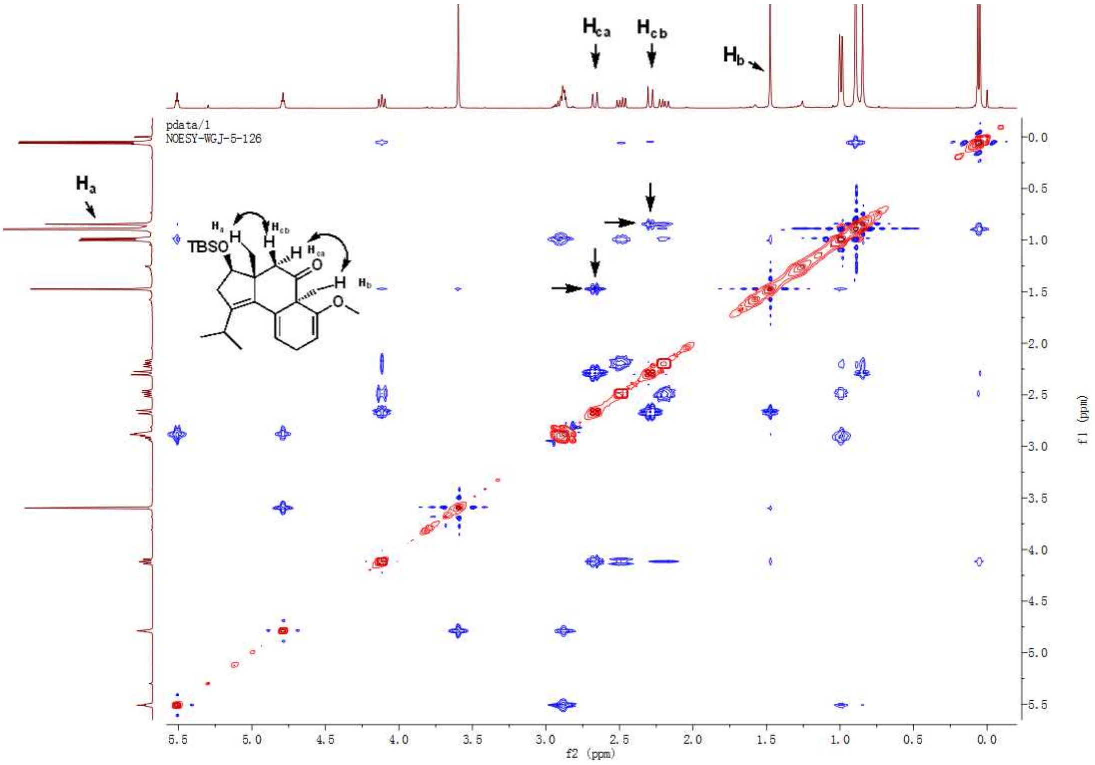

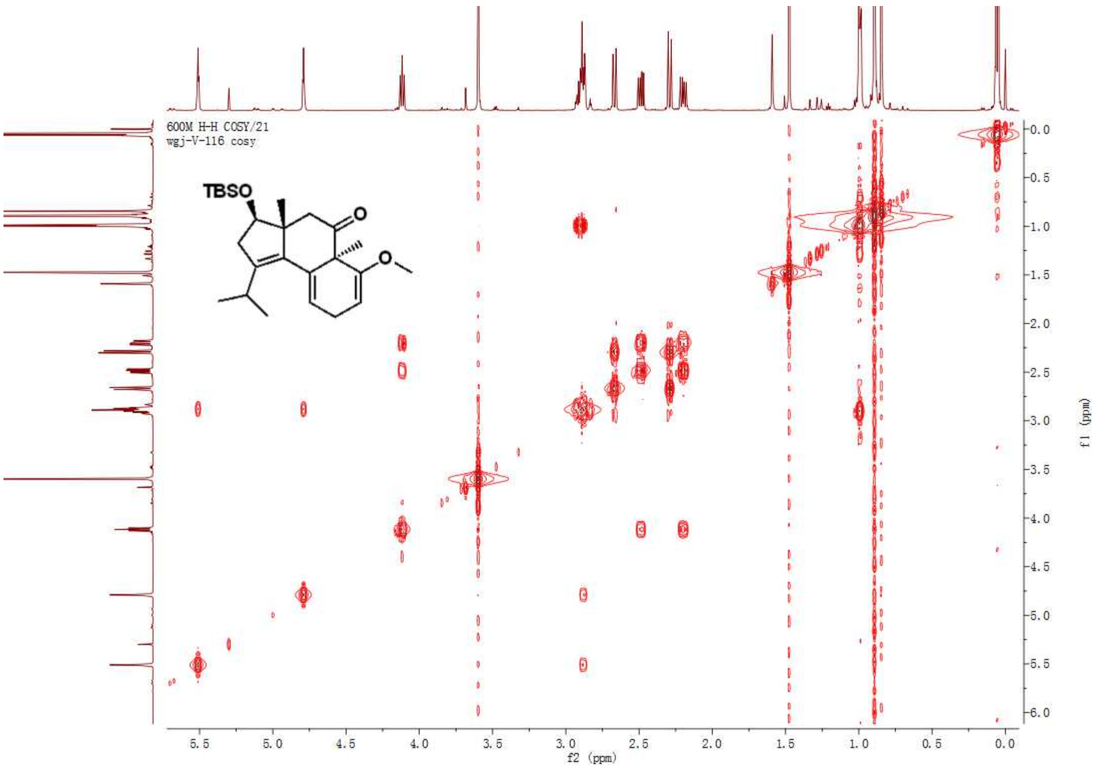

Supplementary Figure 21. NOESY and ^{1}H-^{1}H COSY of compound **24**

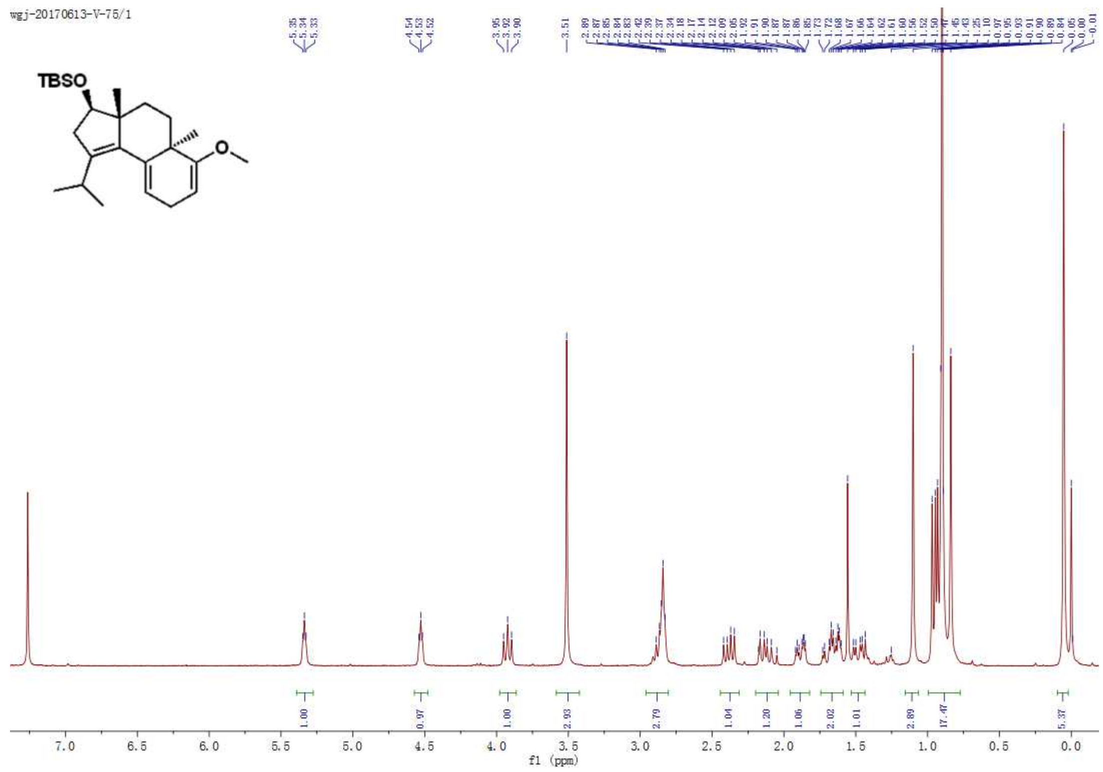

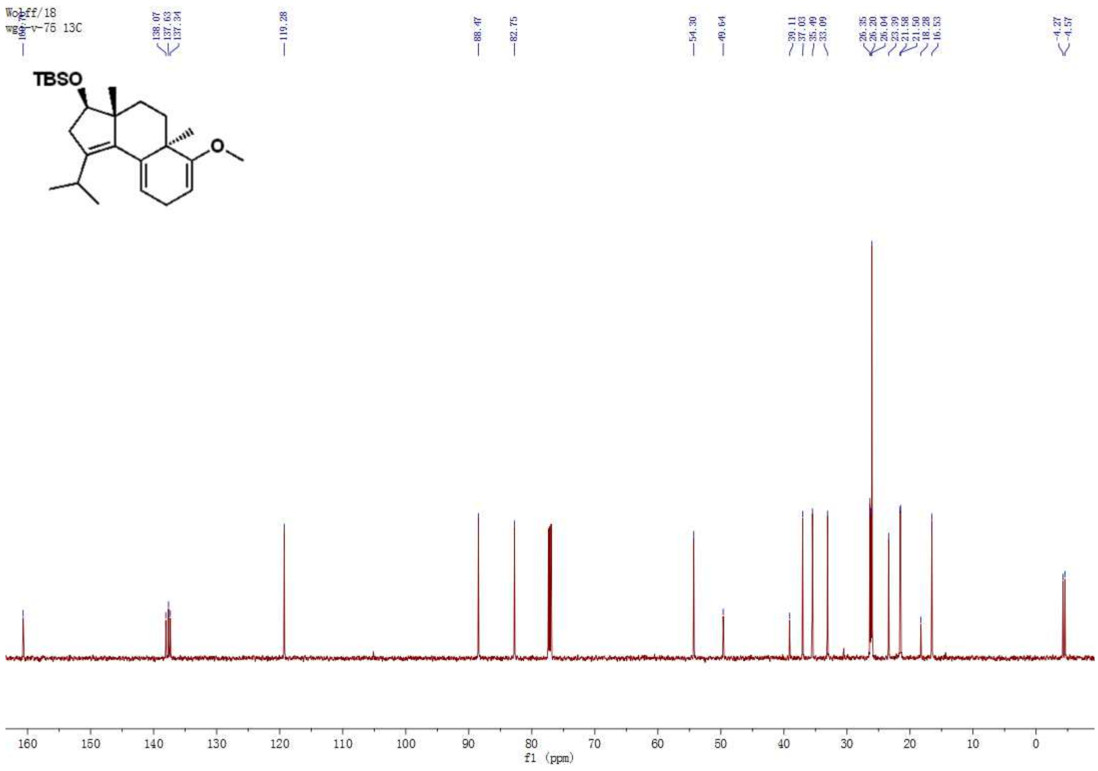

Supplementary Figure 22. ^{1}H NMR and ^{13}C NMR of compound **26**

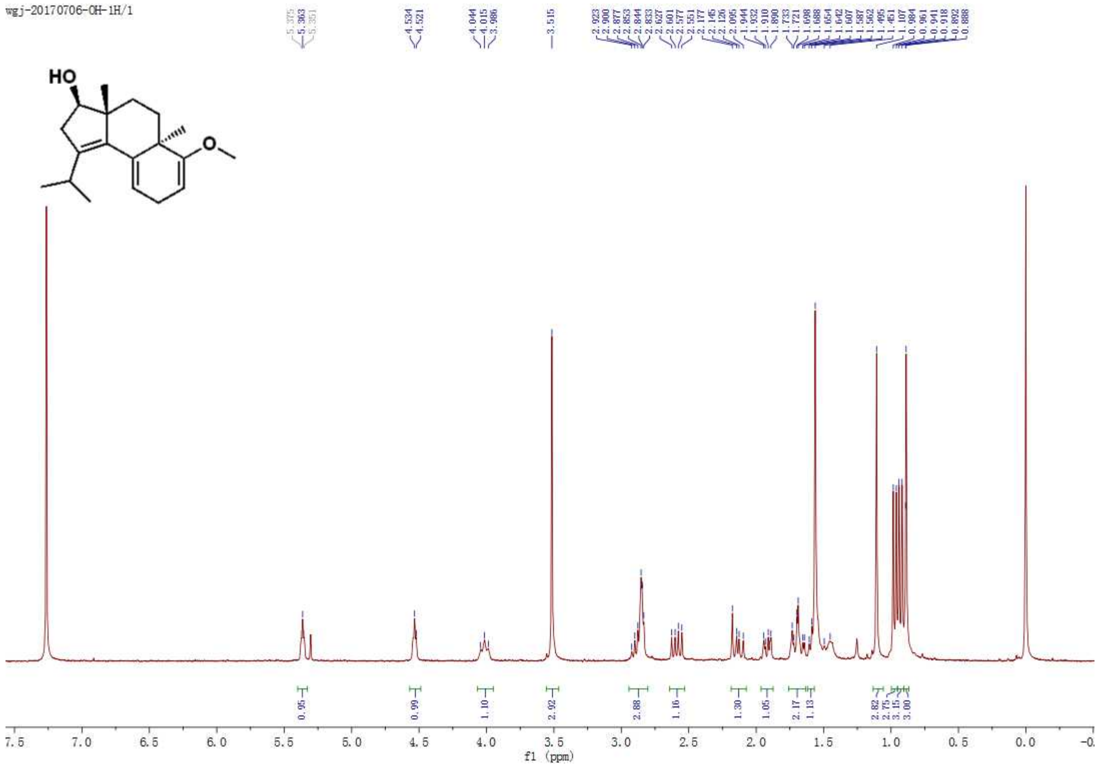

Supplementary Figure 23. ^{1}H NMR of compound **27**

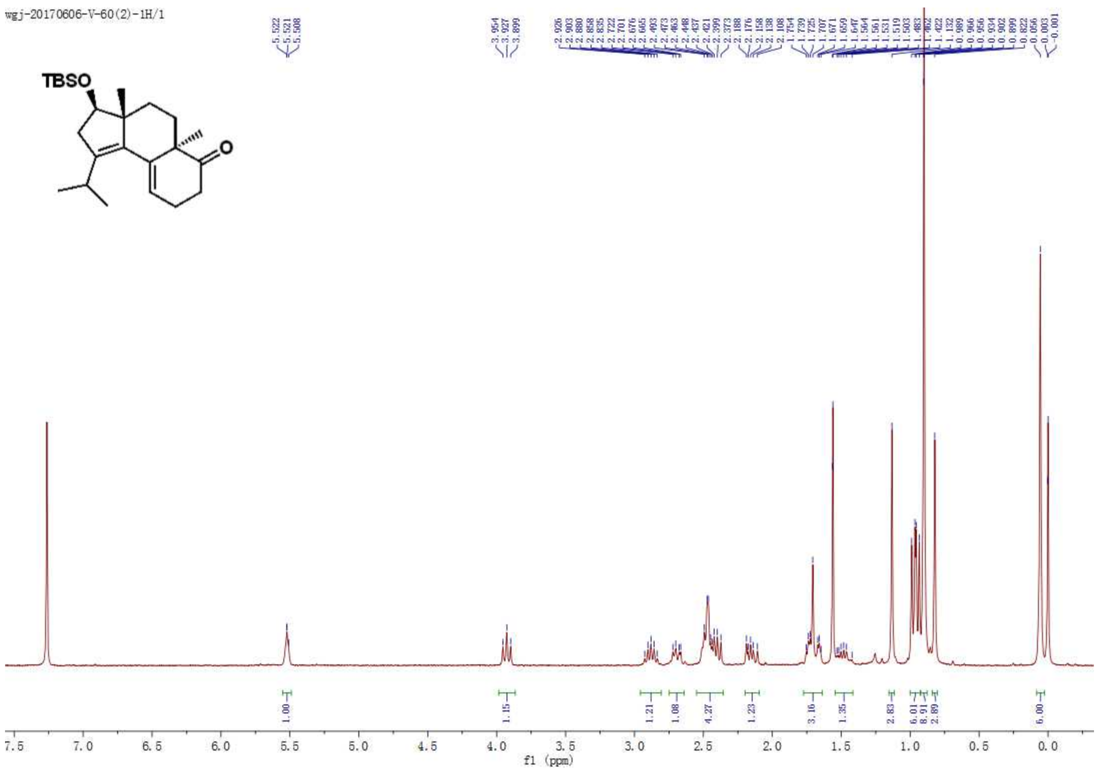

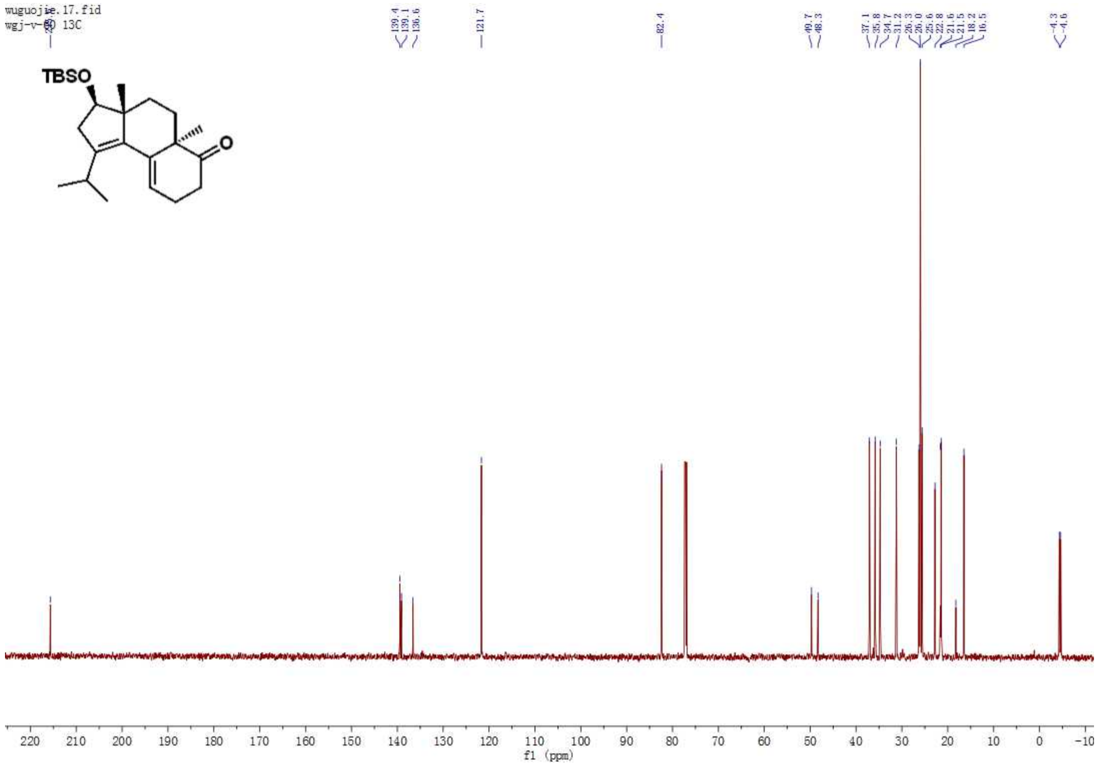

Supplementary Figure 24. ^{1}H NMR and ^{13}C NMR of compound **28**

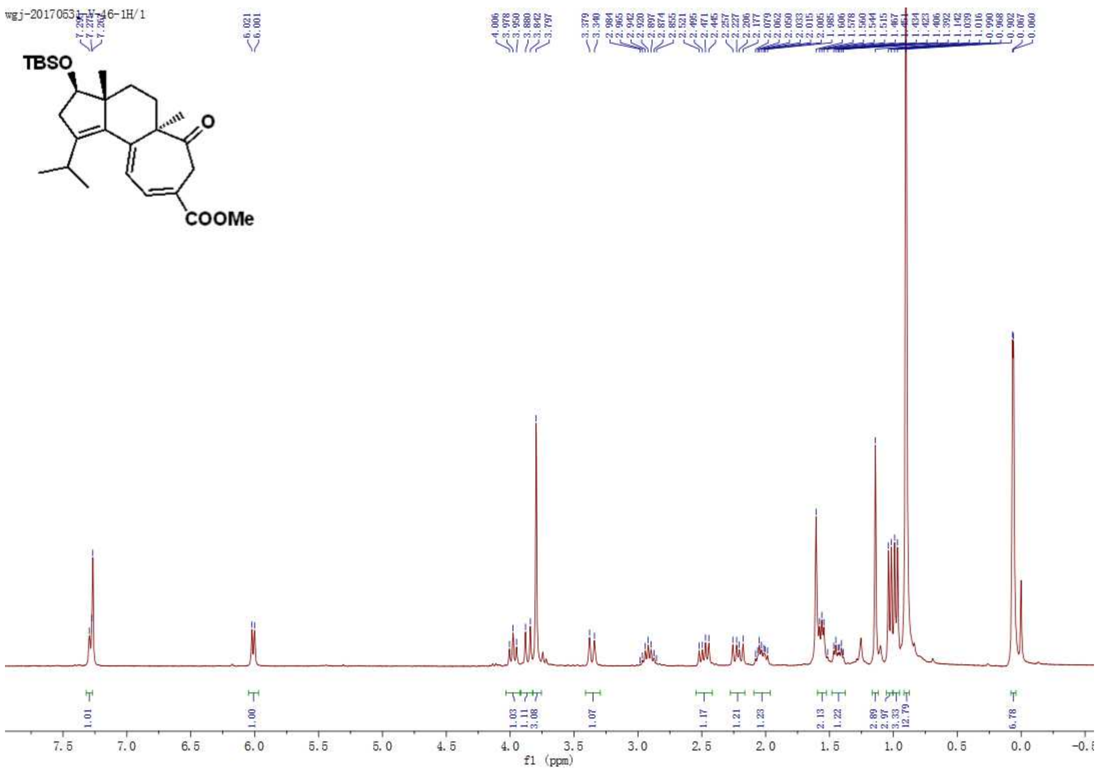

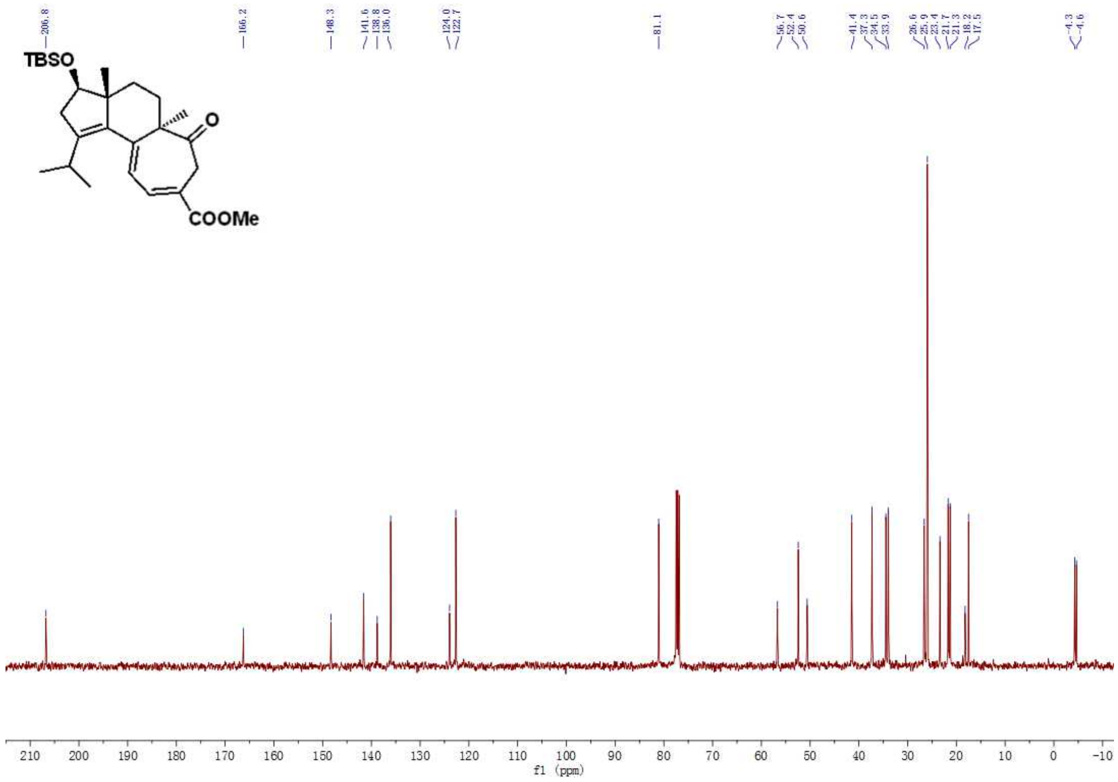

Supplementary Figure 25. ^{1}H NMR and ^{13}C NMR of compound **29**

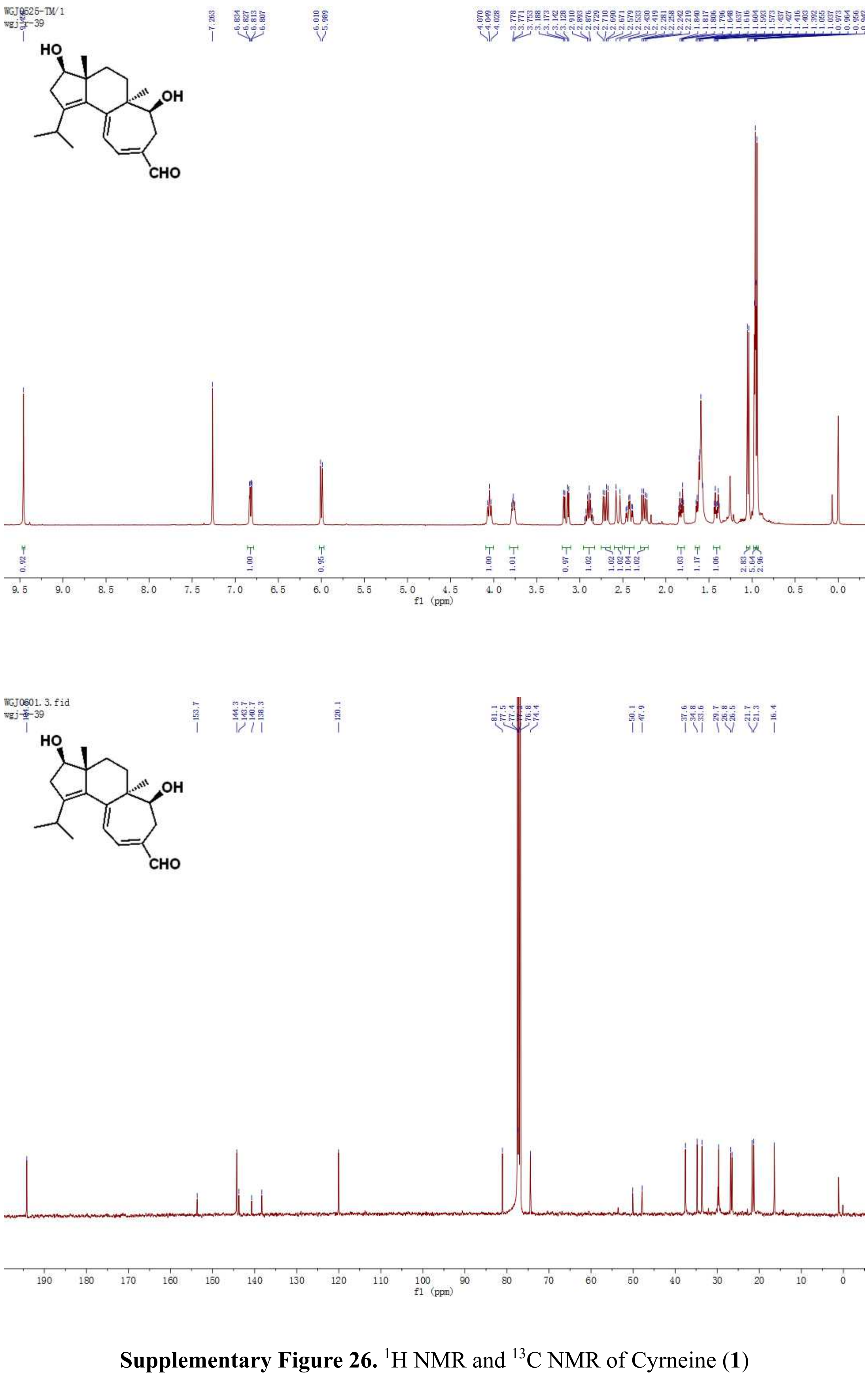

Supplementary Figure 26. ^{1}H NMR and ^{13}C NMR of Cyrneine (**1**)

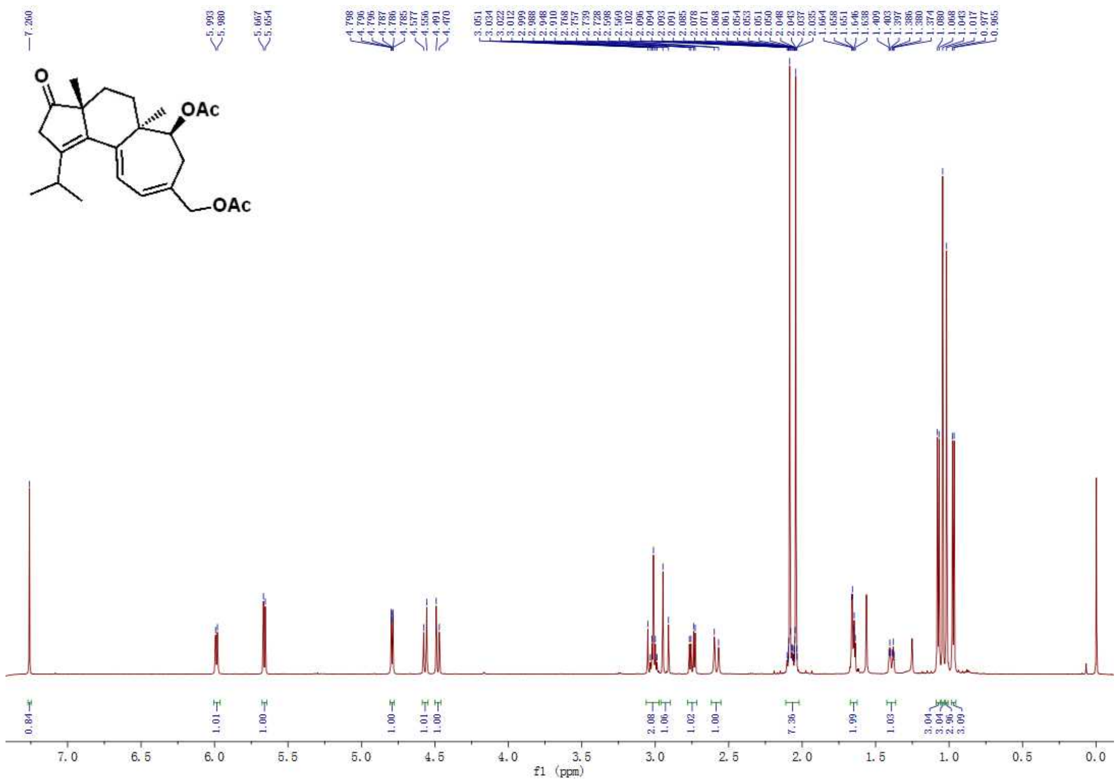

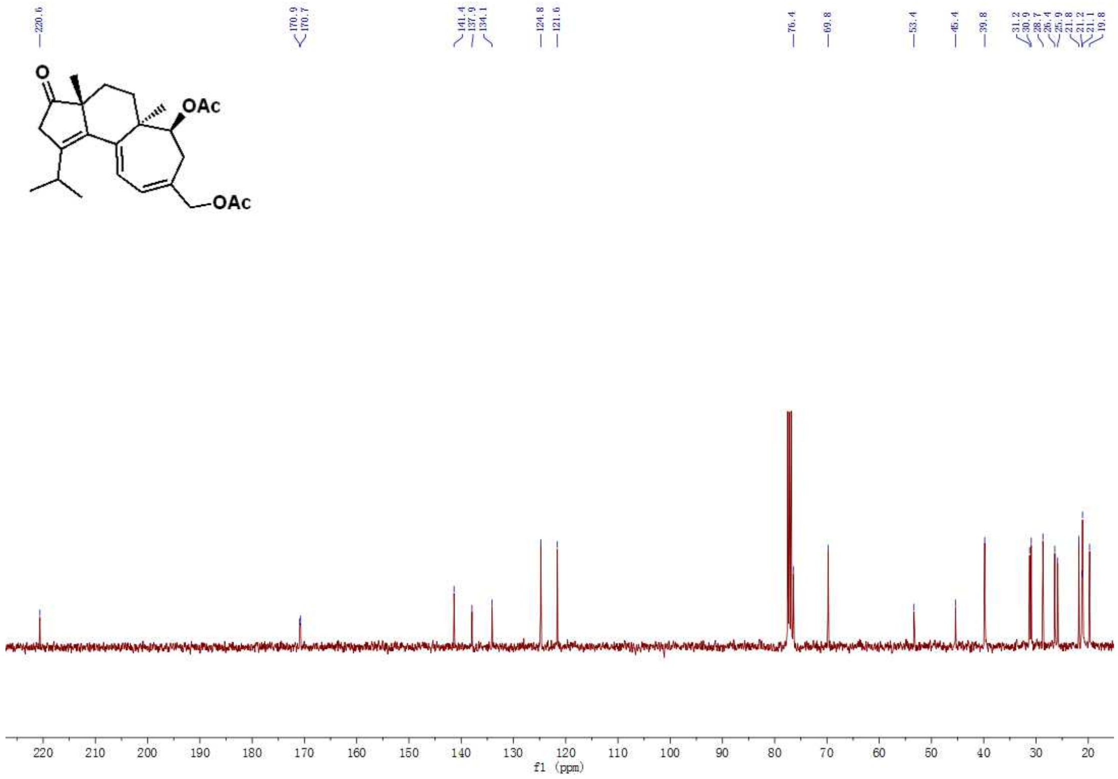

Supplementary Figure 27. ^{1}H NMR and ^{13}C NMR of compound **30**

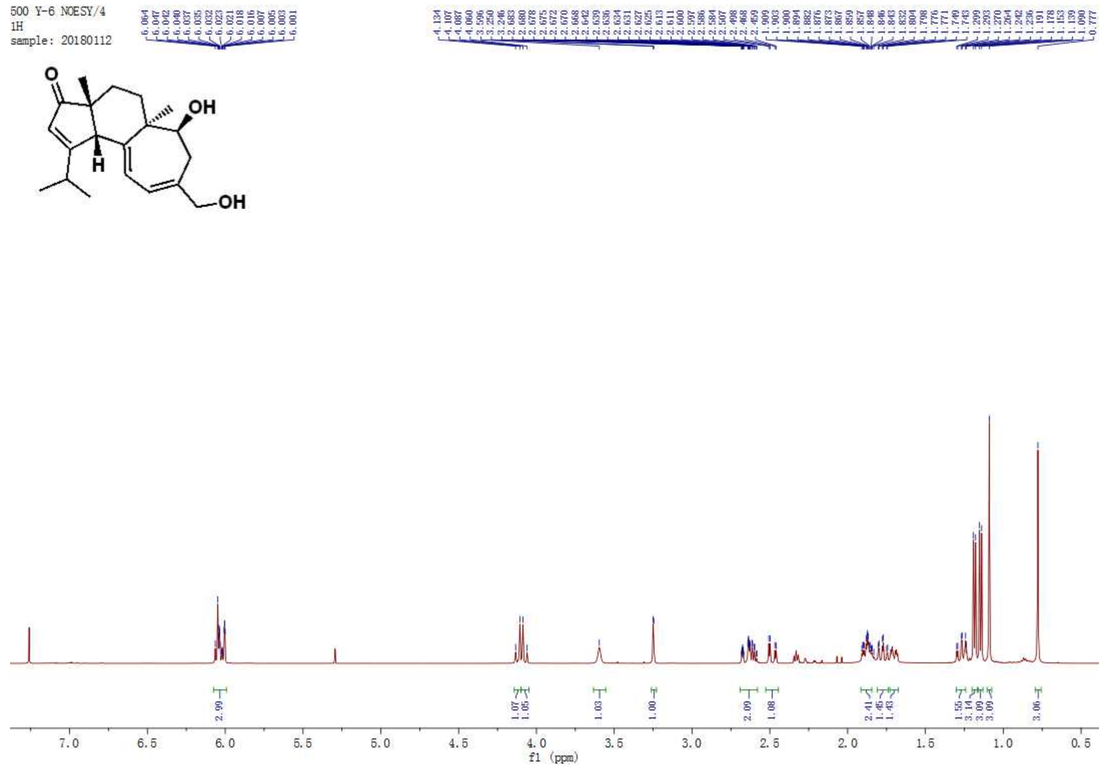

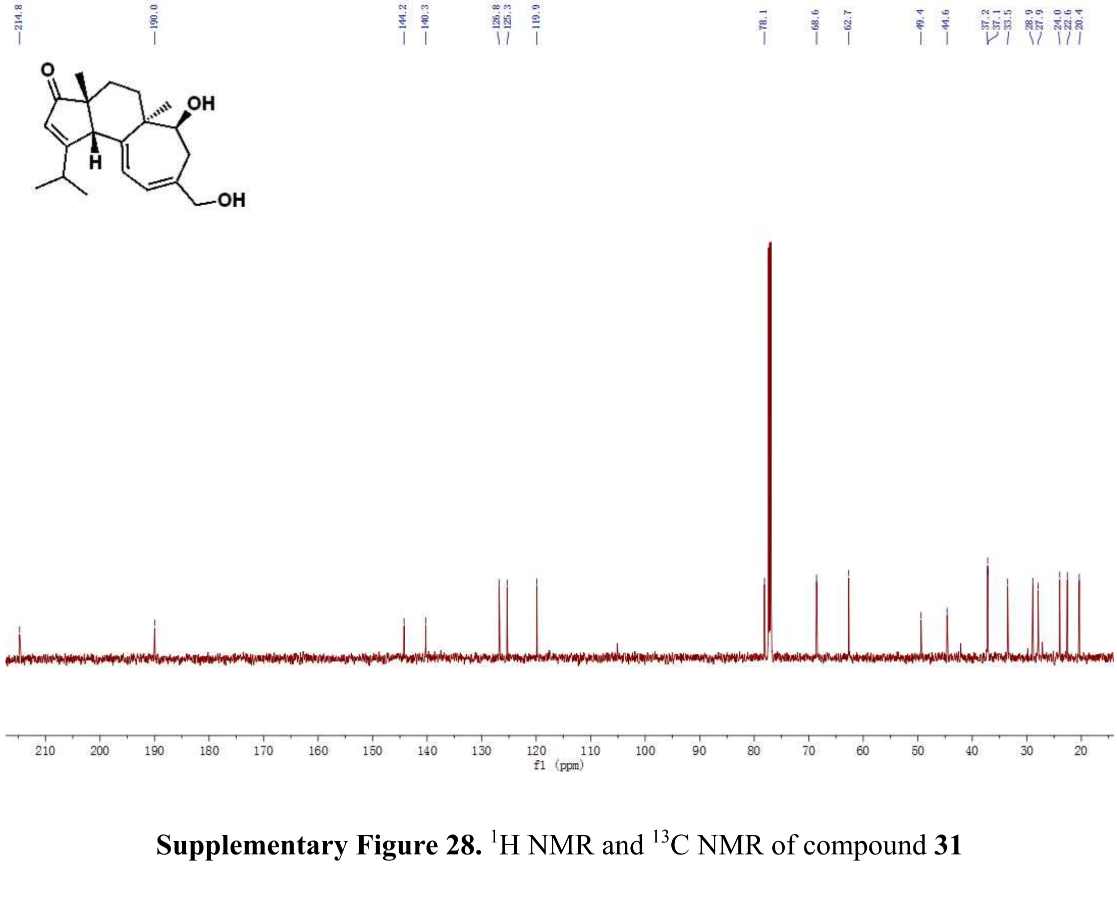

Supplementary Figure 28. ^{1}H NMR and ^{13}C NMR of compound **31**

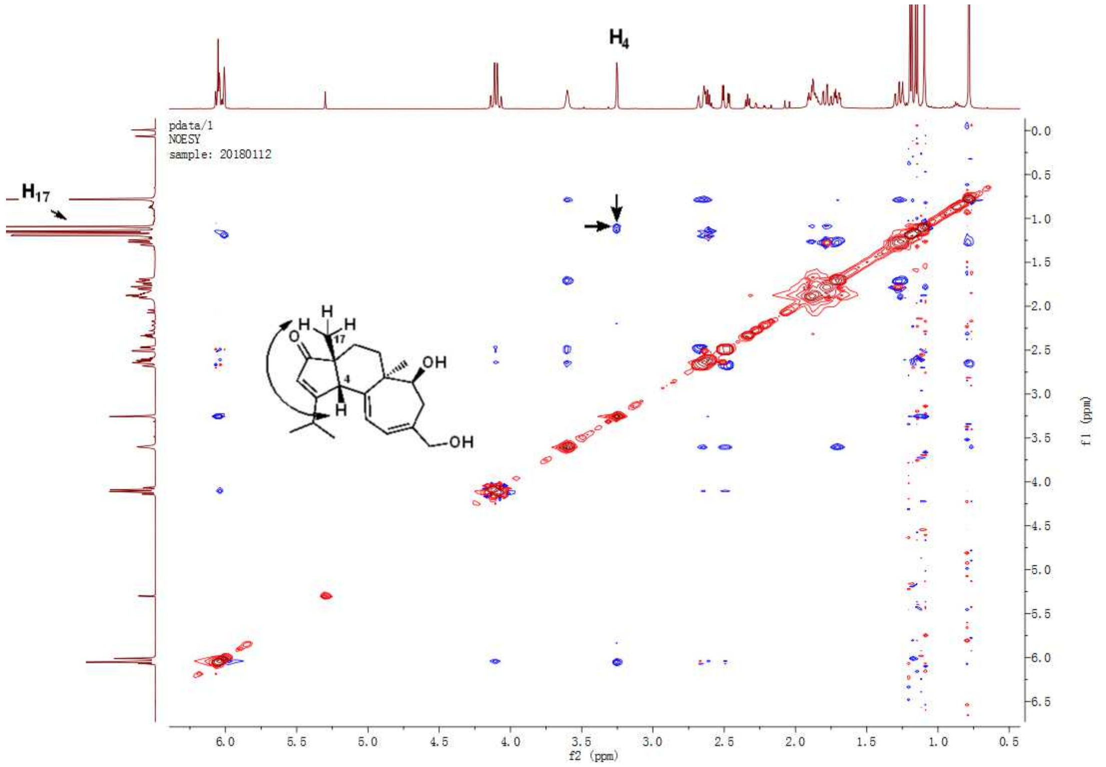

Supplementary Figure 29. Noesy correlation of compound **31**

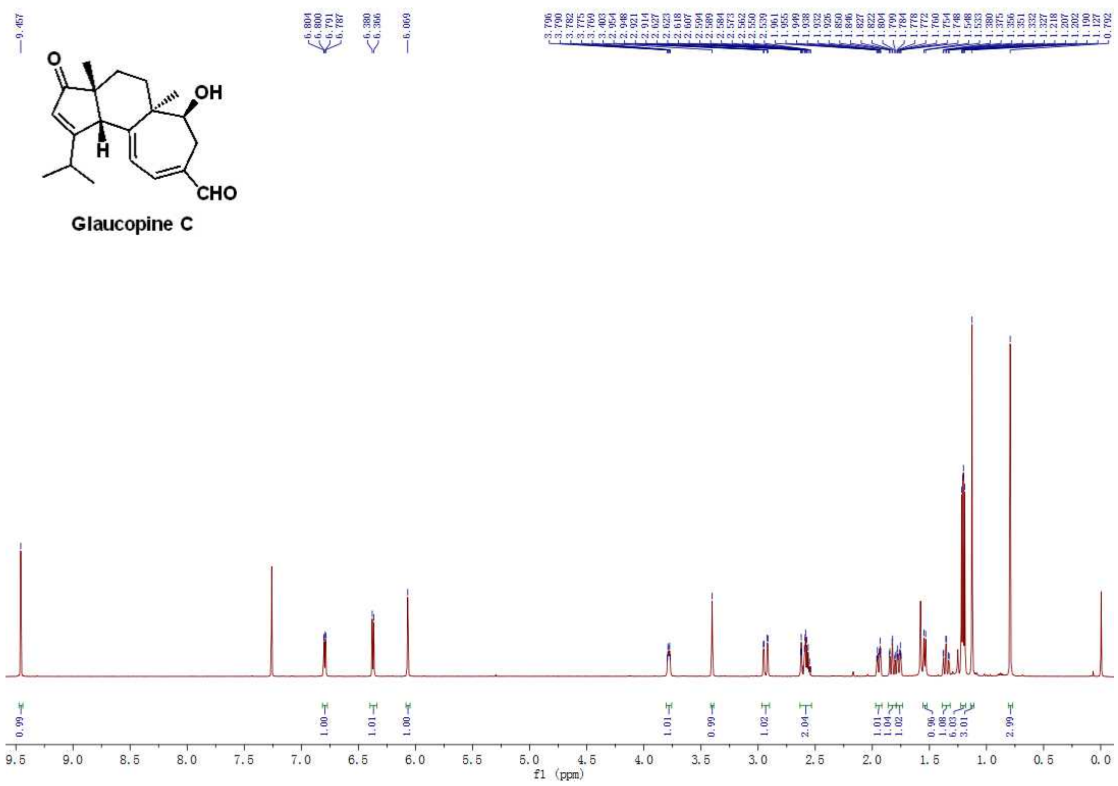

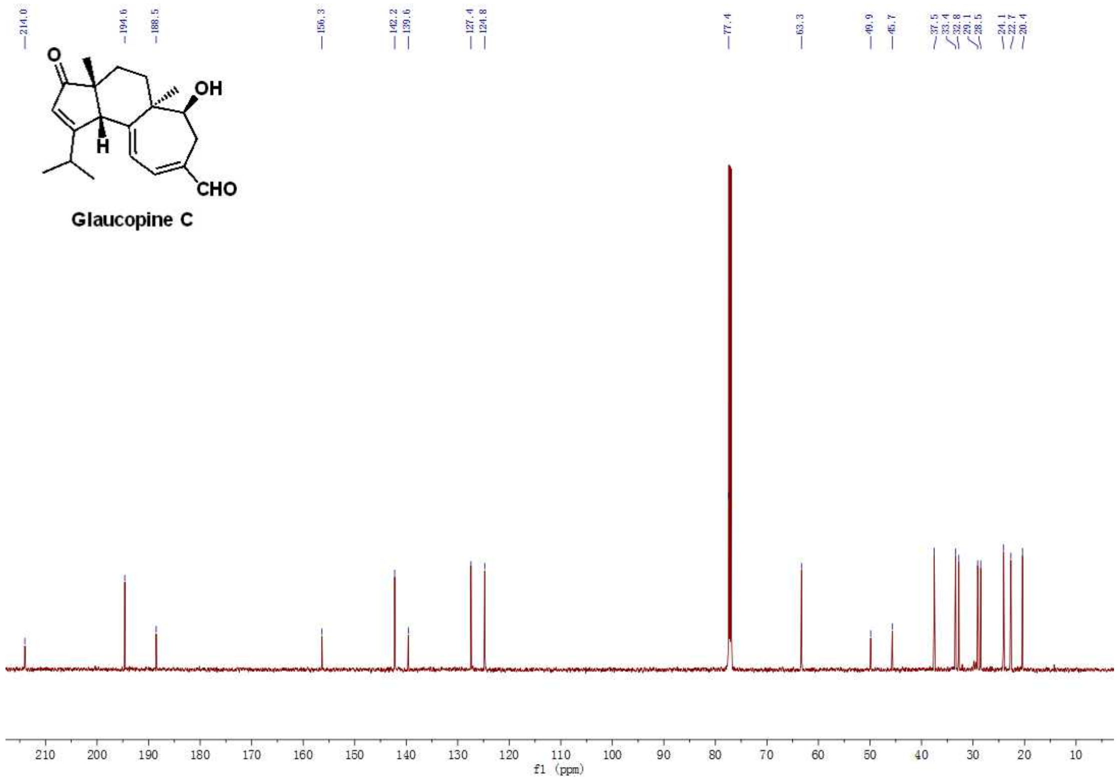

Supplementary Figure 30. ^{1}H NMR and ^{13}C NMR of Glaucopine C

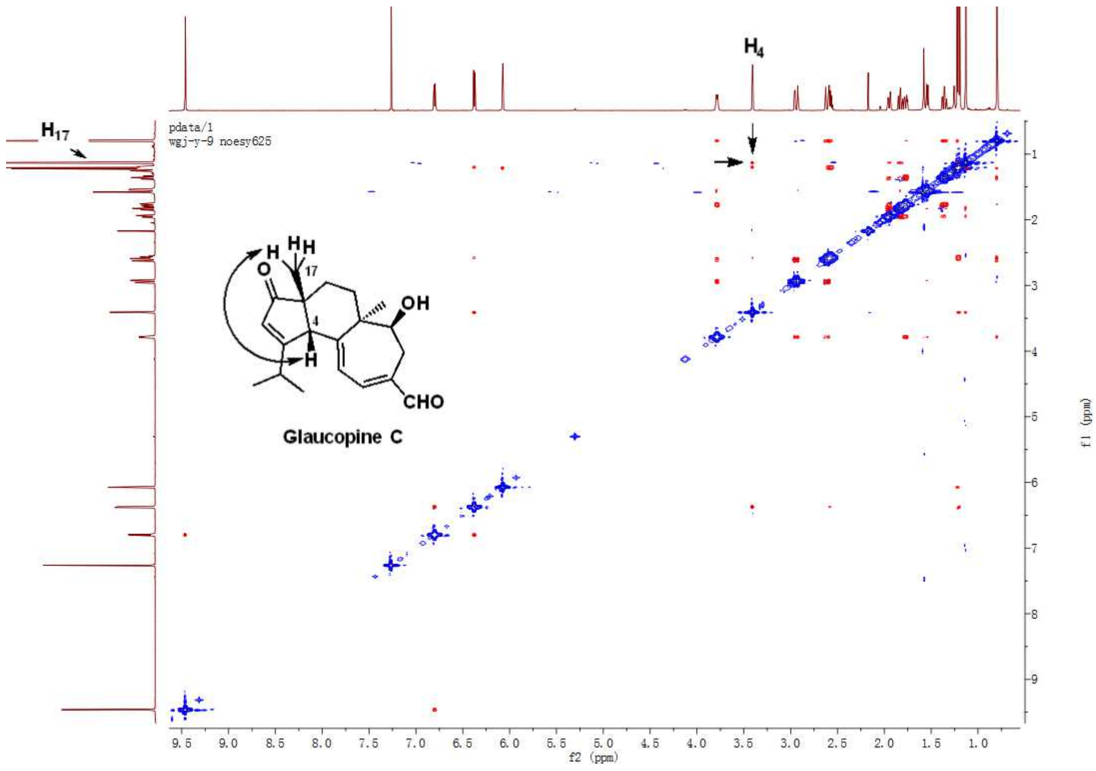

Supplementary Figure 31. Noesy correlation of Glaucopine C

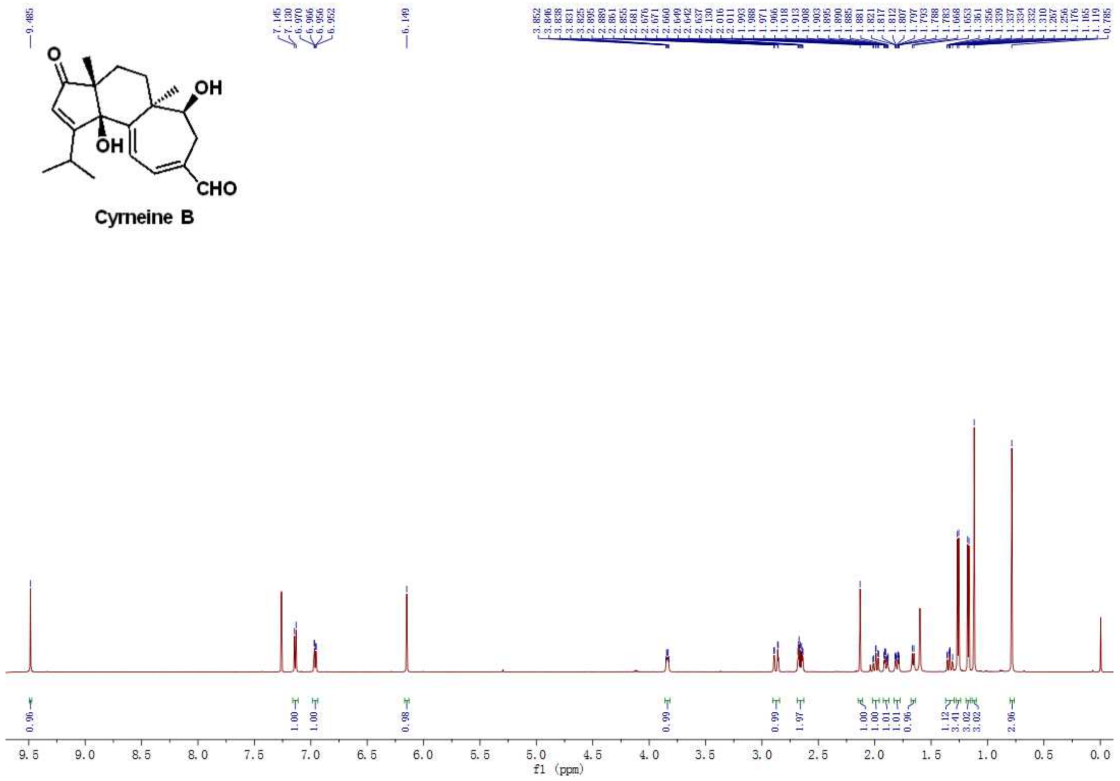

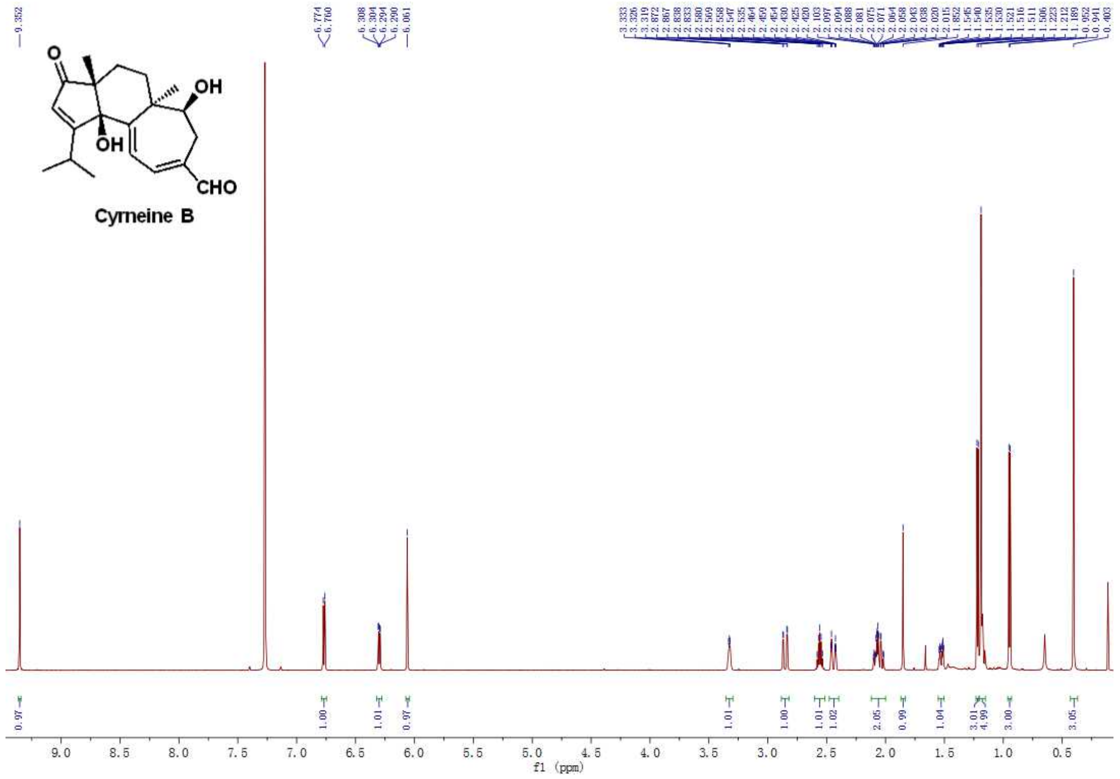

Supplementary Figure 32. ^{1}H NMR of Cyrneine B in $CDCl_3$ (upper) and C_6D_6 (bottom)

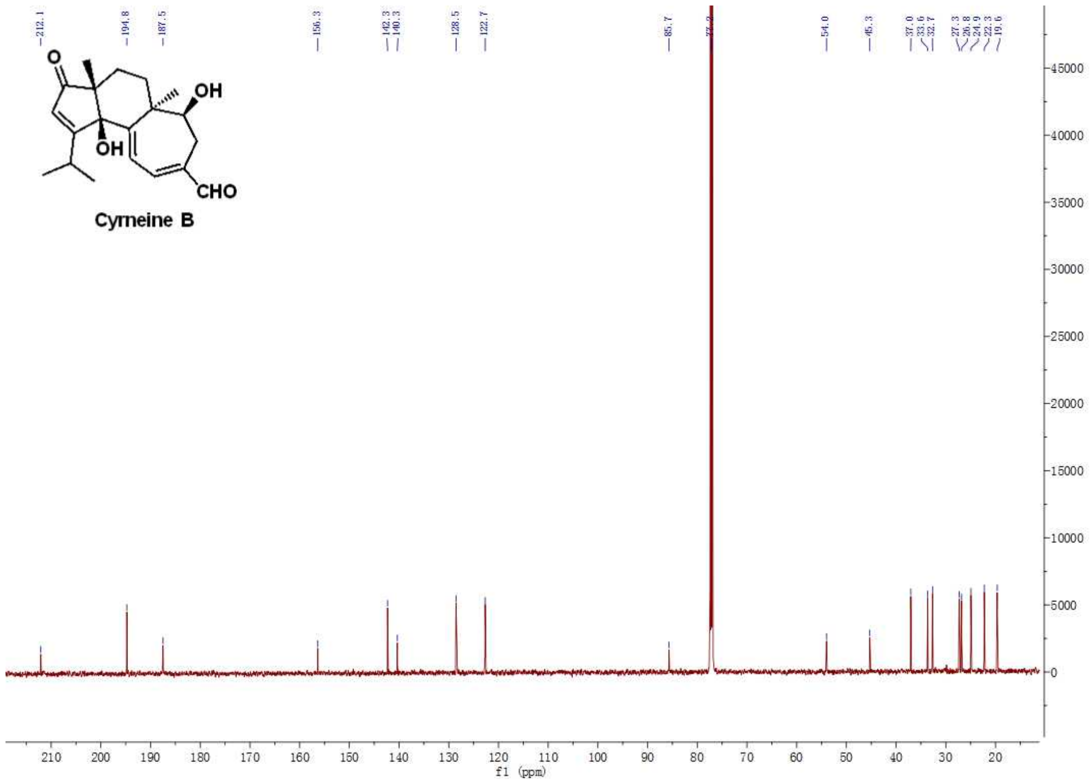

Supplementary Figure 33. ^{13}C NMR of Cyrneine B in $CDCl_3$

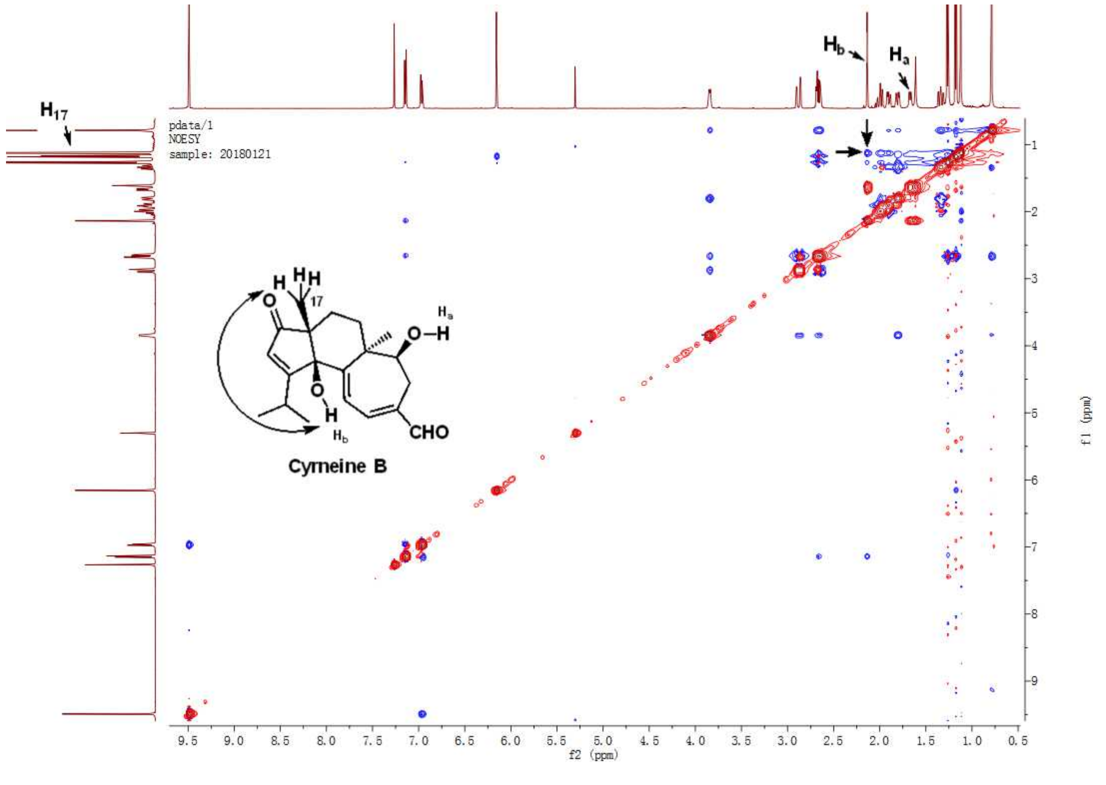

Supplementary Figure 34. Noesy correlation of Cyrneine B in $CDCl_3$

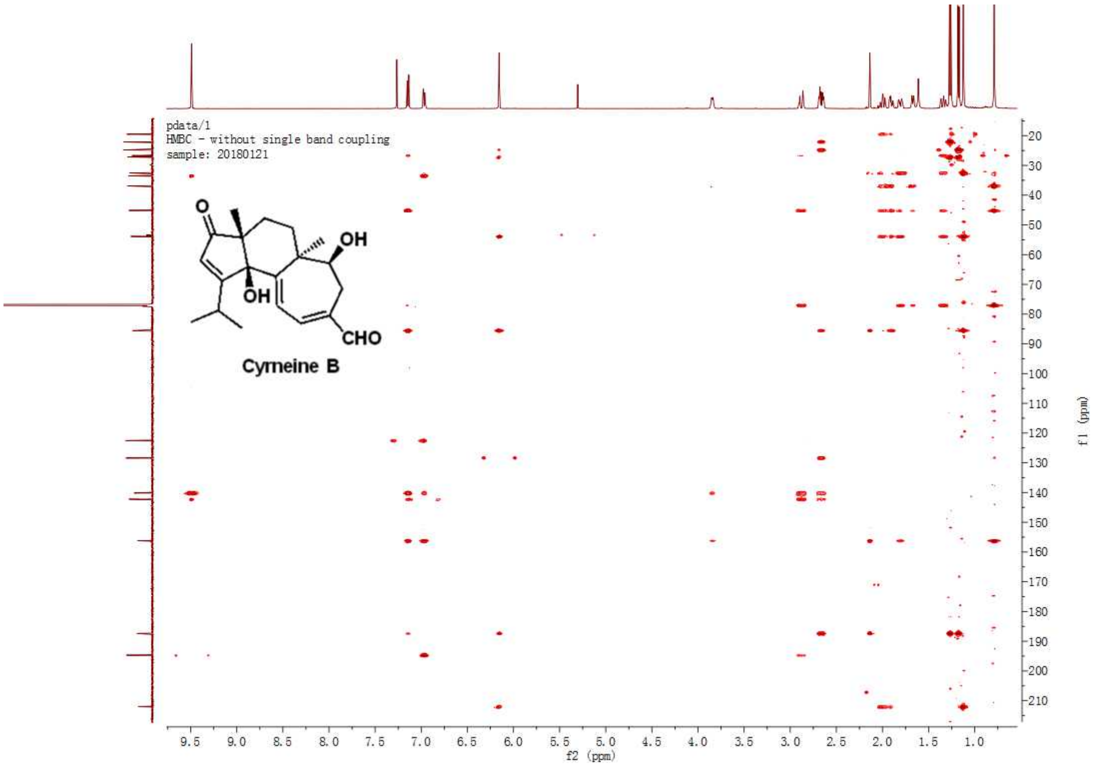

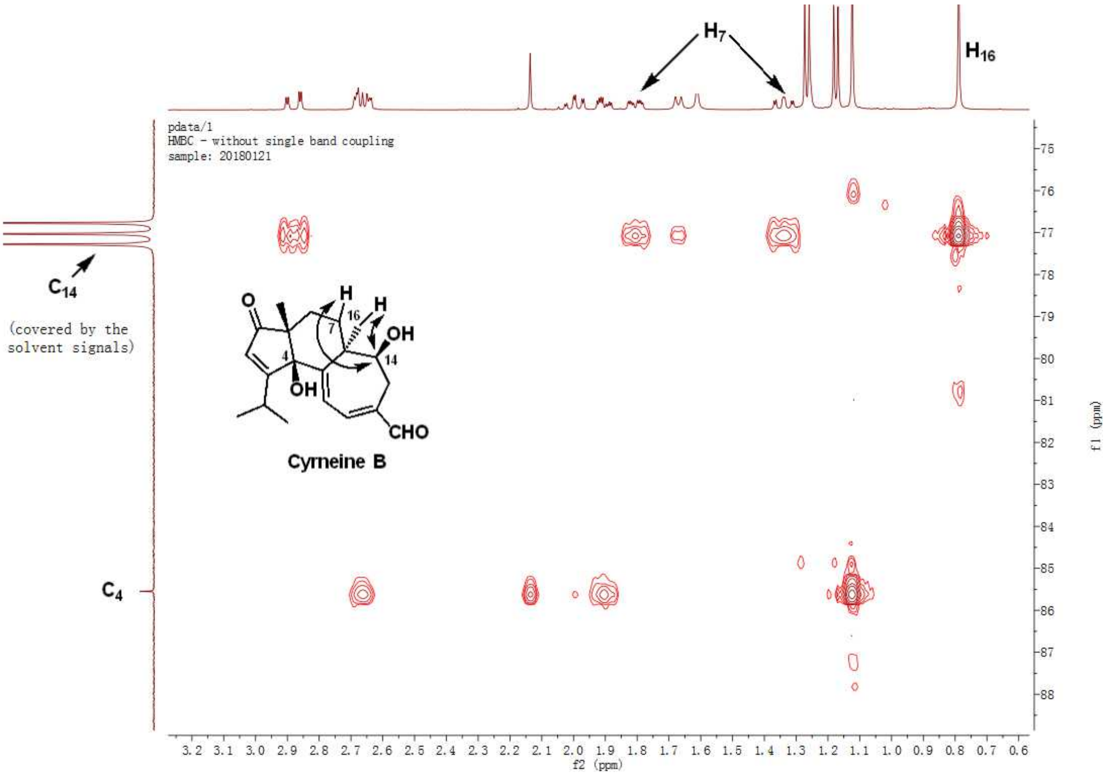

Supplementary Figure 35. HMBC correlation of Cyrneine B in $CDCl_3$: full spectrum (upper) and zoomed (bottom)

5. X-Ray Single Crystal Diffraction Data for Cyrneine A (1)

Supplementary Table 7. Crystal data and structure refinement for **1**.

	(CCDC 1830226)
Formula	C20 H28 O3
Formula weight	316.42
Temperature	150(2) K
Z, calculated density	4, 1.217 Mg·m^{-3}
F(000)	688
absorption coefficient	0.632 mm^{-1}
radiation(wavelength)	Cu K_{α} (1.54178 Å)
Crystal system, space group	orthorhombic, P 21 21 21
Unit cell dimensions	a = 6.9296(2) Å alpha = 90°
	b = 13.6851(3) Å beta = 90°
	c = 18.2162(4) Å gamma = 90°
Volume	1727.48(7) Å^3
Crystal size	0.400 x 0.300 x 0.250 mm
Theta range for data collection	4.855° to 72.395°
Limiting indices	-8<=h<=8, -15<=k<=16, -22<=l<=22
Reflections collected/unique	22615/3397 [R(int) = 0.0453]
Completeness to theta = 72.679	0.995
Absorption correction	None
Refinement method	Full-matrix least-squares on F^2
Data/restraints/parameters	3397/0/214
Goodness-of-fit on F^2	1.068
Final R indices [I>2sigma(I)]	R1 = 0.0353, wR2 = 0.0921
R indices (all data)	R1 = 0.0366, wR2 = 0.0936
Absolute structure parameter	-0.02(7)
Extinction coefficient	n/a
Reflections collected	22615
number of refined parameters	214
Largest diff. peak and hole	0.236 and -0.216 e.A^{-3}

Supplementary Table 8. Atomic coordinates (x 10^4) and equivalent isotropic displacement parameters (A^2 x 10^3) for **1**. U(eq) is defined as one third of the trace of the orthogonalized Uij tensor.

	x	y	z	U(eq)
O(2)	8631(2)	5952(1)	5766(1)	25(1)
O(1)	6352(2)	4549(1)	9255(1)	26(1)
O(3)	9749(2)	8909(1)	4681(1)	29(1)
C(4)	9991(3)	5294(1)	7165(1)	21(1)
C(1)	7441(3)	6528(1)	8012(1)	16(1)
C(10)	6998(2)	7561(1)	6941(1)	17(1)
C(20)	8656(3)	8841(1)	5201(1)	21(1)
C(16)	11652(3)	6911(2)	7350(1)	25(1)
C(13)	10503(3)	7457(2)	5796(1)	24(1)
C(7)	6722(3)	7047(1)	8577(1)	19(1)
C(15)	5889(3)	4911(2)	7787(1)	23(1)
C(6)	7536(3)	5439(1)	8186(1)	18(1)
C(3)	9921(3)	6403(1)	6981(1)	16(1)
C(12)	8798(3)	8124(1)	5796(1)	18(1)
C(11)	7321(3)	8127(1)	6278(1)	17(1)
C(5)	9507(3)	5039(1)	7958(1)	21(1)
C(8)	6111(3)	6361(1)	9190(1)	22(1)
C(17)	6531(3)	8137(1)	8675(1)	23(1)
C(2)	8077(3)	6868(1)	7278(1)	15(1)
C(9)	7250(3)	5432(1)	9025(1)	20(1)
C(14)	10157(3)	6456(1)	6139(1)	20(1)
C(18)	4476(4)	8429(2)	8893(1)	35(1)
C(19)	7965(4)	8494(2)	9254(1)	39(1)

Supplementary Table 9. Bond lengths [Å] and angles [deg] for **1**.

O(2)-C(14)	1.434(2)
O(2)-H(2)	0.8400
O(1)-C(9)	1.422(2)
O(1)-H(1)	0.8400
O(3)-C(20)	1.216(2)
C(4)-C(5)	1.522(3)
C(4)-C(3)	1.556(2)
C(4)-H(4A)	0.9900
C(4)-H(4B)	0.9900
C(1)-C(7)	1.346(3)
C(1)-C(2)	1.483(2)

C(1)-C(6)	1.525(2)
C(10)-C(2)	1.354(3)
C(10)-C(11)	1.453(2)
C(10)-H(10)	0.9500
C(20)-C(12)	1.465(3)
C(20)-H(20)	0.9500
C(16)-C(3)	1.540(3)
C(16)-H(16A)	0.9800
C(16)-H(16B)	0.9800
C(16)-H(16C)	0.9800
C(13)-C(12)	1.493(3)
C(13)-C(14)	1.524(3)
C(13)-H(13A)	0.9900
C(13)-H(13B)	0.9900
C(7)-C(17)	1.509(3)
C(7)-C(8)	1.518(3)
C(15)-C(6)	1.534(3)
C(15)-H(15A)	0.9800
C(15)-H(15B)	0.9800
C(15)-H(15C)	0.9800
C(6)-C(5)	1.529(3)
C(6)-C(9)	1.542(2)
C(3)-C(2)	1.526(2)
C(3)-C(14)	1.544(2)
C(12)-C(11)	1.349(3)
C(11)-H(11)	0.9500
C(5)-H(5A)	0.9900
C(5)-H(5B)	0.9900
C(8)-C(9)	1.527(3)
C(8)-H(8A)	0.9900
C(8)-H(8B)	0.9900
C(17)-C(19)	1.529(3)
C(17)-C(18)	1.531(3)
C(17)-H(17)	1.0000
C(9)-H(9)	1.0000
C(14)-H(14)	1.0000
C(18)-H(18A)	0.9800
C(18)-H(18B)	0.9800
C(18)-H(18C)	0.9800
C(19)-H(19A)	0.9800
C(19)-H(19B)	0.9800
C(19)-H(19C)	0.9800
C(14)-O(2)-H(2)	109.5
C(9)-O(1)-H(1)	109.5

C(5)-C(4)-C(3)	114.93(15)
C(5)-C(4)-H(4A)	108.5
C(3)-C(4)-H(4A)	108.5
C(5)-C(4)-H(4B)	108.5
C(3)-C(4)-H(4B)	108.5
H(4A)-C(4)-H(4B)	107.5
C(7)-C(1)-C(2)	129.40(16)
C(7)-C(1)-C(6)	111.85(16)
C(2)-C(1)-C(6)	118.73(15)
C(2)-C(10)-C(11)	131.74(17)
C(2)-C(10)-H(10)	114.1
C(11)-C(10)-H(10)	114.1
O(3)-C(20)-C(12)	125.80(19)
O(3)-C(20)-H(20)	117.1
C(12)-C(20)-H(20)	117.1
C(3)-C(16)-H(16A)	109.5
C(3)-C(16)-H(16B)	109.5
H(16A)-C(16)-H(16B)	109.5
C(3)-C(16)-H(16C)	109.5
H(16A)-C(16)-H(16C)	109.5
H(16B)-C(16)-H(16C)	109.5
C(12)-C(13)-C(14)	115.18(16)
C(12)-C(13)-H(13A)	108.5
C(14)-C(13)-H(13A)	108.5
C(12)-C(13)-H(13B)	108.5
C(14)-C(13)-H(13B)	108.5
H(13A)-C(13)-H(13B)	107.5
C(1)-C(7)-C(17)	130.13(17)
C(1)-C(7)-C(8)	109.87(16)
C(17)-C(7)-C(8)	119.96(16)
C(6)-C(15)-H(15A)	109.5
C(6)-C(15)-H(15B)	109.5
H(15A)-C(15)-H(15B)	109.5
C(6)-C(15)-H(15C)	109.5
H(15A)-C(15)-H(15C)	109.5
H(15B)-C(15)-H(15C)	109.5
C(1)-C(6)-C(5)	109.42(15)
C(1)-C(6)-C(15)	109.28(15)
C(5)-C(6)-C(15)	111.51(16)
C(1)-C(6)-C(9)	101.95(14)
C(5)-C(6)-C(9)	112.44(15)
C(15)-C(6)-C(9)	111.78(15)
C(2)-C(3)-C(16)	108.07(15)
C(2)-C(3)-C(14)	114.88(15)

C(16)-C(3)-C(14)	109.25(15)
C(2)-C(3)-C(4)	110.86(15)
C(16)-C(3)-C(4)	108.76(15)
C(14)-C(3)-C(4)	104.87(14)
C(11)-C(12)-C(20)	115.32(17)
C(11)-C(12)-C(13)	127.04(16)
C(20)-C(12)-C(13)	117.62(16)
C(12)-C(11)-C(10)	131.00(17)
C(12)-C(11)-H(11)	114.5
C(10)-C(11)-H(11)	114.5
C(4)-C(5)-C(6)	111.84(15)
C(4)-C(5)-H(5A)	109.2
C(6)-C(5)-H(5A)	109.2
C(4)-C(5)-H(5B)	109.2
C(6)-C(5)-H(5B)	109.2
H(5A)-C(5)-H(5B)	107.9
C(7)-C(8)-C(9)	103.10(15)
C(7)-C(8)-H(8A)	111.1
C(9)-C(8)-H(8A)	111.1
C(7)-C(8)-H(8B)	111.1
C(9)-C(8)-H(8B)	111.1
H(8A)-C(8)-H(8B)	109.1
C(7)-C(17)-C(19)	109.92(17)
C(7)-C(17)-C(18)	111.70(18)
C(19)-C(17)-C(18)	110.05(18)
C(7)-C(17)-H(17)	108.4
C(19)-C(17)-H(17)	108.4
C(18)-C(17)-H(17)	108.4
C(10)-C(2)-C(1)	117.66(16)
C(10)-C(2)-C(3)	126.38(16)
C(1)-C(2)-C(3)	115.96(15)
O(1)-C(9)-C(8)	115.05(16)
O(1)-C(9)-C(6)	110.75(14)
C(8)-C(9)-C(6)	104.77(15)
O(1)-C(9)-H(9)	108.7
C(8)-C(9)-H(9)	108.7
C(6)-C(9)-H(9)	108.7
O(2)-C(14)-C(13)	110.70(16)
O(2)-C(14)-C(3)	111.76(15)
C(13)-C(14)-C(3)	117.78(16)
O(2)-C(14)-H(14)	105.1
C(13)-C(14)-H(14)	105.1
C(3)-C(14)-H(14)	105.1
C(17)-C(18)-H(18A)	109.5

C(17)-C(18)-H(18B)	109.5
H(18A)-C(18)-H(18B)	109.5
C(17)-C(18)-H(18C)	109.5
H(18A)-C(18)-H(18C)	109.5
H(18B)-C(18)-H(18C)	109.5
C(17)-C(19)-H(19A)	109.5
C(17)-C(19)-H(19B)	109.5
H(19A)-C(19)-H(19B)	109.5
C(17)-C(19)-H(19C)	109.5
H(19A)-C(19)-H(19C)	109.5
H(19B)-C(19)-H(19C)	109.5

Supplementary Table 10. Anisotropic displacement parameters (A^2 x 10^3) for **1**.
The anisotropic displacement factor exponent takes the form:
-2 pi^2 [h^2 a*^2 U11 + ... + 2 h k a* b* U12]

	U11	U22	U33	U23	U13	U12
O(2)	32(1)	23(1)	18(1)	-6(1)	-4(1)	6(1)
O(1)	40(1)	22(1)	16(1)	7(1)	-2(1)	-8(1)
O(3)	28(1)	36(1)	22(1)	9(1)	4(1)	-1(1)
C(4)	24(1)	16(1)	22(1)	0(1)	2(1)	4(1)
C(1)	17(1)	16(1)	16(1)	2(1)	-2(1)	-1(1)
C(10)	18(1)	18(1)	15(1)	-2(1)	1(1)	0(1)
C(20)	24(1)	20(1)	18(1)	2(1)	-2(1)	-1(1)
C(16)	20(1)	26(1)	30(1)	1(1)	-4(1)	-3(1)
C(13)	24(1)	26(1)	22(1)	8(1)	8(1)	5(1)
C(7)	23(1)	19(1)	15(1)	2(1)	-1(1)	-1(1)
C(15)	28(1)	21(1)	19(1)	1(1)	-1(1)	-6(1)
C(6)	22(1)	15(1)	16(1)	2(1)	-1(1)	-2(1)
C(3)	16(1)	16(1)	18(1)	1(1)	-1(1)	1(1)
C(12)	22(1)	17(1)	16(1)	0(1)	-2(1)	-1(1)
C(11)	20(1)	14(1)	15(1)	-1(1)	-4(1)	2(1)
C(5)	26(1)	17(1)	21(1)	4(1)	-1(1)	3(1)
C(8)	29(1)	23(1)	15(1)	2(1)	4(1)	-2(1)
C(17)	37(1)	17(1)	15(1)	-2(1)	1(1)	-1(1)
C(2)	18(1)	14(1)	13(1)	-2(1)	-1(1)	-2(1)
C(9)	25(1)	19(1)	15(1)	3(1)	-2(1)	-5(1)
C(14)	21(1)	21(1)	19(1)	0(1)	4(1)	5(1)
C(18)	47(1)	28(1)	32(1)	2(1)	9(1)	10(1)
C(19)	59(2)	26(1)	31(1)	-1(1)	-10(1)	-9(1)

Supplementary Table 11. Hydrogen coordinates (x 10^4) and isotropic displacement parameters (A^2 x 10^3) for **1**.

	x	y	z	U(eq)
H(2)	7600	6270	5809	37
H(1)	6429	4500	9714	39
H(4A)	9075	4948	6839	25
H(4B)	11301	5047	7054	25
H(10)	5815	7702	7182	20
H(20)	7617	9293	5224	25
H(16A)	11635	6775	7878	38
H(16B)	11568	7617	7270	38
H(16C)	12855	6662	7137	38
H(13A)	11569	7780	6064	29
H(13B)	10931	7362	5283	29
H(15A)	4649	5125	7990	34
H(15B)	6027	4204	7854	34
H(15C)	5939	5067	7262	34
H(11)	6321	8578	6164	20
H(5A)	10512	5311	8285	26
H(5B)	9511	4320	8016	26
H(8A)	4704	6237	9175	27
H(8B)	6457	6631	9676	27
H(17)	6849	8461	8198	28
H(9)	8543	5481	9267	24
H(14)	11349	6071	6030	24
H(18A)	3573	8222	8509	53
H(18B)	4405	9140	8952	53
H(18C)	4133	8111	9357	53
H(19A)	7653	8198	9729	58
H(19B)	7886	9207	9294	58
H(19C)	9276	8306	9109	58

6. HPLC Charts for compound 12

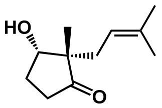

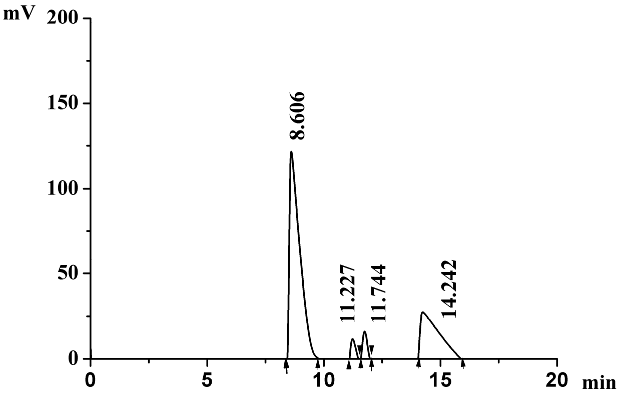

Pk #	Retention Time	Area	Area %	Height	Height %
1	8.606	4323765	46.012	125152	57.245
2*	11.227	473076	5.034	22592	10.334
3*	11.744	685244	7.292	27958	12.788
4	14.242	3915053	41.662	42922	19.633
Totals		9397138	100.000	218622	100.000

* The diastereoisomer of racemic compound **12**

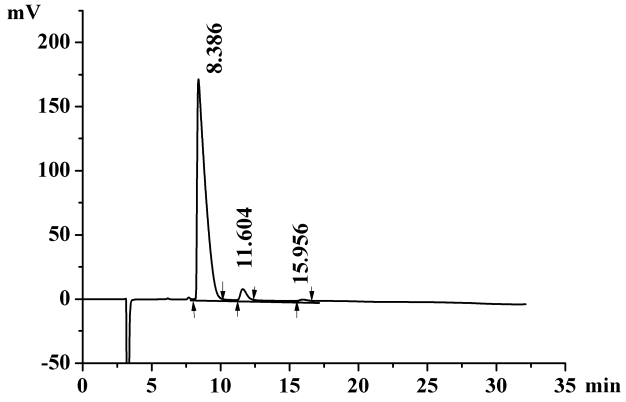

Pk #	Retention Time	Area	Area %	Height	Height %
1	8.386	6952364	95.649	171132	94.738
2*	11.604	278975	3.838	8463	4.685
3	15.956	37266	0.513	1042	0.577
Totals		7268605	100.000	180637	100.000

* The diastereoisomer of chiral **12**.

Supplementary Figure 36. HPLC charts of racemic (upper) and chiral (bottom) compound **12**

Part II. Cross-coupling of Enol Triflates

7. Experimental Procedures and Characterization Data

7.1 Synthesis of Starting materials

7.1.1 Synthesis of 15b.

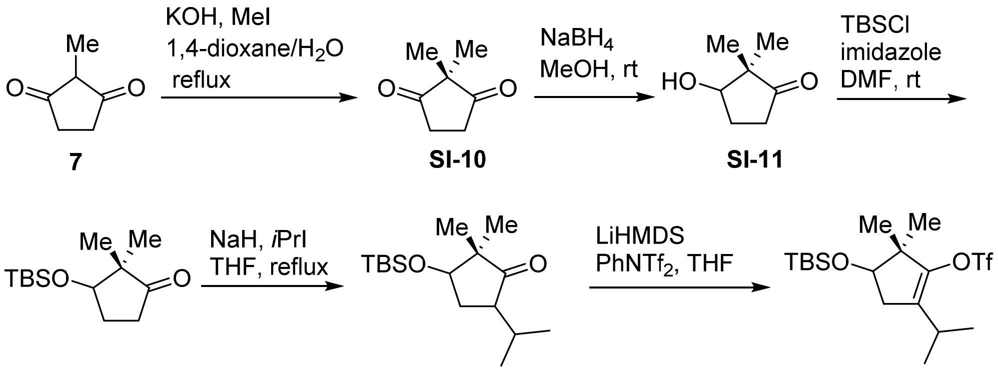

Supplementary Figure 37. Synthesis of compound **15b**

Hydroxyketone **SI-11** was prepared according to the published procedure.[4,5] The synthesis of **15b** from **SI-11** was as below:

To a solution of compound **SI-11** (2.82 g, 22.0 mmol) in DMF (20 ml) was added imidazole (3.00 g, 44.0 mmol, 2.0 equiv) and TBSCl (6.63 g, 44.0 mmol, 2.0 equiv). The mixture was stirred overnight at room temperature. The reaction mixture was poured into sodium bicarbonate solution (50 mL) and extracted with ethyl acetate (3 × 100 mL). The combined organic layers were washed with brine (5 × 20 mL), dried over Na_2SO_4 and evaporated to dryness. The resulting crude residue was purified by flash column chromatography (eluent: petroleum ether/EtOAc = 30/1) to afford **SI-12** (4.8 g, 90%) as a colorless oil. R_f = 0.3 (petroleum ether/EtOAc = 15/1).

SI-12 (5.09 g, 21.0 mmol) in THF (20 mL) was added dropwise to a suspension of NaH (60% dispersion in mineral oil, 4.2 g, 105 mmol, 5.0 equiv) in THF (140 mL) at 0 °C, then *i*PrI (21.0 mL, 210.0 mmol, 10.0 equiv) was added. The reaction mixture was refluxed overnight. The resulting mixture was quenched at 0 °C by adding H_2O. Then 2 M HCl (60 mL) was added, and the mixture was stirred at room temperature for 1 h. Then brine was added to the reaction mixture, and the organic layer was separated. The aqueous layer was extracted with ethyl acetate (3 × 60 mL). The combined organic layers were washed with brine (50 mL), dried over Na_2SO_4 and concentrated under reduced pressure. The resulting crude residue was purified by flash column chromatography (eluent:petroleum ether/CH_2Cl_2 = 5/1) to afford **SI-13** (3.48 g, 58%) as a colorless oil. R_f = 0.3 (petroleum ether/CH_2Cl_2 = 3/2).

Ketone **SI-13** (1.42 g, 5.0 mmol) was dissolved in dry THF (8 mL) and the mixture was cooled to -78 °C. LiHMDS (1.0 M in THF, 10.0 mL, 10.0 mmol, 2.0 equiv) was added and the clear solution was stirred at -40 °C for 1 h. Then $PhNTf_2$ (3.57 g, 10.0 mmol, 2.0 equiv) dissolved in dry THF (15 mL) was slowly added at -78 °C and the mixture was allowed to warm to room temperature and stirred for additional 4 h. Then brine (50 mL) was added to the reaction mixture, and the organic layer was separated. The aqueous layer was extracted with ethyl acetate (3 × 50 mL). The combined organic layers were dried over Na_2SO_4 and concentrated under reduced pressure. The residue was purified by flash chromatography (eluent: petroleum) to afford **15b** (1.64 g, 79%) as a colorless oil. R_f = 0.4 (petroleum ether/CH_2Cl_2 = 50/1); ^{1}H NMR (300 MHz, $CDCl_3$) δ = 3.91 (t, J = 7.2 Hz, 1H), 2.79 (sept, J = 6.6 Hz, 1H), 2.44 (dd, J = 15.0, 7.2 Hz, 1H), 2.11 (dd, J = 15.0, 7.2 Hz, 1H), 1.06 (s, 3H), 1.01 (d, J = 6.6 Hz, 3H), 1.00 (s, 3H), 0.99 (d, J = 6.6 Hz, 3H), 0.90 (s, 9H), 0.07 (s, 3H), 0.07 (s, 3H); ^{13}C NMR (101 MHz, $CDCl_3$) δ 145.0, 134.1, 118.8 (d, J = 321 Hz), 77.5, 46.5, 34.0, 25.9, 25.5, 23.8, 20.4, 20.2, 18.6, 18.2, -4.3, -4.7.

7.1.2 Synthesis of 15c.

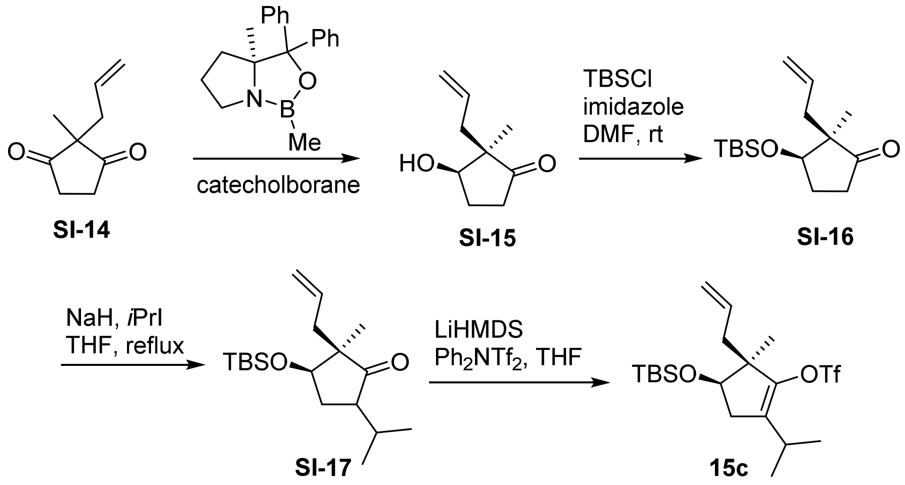

Supplementary Figure 38. Synthesis of compound **15b**

SI-16 was prepared according to the published procedure.[6] The synthesis of **15c** from **SI-16** was as following:

SI-16 (2.55 g, 9.5 mmol) in THF (30 mL) was added dropwise to a suspension of NaH (60% dispersion in mineral oil, 1.9 g, 47.5 mmol, 5.0 equiv) in THF (50 mL) at 0 °C, then *i*PrI (9.5 mL, 95.0 mmol, 10.0 equiv) was added. The reaction mixture was refluxed overnight. The resulting mixture was quenched at 0 °C by adding H_2O. Then 2 M HCl (35 mL) was added, and the mixture stirred at room temperature for 1 h. Then brine was added to the reaction mixture. The organic layer was separated and the aqueous layer was extracted with ethyl

acetate (3 × 50 mL). The combined organic layers were washed with brine (50 mL), dried over Na_2SO_4 and concentrated under reduced pressure. The resulting crude residue was purified by flash column chromatography (eluent: petroleum ether/CH_2Cl_2 = 5/1) to afford **SI-17** (1.74 g, 59%) as a colorless oil. R_f = 0.3 (petroleum ether/CH_2Cl_2 = 2/1).

Ketone **SI-17** (1.74 g, 5.6 mmol) was dissolved in dry THF (10 mL) and the solution was cooled to -78 °C. LiHMDS (1.0 M in THF, 11.2 mL, 11.2 mmol, 2.0 equiv) was added and the clear solution was stirred at -40 °C for 1 h. Then $PhNTf_2$ (4.0 g, 2.0 equiv) in dry THF (15 mL) was slowly added at -78°C and the mixture was allowed to warm to room temperature and stirred for additional 4 h. Then brine (50 mL) was added to the reaction mixture. The organic layer was separated and the aqueous layer was extracted with ethyl acetate (3 × 50 mL). The combined organic layers were dried over Na_2SO_4 and concentrated under reduced pressure. The residue was purified by flash chromatography (eluent: petroleum ether) to afford **15c** (2.0 g, 81% yield) as a colorless oil. R_f = 0.4 (petroleum ether/CH_2Cl_2 = 50/1); ^1H NMR (600 MHz, $CDCl_3$) δ = 5.91–5.82 (m, 1H), 5.04–4.96 (m, 2H), 4.03 (t, J = 7.8 Hz, 1H), 2.83 (sept, J = 6.9 Hz, 1H), 2.42 (dd, J = 15.0, 7.8 Hz, 1H), 2.28 (dd, J = 13.8, 7.2 Hz, 1H), 2.15–2.06 (m, 2H), 1.06 (s, 3H), 1.01 (d, J = 6.9 Hz, 3H), 0.97 (d, J = 6.9 Hz, 3H), 0.92 (s, 9H), 0.09 (s, 3H), 0.08 (s, 3H).

7.1.3 Synthesis of 15d.

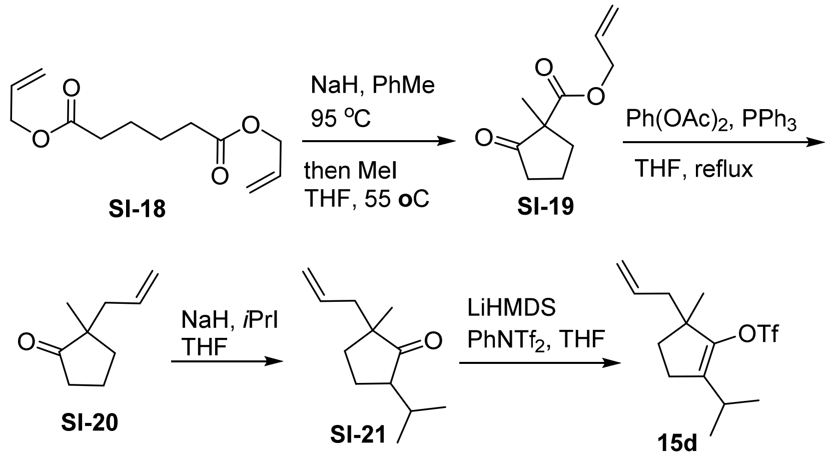

Supplementary Figure 39. Synthesis of compound **15d**

SI-20 was prepared from **SI-18** in 2 steps by a modification of reported procedure.[7,8]

To a stirred suspension of NaH (60% dispersion in mineral oil, 3.08 g, 77.0 mmol, 2.2 equiv) in dry toluene (50 mL) was added dropwise a solution of diallyl adipate (**SI-18**, 7.76 mL, 35 mmol) in toluene (50 mL) at room temperature. The resulting mixture was heated at 95 °C for 2 h. The solvent was removed under reduced pressure and then THF (50 mL) and

MeI (2.83 mL, 45.5 mmol) was added successively at room temperature. The mixture was heated at 55 ºC for 4 h. Then brine (50 mL) was added to the reaction mixture, and the organic layer was separated. The aqueous layer was extracted with ethyl acetate (3 × 30 mL). The combined organic layers were dried over Na_2SO_4 and concentrated under reduced pressure. The residue was purified by flash chromatography (eluent:petroleum ether/EtOAc = 50/1→20/1) to afford **SI-19**[9] (4.82 g, 76%) as a colorless oil. R_f= 0.3 (petroleum ether/EtOAc = 10/1). ^{1}H NMR (300 MHz, $CDCl_3$) δ = 5.98–5.79 (m, 1H), 5.37–5.16 (m, 2H), 4.60 (d, *J* = 5.7 Hz, 2H), 2.61–2.25 (m, 3H), 2.15–21.80 (m, 3H), 1.33 (s, 3H).

SI-20 was prepared from **SI-19** according to the published procedure.[8] The synthesis of **15d** from **SI-20** was as following:

To a stirred suspension of NaH (60% dispersion in mineral oil, 2.3 g, 57.5 mmol, 5.0 equiv) in THF (30 mL) was added dropwise a solution of **SI-20** (1.59 g, 11.5 mmol) in THF (70 mL) at 0 ºC, then *i*PrI (11.5 mL, 115 mmol, 10.0 equiv) was added. The reaction mixture was refluxed overnight. The resulting mixture was quenched at 0 °C by adding H_2O. Then 2 M HCl (40 mL) was added, and the mixture stirred at room temperature for 1 h. Then brine was added to the reaction mixture, and the organic layer was separated. The aqueous layer was extracted with ethyl acetate (3 × 50 mL). The combined organic layers were washed with brine (50 mL), dried over Na_2SO_4, and concentrated under reduced pressure. The resulting crude residue was purified by flash column chromatography (eluent: petroleum ether/CH_2Cl_2 = 5/1) to afford **SI-21** (1.44 g, 70%) as a colorless oil. R_f = 0.3 (petroleum ether/CH_2Cl_2 = 2/1).

Ketone **SI-21** (1.44 g, 8.0 mmol) was dissolved in dry THF (10 mL) and the mixture was cooled to -78 °C. LiHMDS (1.0 M in THF, 16.0 mL, 16.0 mmol, 2.0 equiv) was added and the clear solution was stirred at -40 ºC for 1 h. Then $PhNTf_2$ (5.72 g, 16.0 mmol, 2.0 equiv) in dry THF (20 mL) was slowly added at -78 ºC and the mixture was allowed to warm to room temperature and stirred for additional 4 h. Then brine (50 mL) was added to the reaction mixture, and the organic layer was separated. The aqueous layer was extracted with ethyl acetate (3 × 50 mL). The combined organic layers were dried over Na_2SO_4 and concentrated under reduced pressure. The residue was purified by flash chromatography (eluent: petroleum ether) to afford **15d**[10] (2.01 g, 80%) as a colorless oil. R_f= 0.4 (petroleum ether); ^{1}H NMR (600 MHz, $CDCl_3$) δ = 5.78–5.71 (m, 1H), 5.10–5.04 (m, 2H), 2.83 (sept. *J* = 6.6 Hz, 1H), 2.28–2.17 (m, 3H), 2.10 (ddt, *J* = 13.8, 8.4, 0.9 Hz, 1H), 1.95 (ddd, *J* = 12.6, 8.4, 4.8 Hz, 1H), 1.66 (ddd, *J* = 12.6, 9.0, 6.0 Hz, 1H), 1.14 (s, 3H), 1.03 (d, *J* = 6.6 Hz, 3H), 1.01 (d, *J* = 6.6 Hz, 3H).

7.1.4 Synthesis of 15e.

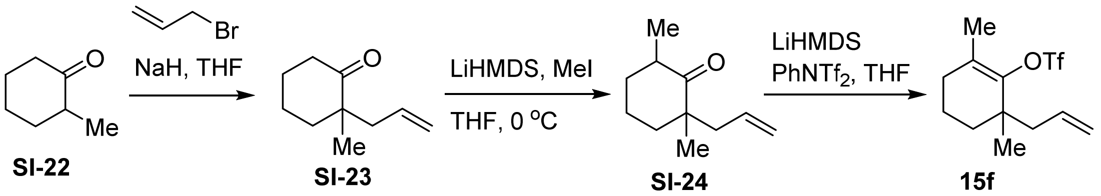

Supplementary Figure 40. Synthesis of compound **15f**

To a stirred suspension of NaH (60% dispersion in mineral oil, 0.2 g, 5 mmol, 1.0 equiv) in THF (5 mL) was added dropwise a solution of **SI-22** (0.61 mL, 5 mmol) in THF (5 mL) at 0 °C. The reaction mixture was refluxed 1 h. Allyl bromide (0.43 mL, 5 mmol, 1.0 equiv) was added at 0 °C, and the reaction mixture was refluxed 2 h. Then brine (30 mL) was added to the reaction mixture. The organic layer was separated and the aqueous layer was extracted with ethyl acetate (3 × 25 mL). The combined organic layers were dried over Na_2SO_4 and concentrated under reduced pressure. The residue was purified by flash chromatography (eluent: petroleum ether/EtOAc = 40/1) to afford **SI-23**[11] (0.23 g, 30% yield) as a colorless oil. R_f= 0.3 (petroleum ether/EtOAc = 15/1); ^{1}H NMR (300 MHz, $CDCl_3$) δ = 5.80–5.59 (m, 1H), 5.11–4.96 (m, 2H), 2.45–2.30 (m, 3H), 2.24 (dd, J = 13.8, 7.2 Hz, 1H), 1.93–1.50 (m, 6H), 1.07 (s, 3H).

Ketone **SI-23** (0.30 g, 2.0 mmol) was dissolved in dry THF (2 mL) and the mixture was cooled to 0 °C. LiHMDS (1.0 M in THF, 2.4 mL, 2.4 mmol, 1.2 equiv) was added and the reaction mixture was stirred at this temperature for 1 h. Then MeI (0.31 mL, 5.0 mmol, 2.5 equiv) was added slowly and the mixture was allowed to warm to room temperature and stirred for additional 5 h. Then brine (10 mL) was added to the reaction mixture. The organic layer was separated and the aqueous layer was extracted with ethyl acetate (3 × 15 mL). The combined organic layers were dried over Na_2SO_4 and concentrated under reduced pressure. The residue was purified by flash chromatography (eluent: petroleum ether/Et_2O = 50/1) to afford **SI-24** as a colorless oil. R_f= 0.4 (petroleum ether/Et_2O = 10/1).

Ketone **SI-24** was dissolved in dry THF (4 mL) and the mixture was cooled to -78 °C. LiHMDS (1.0 M in THF, 4.0 mL, 4.0 mmol) was added and the reaction mixture was stirred at -78 °C for 1 h. Then $PhNTf_2$ (0.86 g, 2.4 mmol) in dry THF (4 mL) was slowly added and the mixture was allowed to warm to room temperature and stirred for additional 3 h. Then brine (20 mL) was added to the reaction mixture. The organic layer was separated and the aqueous layer was extracted with ethyl acetate (3 × 20 mL). The combined organic layers were dried over Na_2SO_4 and concentrated under reduced pressure. The residue was purified by flash chromatography (eluent: petroleum ether) to afford **15e** (0.31 g, 52% over 2 steps) as a colorless oil. R_f= 0.2 (petroleum ether); ^{1}H NMR (300 MHz, $CDCl_3$) δ = 5.77–5.61 (m, 1H), 5.11–5.00 (m, 2H), 2.33

(ddt, J = 14.1, 6.9, 1.2 Hz, 1H), 2.18–2.08 (m, 3H), 1.79–1.58 (m, 6H), 1.49–1.40 (m, 1H), 1.16 (s, 3H).

7.1.5 Synthesis of 15f.

15f was prepared according to the reported procedure.[12]

7.2 General Procedure for Cross-coupling Reaction and Characterization Data

To a solution of vinyl triflate **15** (0.2 mmol) in the mixed solvent of DMF (1.0 mL) and EtOH (1.0 mL) was added arylboronic acid **16** (0.4 mmol, 2.0 equiv), palladacycle **18** (5.6 mg, 5 mmol%), and K_3PO_4 (127 mg, 0.6 mmol, 3.0 equiv) at room temperature under nitrogen. The resulting mixture was stirred at the same temperature until the vinyl triflate had disappeared as monitored by TLC. The reaction mixture was then poured into water and extracted with ethyl acetate (3 × 25 mL). The organic layer was combined, washed with brine, dried over Na_2SO_4, and concentrated under vacuum. The residue was purified by silica gel column chromatography to afford the desired cross-coupling product.

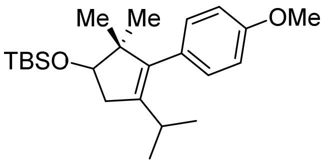

17b

17b: R_f = 0.5 (petroleum ether/CH_2Cl_2 = 5/2); Purified by column chromatography on silica gel with petroleum ether/CH_2Cl_2 (15/1) as eluent in 87% yield as oil. ^{1}H NMR (300 MHz, $CDCl_3$) δ 6.97 (d, J = 8.7 Hz, 2H), 6.85 (d, J = 8.7 Hz, 2H), 3.94 (t, J = 7.2 Hz, 1H), 3.81 (s, 3H), 2.43 (dd, J = 15.0, 7.2 Hz, 1H), 2.36 (sept, J = 6.9 Hz, 1H), 2.18 (dd, J = 15.0, 7.8 Hz, 1H), 0.93 (s, 3H), 0.92 (s, 9H), 0.91 (d, J = 6.9 Hz, 3H), 0.88 (d, J = 6.9 Hz, 3H), 0.86 (s, 3H), 0.09 (s, 3H), 0.08 (s, 3H); ^{13}C NMR (101 MHz, $CDCl_3$) δ 158.3, 141.9, 140.4, 130.5, 113.3, 109.7, 80.5, 55.3, 50.0, 36.4, 27.5, 26.0, 25.8, 21.5, 21.4, 19.9, 18.3, -4.2, -4.7; HRMS (EI) m/z calcd for $C_{23}H_{38}O_2Si$ [M]: 374.2641, found 374.2639.

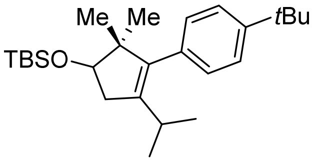

17c

17c: R_f = 0.4 (petroleum ether); Purified by column chromatography on silica gel with petroleum ether as eluent in 92% as oil; ^{1}H NMR (300 MHz, $CDCl_3$) δ 7.30 (d, J = 7.8 Hz, 2H), 6.97 (d, J = 7.8 Hz, 2H), 3.94 (t, J = 7.5 Hz, 1H), 2.43 (dd, J = 15.0, 7.5 Hz, 1H), 2.40

(sept, J = 6.9 Hz, 1H), 2.18 (dd, J = 15.0, 7.8 Hz, 1H), 1.33 (s, 9H), 0.95 (s, 3H), 0.92 (d, J = 6.9 Hz, 3H), 0.92 (s, 9H), 0.88 (d, J = 6.9 Hz, 3H), 0.86 (s, 3H), 0.09 (s, 3H), 0.08 (s, 3H); ^{13}C NMR (101 MHz, $CDCl_3$) δ 149.0, 142.3, 140.2, 135.1, 129.1, 124.7, 80.6, 50.0, 36.4, 34.6, 31.6, 27.5, 26.1, 25.8, 21.6, 21.4, 20.0, 18.3, -4.2, -4.7; HRMS (EI) m/z calcd for $C_{26}H_{44}OSi$ [M]: 400.3161, found 400.3157.

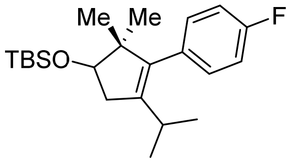

17d

17d: R$_f$ = 0.4 (petroleum ether); Purified by column chromatography on silica gel with petroleum ether as eluent in 94% as oil; ^{1}H NMR (300 MHz, $CDCl_3$) δ = 7.04–6.95 (m, 4H), 3.95 (t, J = 7.5 Hz, 1H), 2.45 (dd, J = 15.0, 7.2 Hz, 1H), 2.31 (sept, J = 6.9 Hz, 1H), 2.18 (dd, J = 15.3, 7.5 Hz, 1H), 0.93 (s, 3H), 0.92 (s, 9H), 0.91 (d, J = 6.9 Hz, 3H), 0.88 (d, J = 6.9 Hz, 3H), 0.85 (s, 3H), 0.09 (s, 3H), 0.08 (s, 3H); ^{13}C NMR (101 MHz, $CDCl_3$) δ 163.1, 160.7, 141.3, 141.1, 134.1, 131.0, 130.9, 114.9, 114.7, 80.4, 50.0, 36.5, 27.5, 26.0, 25.7, 21.5, 21.3, 19.9, 18.3, -4.2, -4.7; HRMS (EI) m/z calcd for $C_{22}H_{35}OFSi$ [M]: 362.2441, found 362.2438.

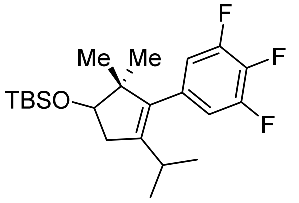

17e

17e: R$_f$ = 0.5 (petroleum ether); Purified by column chromatography on silica gel with petroleum ether as eluent in 75% as oil; ^{1}H NMR (300 MHz, $CDCl_3$) δ 6.72–6.58 (m, 2H), 3.92 (t, J = 7.2 Hz, 1H), 2.45 (dd, J = 15.3, 7.2 Hz, 1H), 2.31 (sept, J = 6.9 Hz, 1H), 2.17 (dd, J = 15.3, 7.2 Hz, 1H), 0.93 (d, J = 6.9 Hz, 3H), 0.93 (s, 3H), 0.92 (s, 9H), 0.89 (d, J = 6.9 Hz, 3H), 0.86 (s, 3H), 0.08 (s, 3H), 0.07(s, 3H); HRMS (EI) m/z calcd for $C_{22}H_{33}OF_3Si$ [M]: 398.2253, found 398.2259.

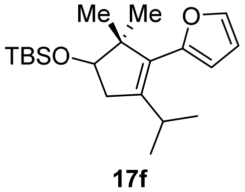

17f

17f: R$_f$ = 0.4 (petroleum ether); Purified by column chromatography on silica gel with

petroleum ether as eluent in 82% as oil; ^{1}H NMR (300 MHz, $CDCl_3$) δ 7.38 (d, *J* = 1.8 Hz, 1H), 6.40–6.35 (m, 1H), 6.17 (d, *J* = 3.3 Hz, 1H), 3.87 (t, *J* = 7.5 Hz, 1H), 2.93 (sept, *J* = 6.9 Hz, 1H), 2.47 (dd, *J* = 15.9, 7.2 Hz, 1H), 2.21 (dd, *J* = 15.9, 7.8 Hz, 1H), 1.08 (s, 3H), 1.05 (s, 3H), 1.04 (d, *J* = 6.9 Hz, 3H), 0.96 (d, *J* = 6.9 Hz, 3H), 0.91 (s, 9H), 0.07 (s, 3H), 0.07 (s, 3H); ^{13}C NMR (101 MHz, $CDCl_3$) δ 151.8, 144.0, 141.0, 131.8, 110.4, 107.8, 80.2, 49.5, 37.2, 28.0, 26.1, 25.6, 21.4, 21.1, 20.1, 18.3, -4.2, -4.7; HRMS (EI) m/z calcd for $C_{20}H_{34}O_2Si$ [M]: 334.2328, found 334.2322.

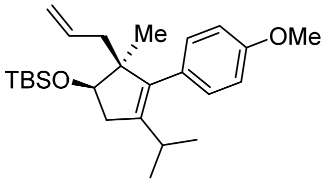

17g

17g: R*f* = 0.4 (petroleum ether/CH_2Cl_2 = 10/3); Purified by column chromatography on silica gel with petroleum ether/CH_2Cl_2 (15/1) as eluent in 75% as oil; ^{1}H NMR (300 MHz, $CDCl_3$) δ 7.01 (d, *J* = 8.7 Hz, 2H), 6.86 (d, *J* = 8.7 Hz, 2H), 6.08–5.90 (m, 1H), 5.05–4.86 (m, 2H), 4.05 (t, *J* = 7.8 Hz, 1H), 3.81 (s, 3H), 2.48–2.34 (m, 2H), 2.32–2.16 (m, 2H), 2.05 (dd, *J* = 13.8, 7.5 Hz, 1H), 0.93 (s, 9H), 0.89 (d, *J* = 6.9 Hz, 3H), 0.89 (d, *J* = 6.9 Hz, 3H), 0.89 (s, 3H), 0.09 (s, 3H), 0.09 (s, 3H); ^{13}C NMR (101 MHz, $CDCl_3$) δ 158.3, 142.3, 139.2, 137.6, 130.7, 130.4, 115.7, 113.3, 80.8, 55.3, 53.6, 40.3, 37.6, 27.6, 26.0, 25.8, 21.7, 21.4, 18.2, -4.2, -4.7; HRMS (EI) m/z calcd for $C_{25}H_{40}O_2Si$ [M]: 400.2798, found 400.2794.

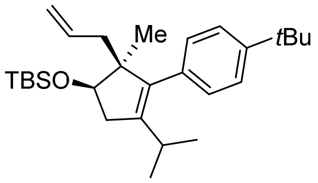

17h

17h: R*f* = 0.6 (petroleum ether); Purified by column chromatography on silica gel with petroleum ether as eluent in 85% as oil; ^{1}H NMR (300 MHz, $CDCl_3$) δ 7.30 (d, *J* = 8.4 Hz, 2H), 7.01 (d, *J* = 8.4 Hz, 2H), 6.10–5.92 (m, 1H), 5.07–4.86 (m, 2H), 4.06 (t, *J* = 7.8 Hz, 1H), 2.50–2.35 (m, 2H), 2.35–2.16 (m, 2H), 2.08 (dd, *J* = 14.1, 7.5 Hz, 1H), 1.33 (s, 9H), 0.94 (s, 9H), 0.90 (d, *J* = 6.9 Hz, 3H), 0.89 (d, *J* = 6.9 Hz, 3H), 0.89 (s, 3H), 0.10 (s, 3H), 0.09 (s, 3H); ^{13}C NMR (101 MHz, $CDCl_3$) δ 149.0, 142.2, 139.5, 137.6, 135.0, 129.2, 124.7, 115.6, 80.9, 53.7, 40.3, 37.7, 34.6, 31.6, 27.6, 26.1, 25.9, 21.8, 21.5, 18.3, -4.1, -4.7; HRMS (EI) m/z calcd for $C_{28}H_{46}OSi$ [M]: 426.3318, found 426.3314.

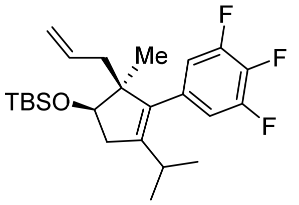

17i

17i: R_f = 0.7 (petroleum ether); Purified by column chromatography on silica gel with petroleum ether as eluent in 81% as oil; ^{1}H NMR (300 MHz, $CDCl_3$) δ 6.77–6.62 (m, 2H), 6.02–5.84 (m, 1H), 5.05–4.90 (m, 2H), 4.04 (t, *J* = 7.5 Hz, 1H), 2.45 (dd, *J* = 15.9, 7.8 Hz, 1H), 2.40–2.16 (m, 3H), 2.00 (dd, *J* = 14.1, 7.8 Hz, 1H), 0.93 (s, 9H), 0.91 (d, *J* = 6.9 Hz, 3H), 0.91 (d, *J* = 6.9 Hz, 3H), 0.89 (s, 3H), 0.09 (s, 3H), 0.09 (s, 3H); ^{13}C NMR (101 MHz, $CDCl_3$) δ 150.9 (dd, *J* = 250.6, 5.4 Hz), 144.5, 138.7 (dt, *J* = 250.8, 15.6 Hz), 137.1, 137.0, 134.4–138.8 (m), 116.3, 80.4, 53.8, 40.3, 37.8, 27.8, 26.0, 25.7, 21.6, 21.3, 18.2, -4.2, -4.8; HRMS (EI) m/z calcd for $C_{24}H_{35}OF_3Si$ [M]: 424.2409, found 424.2406.

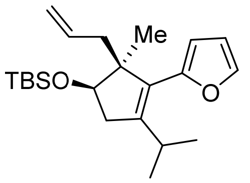

17j

17j: R_f = 0.5 (petroleum ether); Purified by column chromatography on silica gel with petroleum ether as eluent in 85% yield as oil; ^{1}H NMR (600 MHz, $CDCl_3$) δ 7.38 (d, *J* = 1.2 Hz, 1H), 6.82–6.36 (m, 1H), 6.17 (d, *J* = 3.0 Hz, 1H), 5.95–5.86 (m, 1H), 4.97-4.91 (m, 1H), 4.87–4.83 (m,1H), 3.99 (t, *J* = 7.8 Hz, 1H), 2.95 (sept, *J* = 6.6 Hz, 1H), 2.46 (dd, *J* = 16.2, 7.8 Hz, 1H), 2.39 (dd, *J* = 13.2, 7.2 Hz, 1H), 2.27 (dd, *J* = 13.2, 7.2 Hz, 1H), 2.22 (dd, *J* = 15.6, 7.8 Hz, 1H), 1.06 (s, 3H), 1.02 (d, *J* = 6.6 Hz, 3H), 0.95 (d, *J* = 6.6 Hz, 3H), 0.94 (s, 9 H), 0.09 (s, 3H), 0.08 (s, 3H); ^{13}C NMR (151 MHz, $CDCl_3$) δ 151.7, 145.8, 141.0, 137.1, 129.2, 115.9, 110.4, 108.1, 80.4, 53.1, 40.2, 38.3, 28.1, 26.0, 25.6, 21.5, 21.3, 18.2, -4.2, -4.7. HRMS (EI) m/z calcd for $C_{22}H_{36}O_2Si$ [M]: 360.2485, found 360.2480.

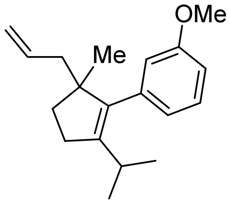

17k

17k: R_f = 0.4 (petroleum ether/CH_2Cl_2 = 4/1); Purified by column chromatography on silica

gel with petroleum ether/CH_2Cl_2 (15/1) as eluent in 80% as oil; ^{1}H NMR (300 MHz, $CDCl_3$) δ 7.26–7.16 (m, 1H), 6.85–6.75 (m, 1H), 6.70–6.55 (m, 2H), 5.90–5.70 (m, 1H), 5.06–4.93 (m, 2H), 3.81 (s, 3H), 2.44–2.22 (m, 3H), 2.17–2.04 (m, 1H), 2.03–1.85 (m, 2H), 1.69–1.55 (m, 1H), 1.01 (s, 3H), 0.93 (d, J = 7.2 Hz, 3H), 0.93 (d, J = 7.2 Hz, 3H); ^{13}C NMR (101 MHz, $CDCl_3$) δ 159.2, 145.1, 142.0, 140.0, 136.5, 128.7, 122.4, 116.6, 115.8, 111.5, 55.3, 51.3, 44.8, 36.0, 28.2, 27.9, 26.4, 21.8, 21.5; HRMS (EI) m/z calcd for $C_{19}H_{26}O$ [M]: 270.1984, found 270.1987.

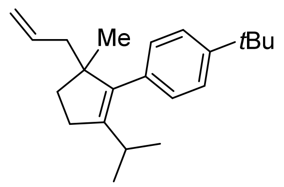

17l

17l: R_f = 0.7 (petroleum ether); Purified by column chromatography on silica gel with petroleum ether as eluent in 83% as oil; ^{1}H NMR (300 MHz, $CDCl_3$) δ 7.30 (d, J = 8.4 Hz, 2H), 6.97 (d, J = 8.4 Hz, 2H), 5.90–5.72 (m, 1H), 5.06–4.93 (m, 2H), 2.45–2.23 (m, 3H), 2.15–2.04 (m, 1H), 2.02–1.84 (m, 2H), 1.68–1.52 (m, 1H), 1.33 (s, 9H), 1.00 (s, 3H), 0.93 (d, J = 6.9 Hz, 3H), 0.90 (d, J = 6.9 Hz, 3H); ^{13}C NMR (101 MHz, $CDCl_3$) δ 148.9, 144.8, 142.1, 136.7, 135.3, 129.3, 124.7, 116.4, 51.3, 44.8, 35.9, 34.6, 31.6, 28.1, 27.8, 26.3, 21.8, 21.6; HRMS (EI) m/z calcd for $C_{22}H_{32}$ [M]: 296.2504, found 296.2510.

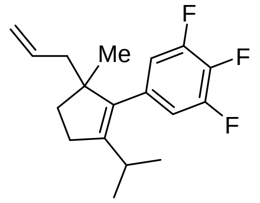

17m

17m: R_f = 0.7 (petroleum ether); Purified by column chromatography on silica gel with petroleum ether as eluent in 71% as oil; ^{1}H NMR (300 MHz, $CDCl_3$) δ 6.73–6.60 (m, 2H), 5.84–5.65 (m, 1H), 5.08–4.95 (m, 2H), 2.38–2.24 (m, 3H), 2.10–1.85 (m, 3H), 1.73–1.56 (m, 1H), 1.00 (s, 3H), 0.94 (d, J = 6.9 Hz, 3H), 0.90 (d, J = 6.9 Hz, 3H); ^{13}C NMR (101 MHz, $CDCl_3$) δ 150.9 (ddd, J = 250.1, 8.8, 4.2 Hz), 147.3, 138.7 (dt, J = 251.5, 15.6 Hz), 138.0, 135.6, 134.8–134.2 (m), 117.2, 113.8 (dd, J = 15.4, 5.6 Hz), 51.3, 44.7, 35.8, 28.3, 27.9, 26.3, 21.6, 21.4; HRMS (EI) m/z calcd for $C_{18}H_{21}F_3$ [M]: 294.1595, found 294.1597.

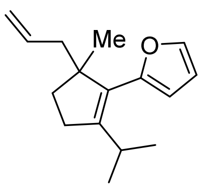

17n

7n: R$_f$ = 0.6 (petroleum ether); Purified by column chromatography on silica gel with petroleum ether as eluent in 83% as oil; ^{1}H NMR (300 MHz, $CDCl_3$) δ = 7.39 (d, *J* = 1.8 Hz, 1H), 6.42–6.34 (m, 1H), 6.17 (d, *J* = 3.3 Hz, 1H), 5.82–5.64 (m, 1H), 5.03–4.90 (m, 2H), 2.94 (sept, *J* = 6.9 Hz, 1H), 2.38–2.27 (m, 3H), 2.22–2.11 (m, 1H), 1.95–1.82 (m, 1H), 1.62–1.49 (m, 1H), 1.16 (s, 3H), 1.02 (d, *J* = 6.9 Hz, 3H), 1.01 (d, *J* = 6.9 Hz, 3H); ^{13}C NMR (101 MHz, $CDCl_3$) δ 151.9, 149.1, 140.9, 136.5, 131.4, 116.5, 110.4, 107.8, 50.8, 44.8, 36.0, 29.0, 28.2, 26.4, 21.6, 21.3; HRMS (EI) m/z calcd for $C_{16}H_{22}O$ [M]: 230.1671, found 230.1667.

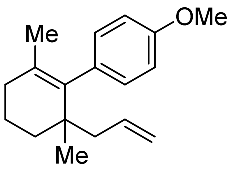

17o

17o: R$_f$ = 0.3 (petroleum ether/CH_2Cl_2 = 10/3); Purified by column chromatography on silica gel with petroleum ether/CH_2Cl_2 (15/1) as eluent in 75% as oil; ^{1}H NMR (400 MHz, $CDCl_3$) δ 6.92 (d, *J* = 8.4 Hz, 2H), 6.84 (d, *J* = 8.4 Hz, 2H), 5.83–5.69 (m, 1H), 5.02–4.89 (m, 2H), 3.81 (s, 3H), 2.17–1.90 (m, 4H), 1.75–1.62 (m, 3H), 1.47–1.37 (m, 1H), 1.30 (s, 3H), 0.92 (s, 3H); ^{13}C NMR (101 MHz, $CDCl_3$) δ 157.8, 140.2, 136.1, 133.8, 131.4, 130.7, 116.7, 113.0, 55.3, 44.8, 37.5, 35.4, 32.2, 26.5, 21.7, 19.2; HRMS (EI) m/z calcd for $C_{18}H_{24}O$ [M]: 256.1827, found 256.1822.

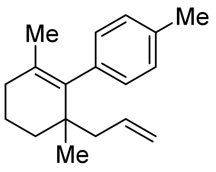

17p

17p: R$_f$ = 0.7 (petroleum ether); Purified by column chromatography on silica gel with petroleum ether as eluent in 83% as oil; ^{1}H NMR (300 MHz, $CDCl_3$) δ 0.98 (d, *J* = 7.8 Hz, 2H), 0.89 (d, *J* = 7.8 Hz, 2H), 5.84–5.67 (m, 1H), 5.03–4.88 (m, 2H), 2.34 (s, 3H), 2.15–1.89 (m, 4H), 1.75–1.61 (m, 3H), 1.48–1.37 (m, 1H), 1.29 (s, 3H), 0.93 (s, 3H); ^{13}C NMR (151

MHz, $CDCl_3$) δ 140.5, 138.5, 136.2, 135.3, 130.4, 130.2, 128.4, 116.7, 44.8, 37.3, 35.4, 32.2, 26.5, 21.7, 21.3, 19.2; HRMS (EI) m/z calcd for $C_{18}H_{24}$ [M]: 240.1878, found 240.1880.

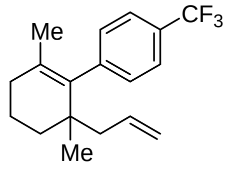

17q

17q: R_f = 0.7 (petroleum ether); Purified by column chromatography on silica gel with petroleum ether as eluent in 70% as oil; ^{1}H NMR (300 MHz, $CDCl_3$) δ 7.55 (d, J = 7.8 Hz, 2H), 7.14 (d, J = 7.8 Hz, 2H), 5.83–5.66 (m, 1H), 5.05–4.89 (m, 2H), 2.19–1.84 (m, 4H), 1.78–1.62 (m, 3H), 1.50–1.40 (m, 1H), 1.27 (s, 3H), 0.93 (s, 3H); ^{13}C NMR (151 MHz, $CDCl_3$) δ 145.6, 139.7, 135.5, 131.2, 130.8, 128.3 (q, J = 32.5 Hz), 124.6 (q, J = 272.1 Hz), 124.6, 117.2, 44.7, 37.3, 35.2, 32.1, 26.4, 21.6, 19.0; HRMS (EI) m/z calcd for $C_{18}H_{21}F_3$ [M]: 294.1595, found 294.1598.

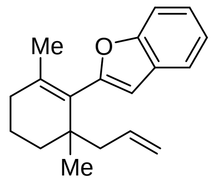

17r

17r: R_f = 0.4 (petroleum ether); Purified by column chromatography on silica gel with petroleum ether as eluent in 81% as oil; ^{1}H NMR (300 MHz, $CDCl_3$) δ 7.58–7.50 (m, 1H), 7.50–7.41 (m, 1H), 7.26–7.16 (m, 2H), 6.43 (d, J = 0.9 Hz, 1H), 5.88–5.70 (m, 1H), 5.05–4.89 (m, 2H), 2.29–1.97 (m, 4H), 1.78–1.65 (m, 3H), 1.59 (s, 3H), 1.49–1.36 (m, 1H), 1.07 (s, 3H); ^{13}C NMR (151 MHz, $CDCl_3$) δ 156.3, 154.4, 139.3, 135.8, 131.0, 128.9, 123.4, 122.5, 120.5, 117.0, 111.2, 105.7, 45.1, 37.5, 34.8, 32.3, 26.6, 22.1, 18.8; HRMS (EI) m/z calcd for $C_{19}H_{22}O$ [M]: 266.1671, found 266.1667.

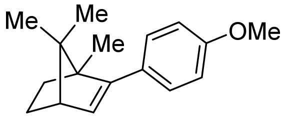

17s

17s: R_f = 0.3 (petroleum ether/CH_2Cl_2 = 10/3); Purified by column chromatography on silica gel with petroleum ether/CH_2Cl_2 (15/1) as eluent in 80% as oil; ^{1}H NMR (300 MHz, $CDCl_3$) δ = 7.19 (d, J = 8.4 Hz, 2H), 6.85 (d, J = 8.4 Hz, 2H), 5.91 (d, J = 3.3 Hz, 1H), 3.80 (s, 3H),

2.41–2.31 (m, 1H), 2.00–1.85 (m, 1H), 1.71–1.58 (m, 1H), 1.35–1.20 (m, 1H), 1.15–1.00 (m, 1H), 1.09 (s, 3H), 0.88 (s, 3H), 0.82 (s, 3H); ^{13}C NMR (151 MHz, $CDCl_3$) δ 158.5, 149.4, 131.3, 130.7, 127.9, 113.7, 57.2, 55.4, 55.0, 51.7, 32.1, 25.9, 20.0, 19.8, 12.9; HRMS (EI) m/z calcd for $C_{17}H_{22}O$ [M]: 242.1671, found 242.1670.

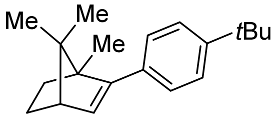

17t

17t: R_f = 0.6 (petroleum ether); Purified by column chromatography on silica gel with petroleum ether as eluent in 80% as oil; ^{1}H NMR (300 MHz, $CDCl_3$) δ 7.33 (d, J = 7.8 Hz, 2H), 7.22 (d, J = 7.8 Hz, 2H), 5.98 (d, J = 3.3 Hz, 1H), 2.41–2.33 (m, 1H), 2.01–1.84 (m, 1H), 1.73–1.58 (m, 1H), 1.35–1.22 (m, 1H), 1.32 (s, 9H), 1.13 (s, 3H), 1.15–1.01 (m, 1H), 0.88 (s, 3H), 0.82 (s, 3H); ^{13}C NMR (151 MHz, $CDCl_3$) δ 149.6, 149.4, 135.8, 131.5, 126.4, 125.1, 57.2, 55.0, 51.8, 34.6, 32.1, 31.5, 25.9, 19.9, 19.8, 12.9; HRMS (EI) m/z calcd for $C_{20}H_{28}$ [M]: 268.2191, found 268.2195.

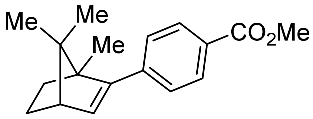

17u

17u: R_f = 0.4 (petroleum ether/CH_2Cl_2 = 1/1); Purified by column chromatography on silica gel with petroleum ether/CH_2Cl_2 (4/1) as eluent in 79% as oil; ^{1}H NMR (300 MHz, $CDCl_3$) δ = 7.96 (d, J = 8.1 Hz, 2H), 7.32 (d, J = 8.1 Hz, 2H), 6.11 (d, J = 3.3 Hz, 1H), 3.91 (s, 3H), 2.47–2.37 (m, 1H), 2.04–1.89 (m, 1H), 1.77–1.63 (m, 1H), 1.36–1.22 (m, 1H), 1.15–1.05 (m, 1H), 1.12 (s, 3H), 0.88 (s, 3H), 0.83 (s, 3H); ^{13}C NMR (151 MHz, $CDCl_3$) δ 167.2, 149.3, 143.6, 134.0, 129.6, 128.0, 126.5, 57.3, 55.1, 52.1, 51.9, 32.0, 25.7, 19.8, 19.7, 12.7; HRMS (EI) m/z calcd for $C_{18}H_{22}O_2$ [M]: 270.1620, found 270.1623.

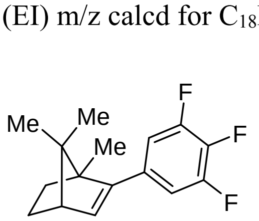

17v

17v: R_f = 0.8 (petroleum ether); Purified by column chromatography on silica gel with petroleum ether as eluent in 75% as oil; ^{1}H NMR (300 MHz, $CDCl_3$) δ 6.93–6.73 (m, 2H), 6.09–5.94 (m, 1H), 2.46–2.32 (m, 1H), 2.04–1.83 (m, 1H), 1.77–1.59 (m, 1H), 1.30–1.15 (m 1H), 1.14–1.00 (m, 1H), 1.09 (s, 3H), 0.85 (s, 3H), 0.80 (s, 3H); ^{13}C NMR (151 MHz, $CDCl_3$)

δ 151.1 (ddd, *J* = 248.6, 10.1, 4.4 Hz), 147.5, 138.6 (dt, *J* = 250.2, 15.6 Hz), 134.8–134.6 (m), 134.0, 110.5 (dd, *J* = 16.3, 4.5 Hz), 57.5, 55.2, 51.8, 32.0, 25.7, 19.8, 19.6, 12.6; HRMS (EI) m/z calcd for $C_{16}H_{17}F_3$ [M]: 266.1282, found 266.1280.

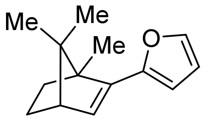

17w

17w: R_f = 0.7 (petroleum ether); Purified by column chromatography on silica gel with petroleum ether as eluent in 55% as oil; ^{1}H NMR (300 MHz, $CDCl_3$) δ = 7.34 (d, *J* = 1.8 Hz, 1H), 6.39–6.20 (m, 3H), 2.45–2.34 (m, 1H), 1.99–1.85 (m, 1H), 1.66–1.54 (m, 1H), 1.25 (s, 3H), 1.18–1.00 (m, 2H), 0.85 (s, 3H), 0.82 (s, 3H); ^{13}C NMR (151 MHz, $CDCl_3$) δ 151.9, 141.2, 139.1, 130.3, 111.0, 105.3, 57.1, 54.3, 51.7, 32.0, 25.7, 19.8, 19.7, 13.2.

8. Copies of NMR Spectra of Coupling Products

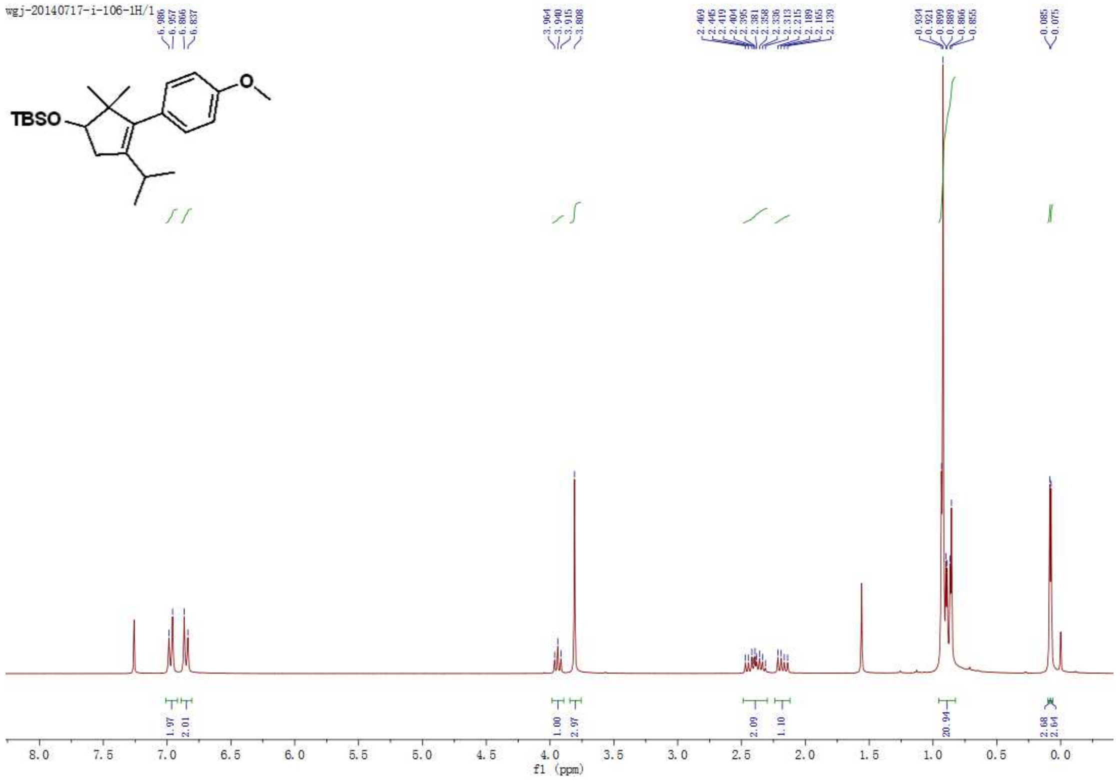

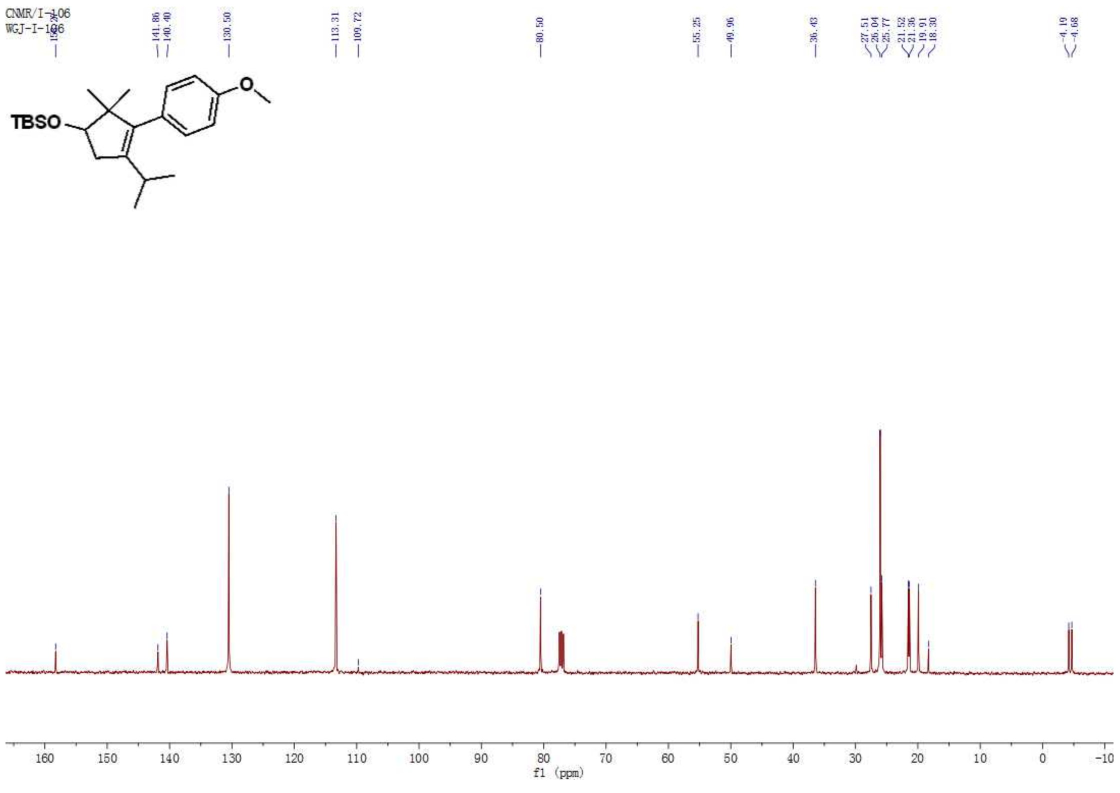

Supplementary Figure 41. ^{1}H and ^{13}C NMR of compound **17b**

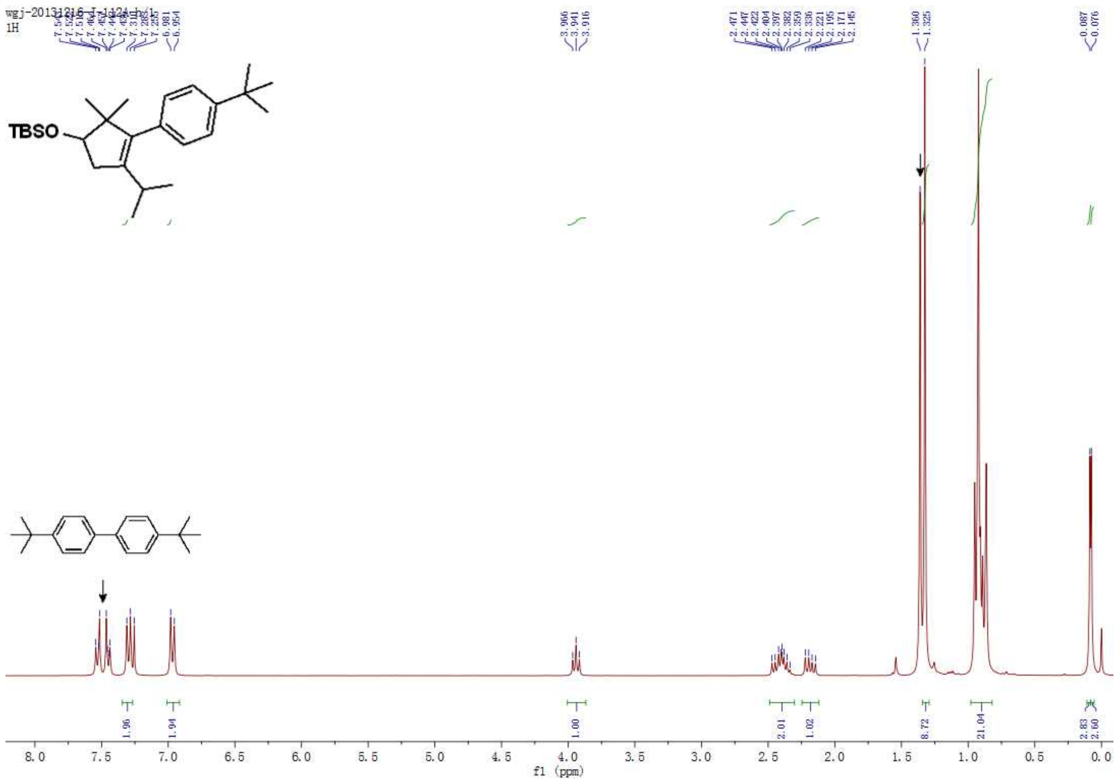

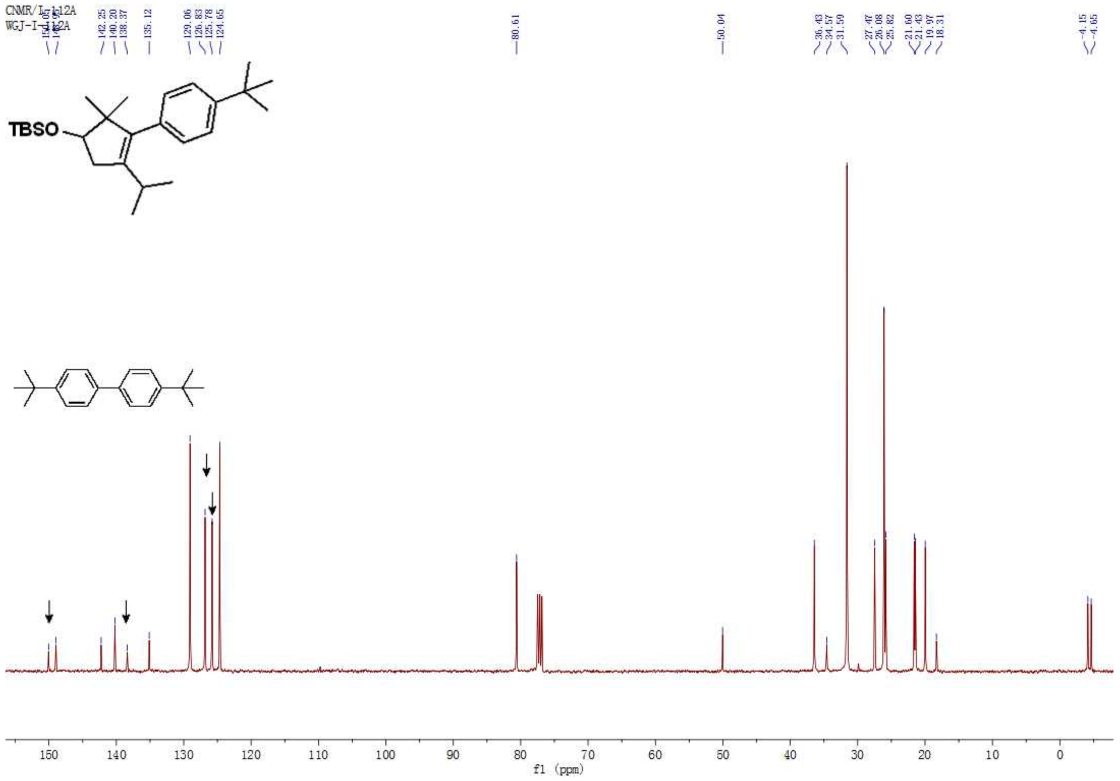

Supplementary Figure 42. ^{1}H and ^{13}C NMR of compound **17c**

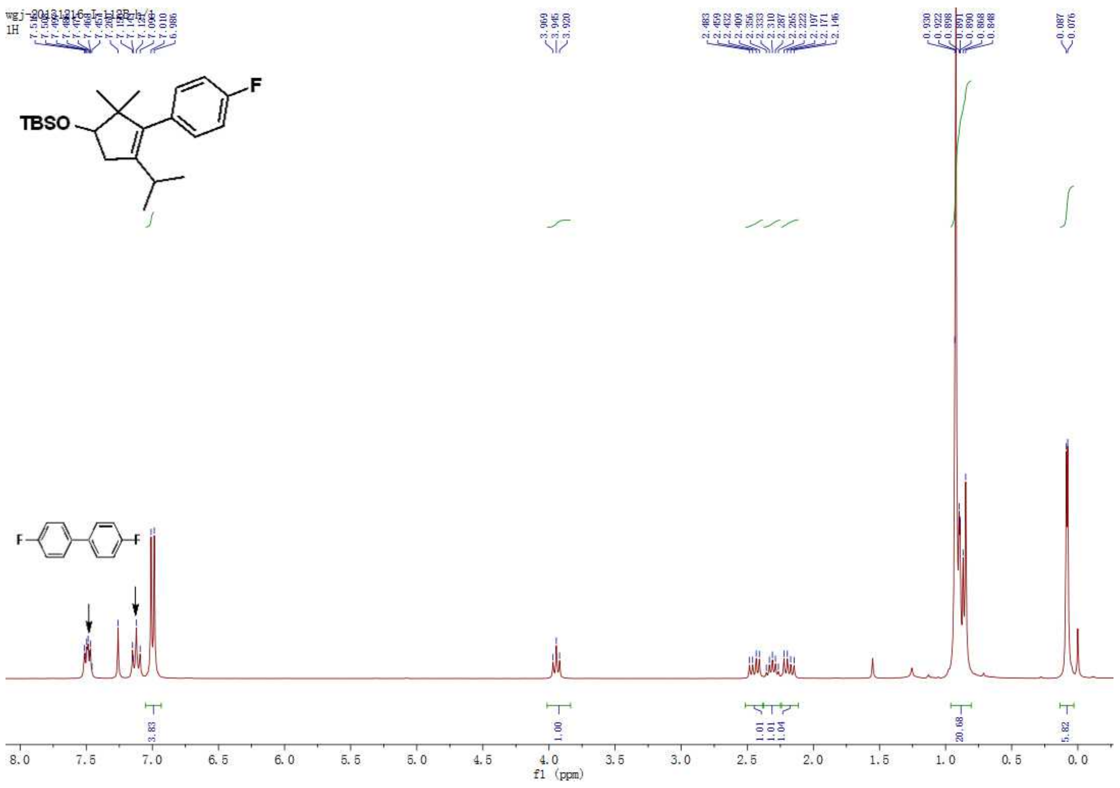

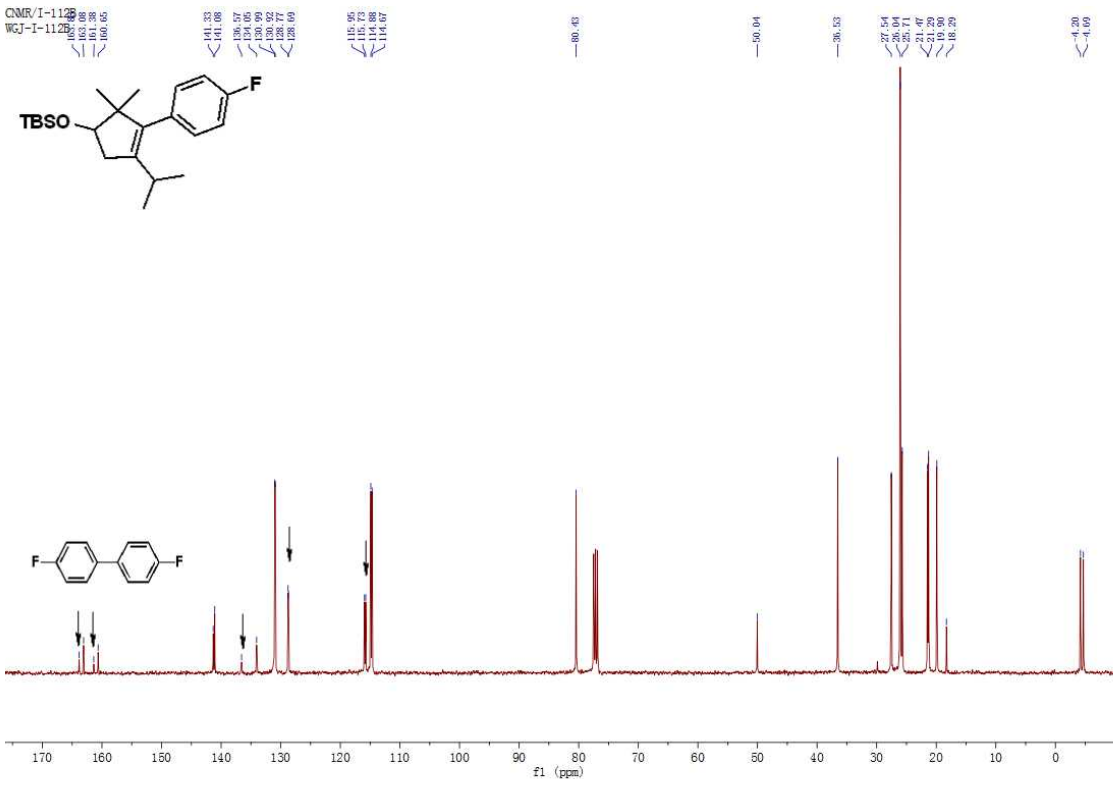

Supplementary Figure 43. ^{1}H and ^{13}C NMR of compound **17d**

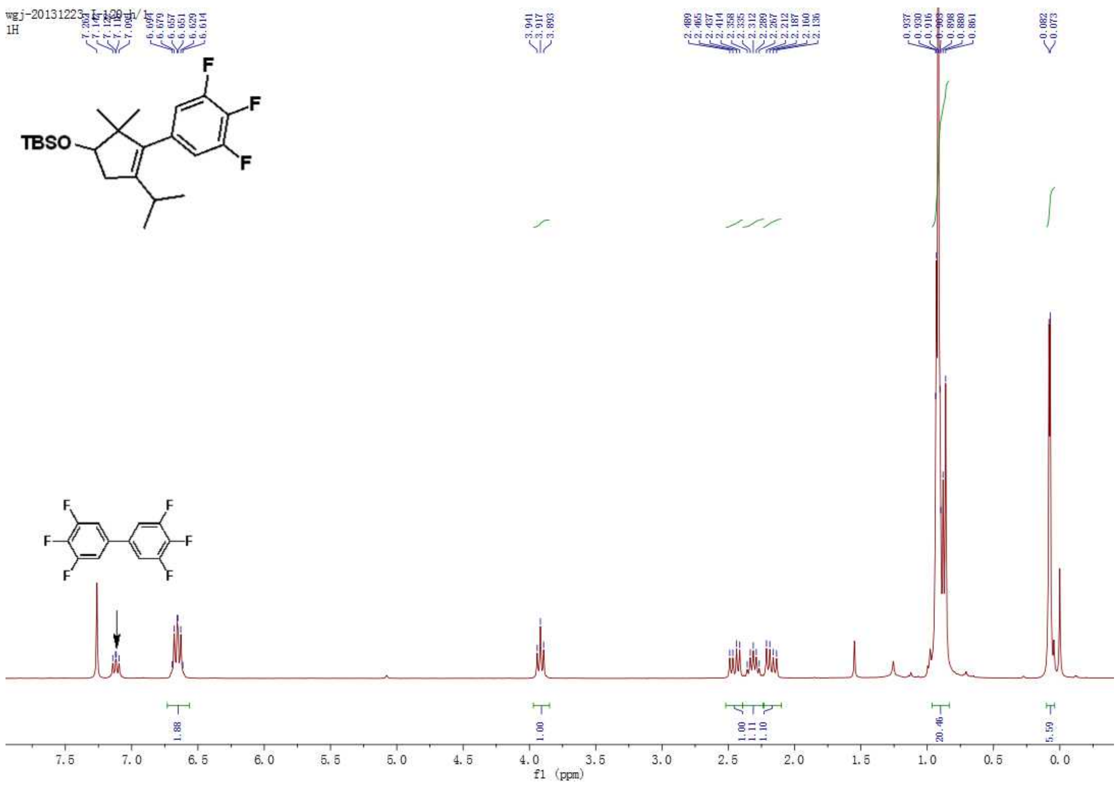

Supplementary Figure 44. ^{1}H NMR of compound **17e**

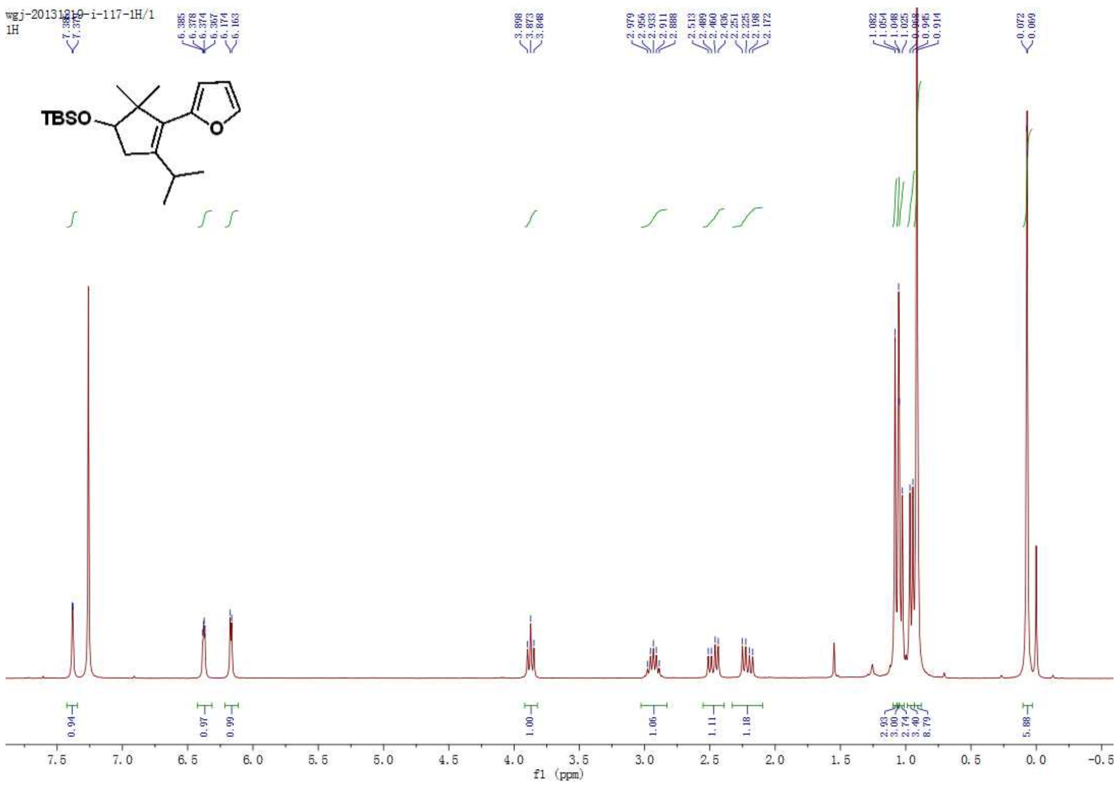

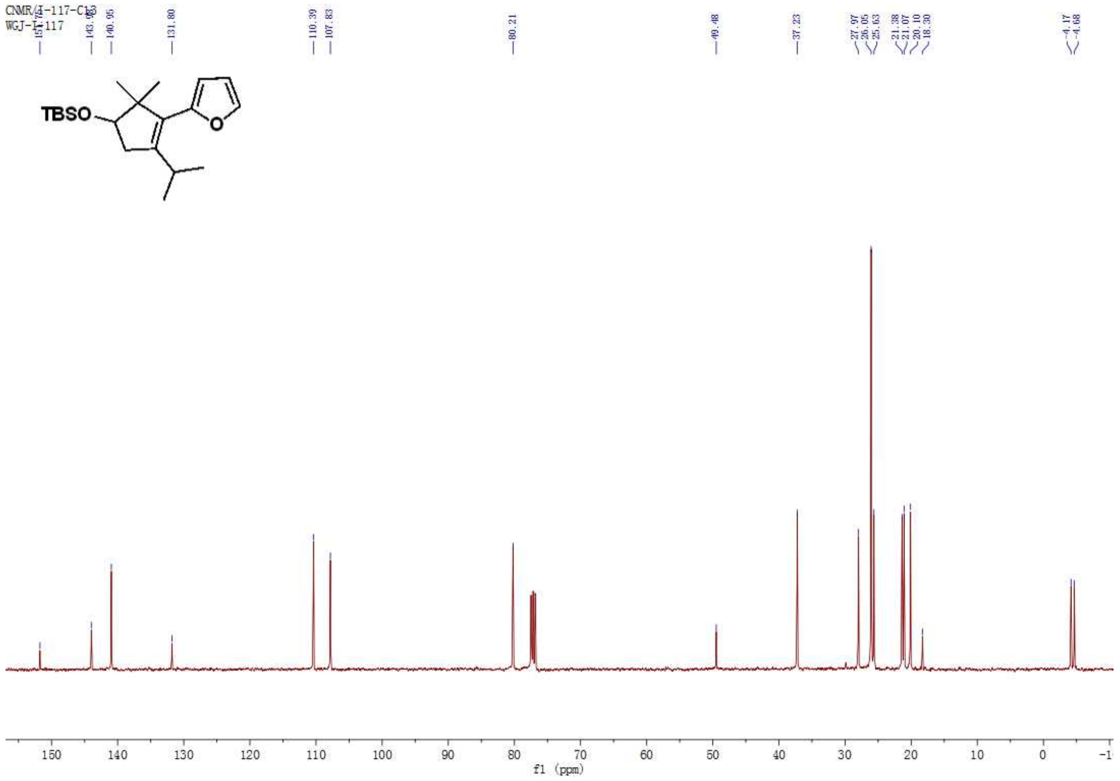

Supplementary Figure 45. ^{1}H and ^{13}C NMR of compound **17f**

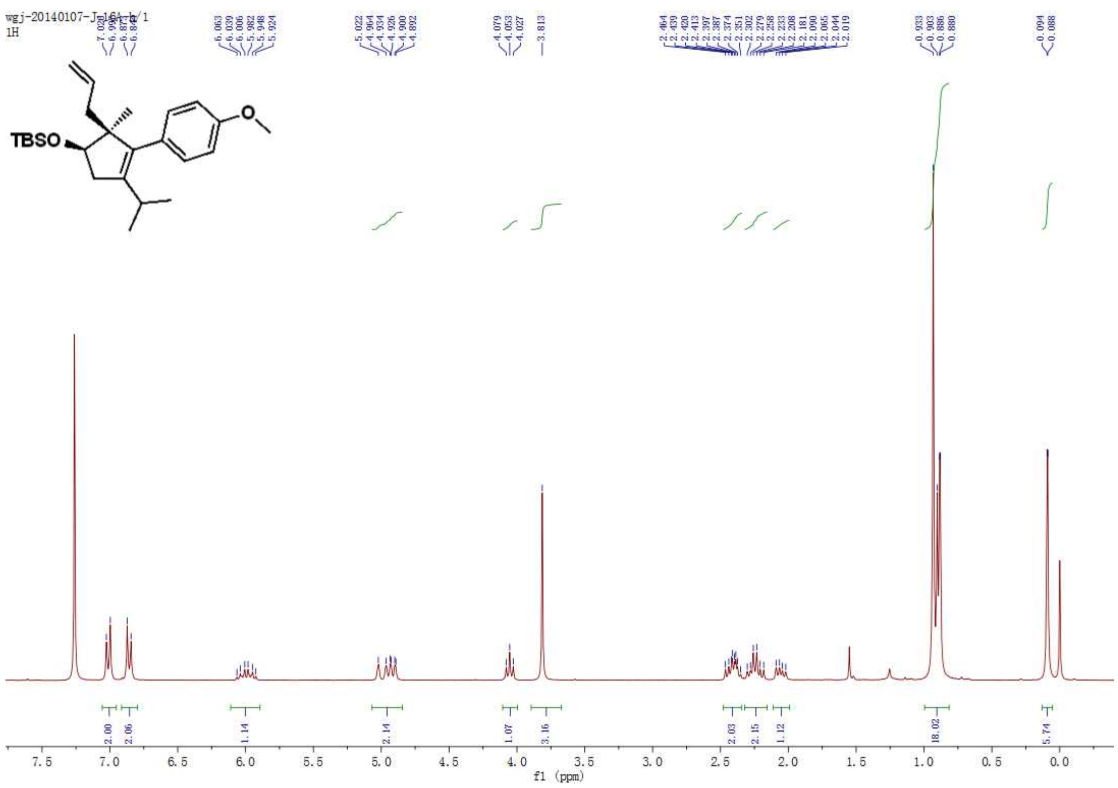

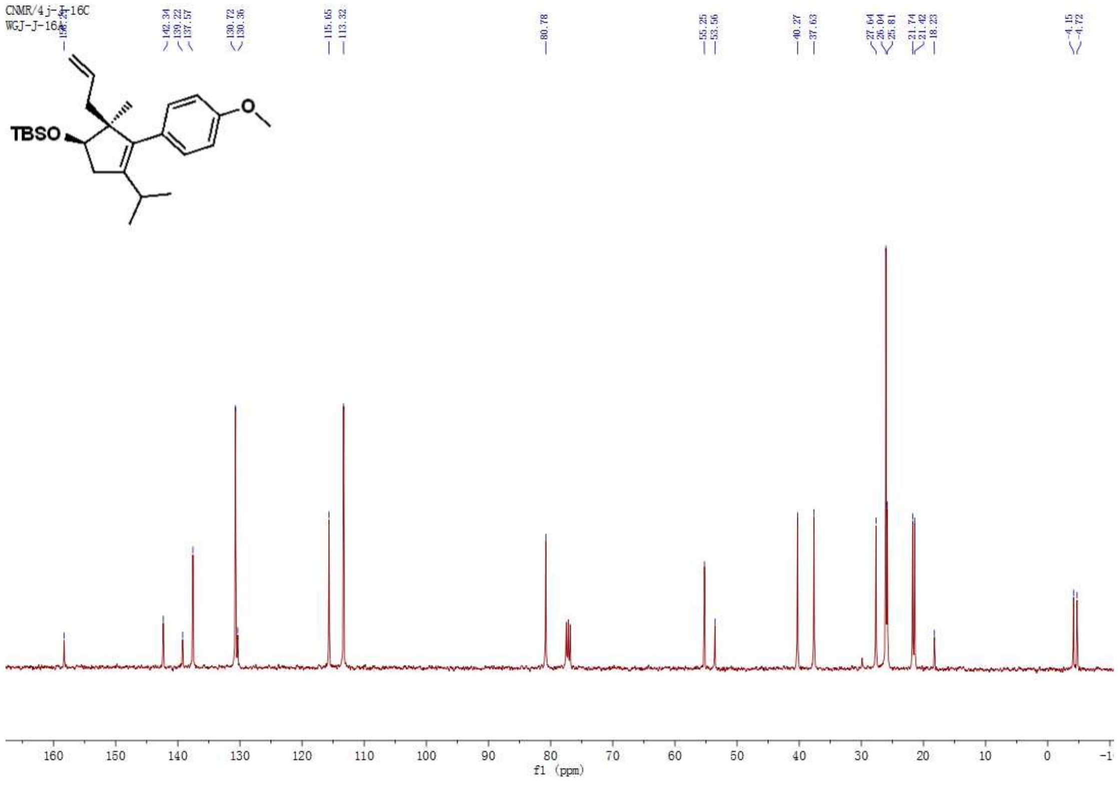

Supplementary Figure 46. ^{1}H and ^{13}C NMR of compound **17g**

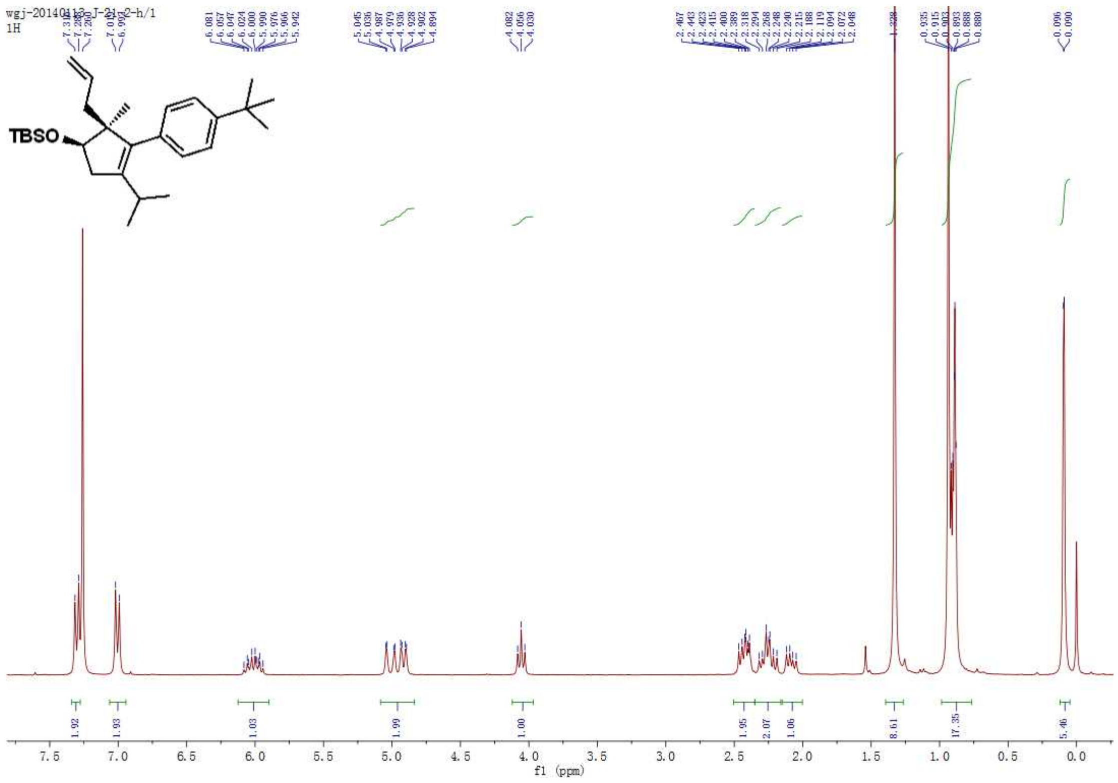

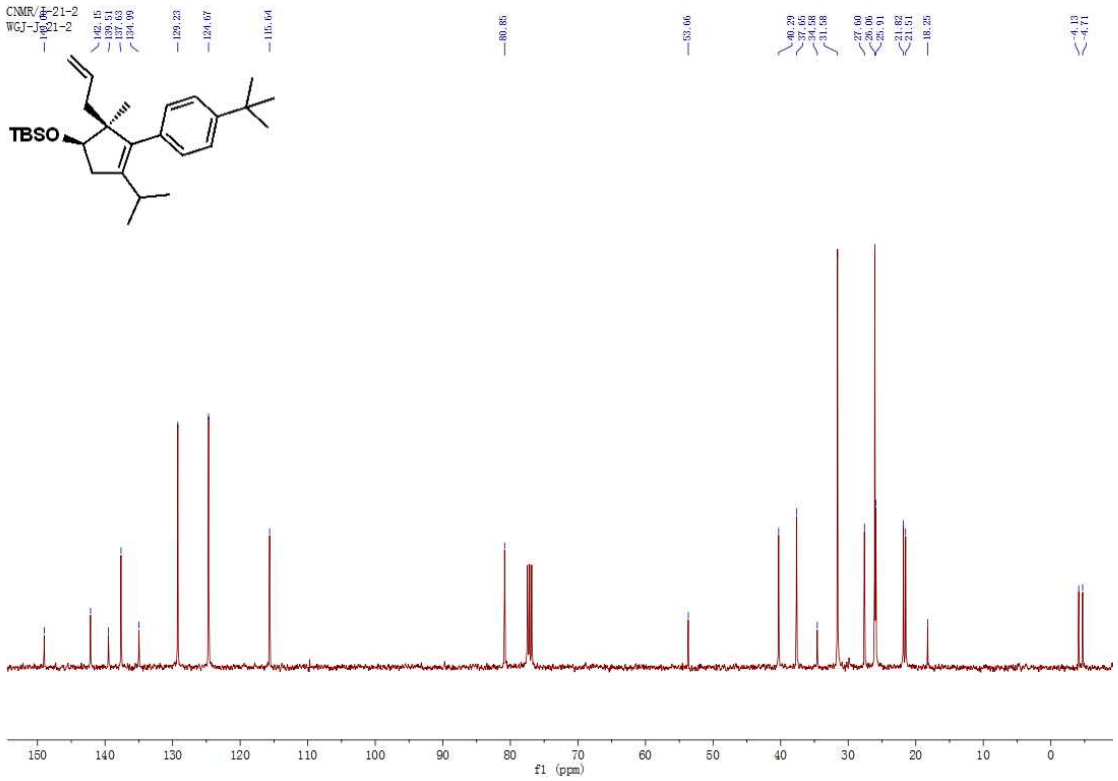

Supplementary Figure 47. ^{1}H and ^{13}C NMR of compound **17h**

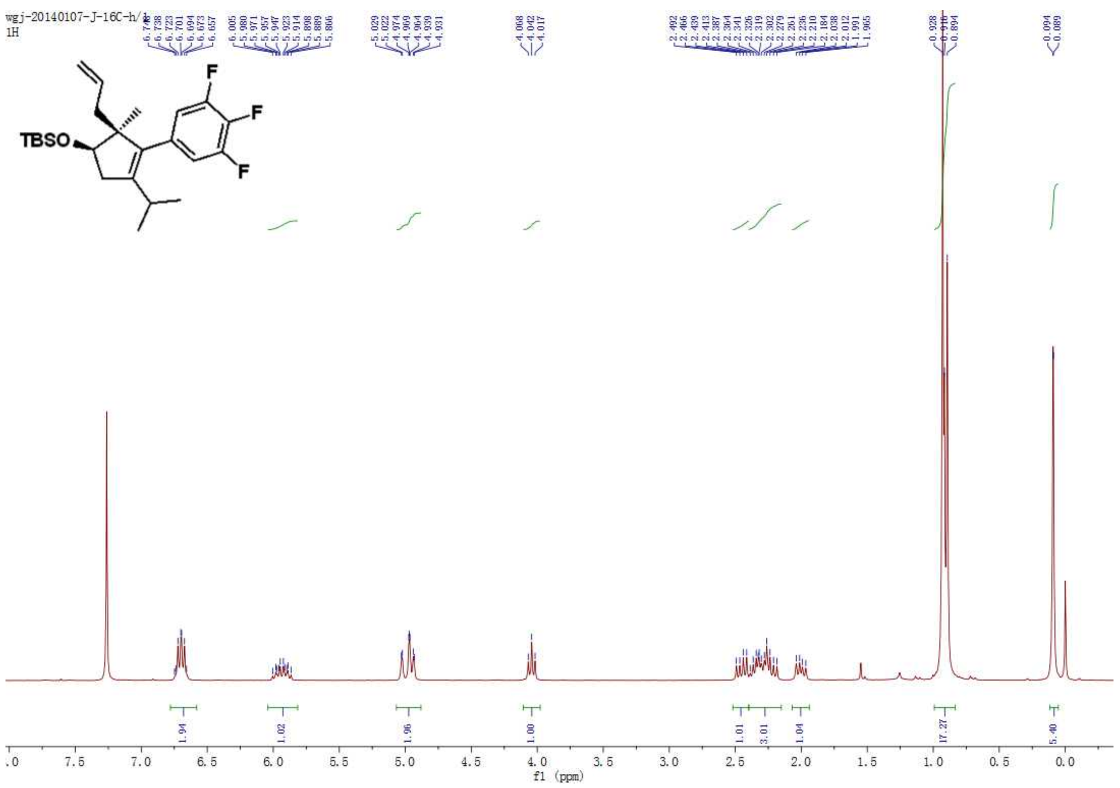

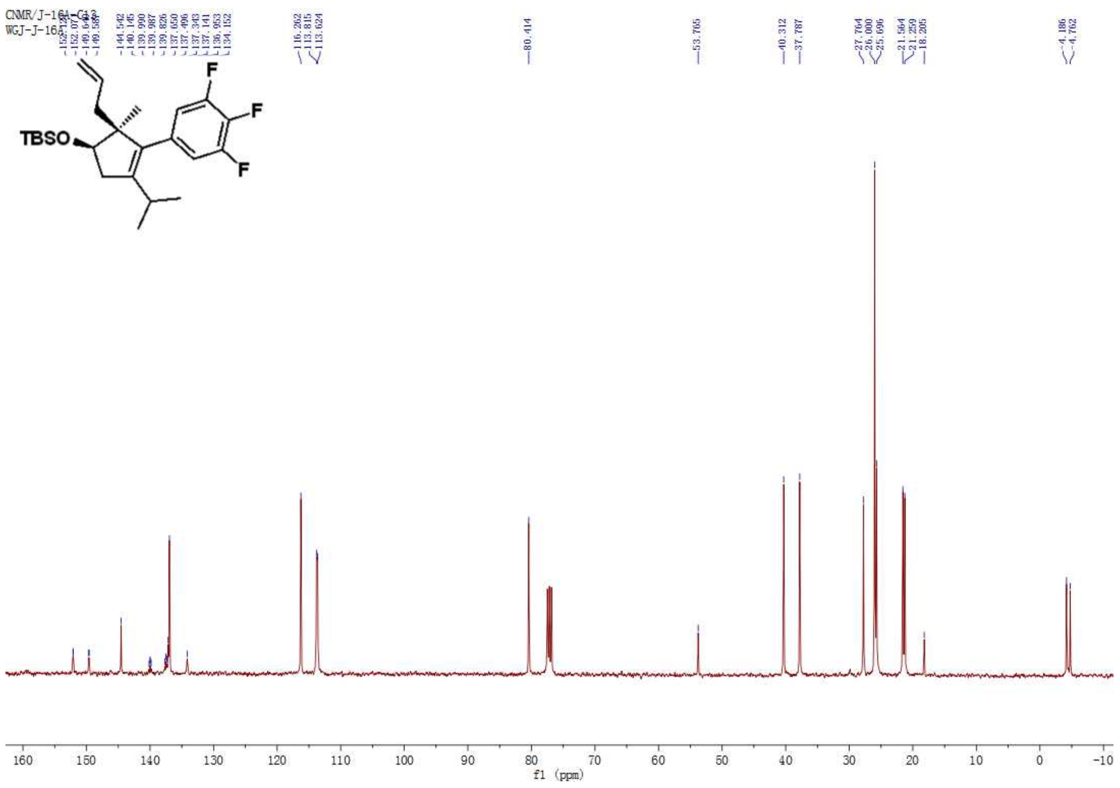

Supplementary Figure 48. ^{1}H and ^{13}C NMR of compound **17i**

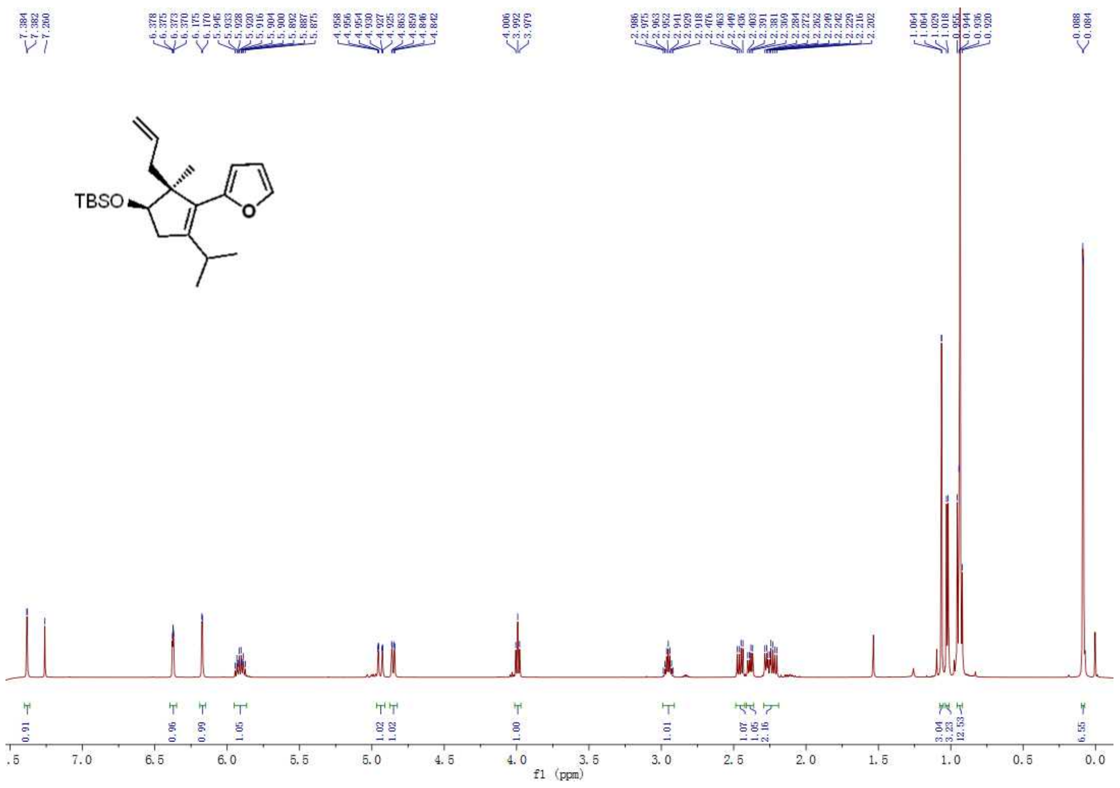

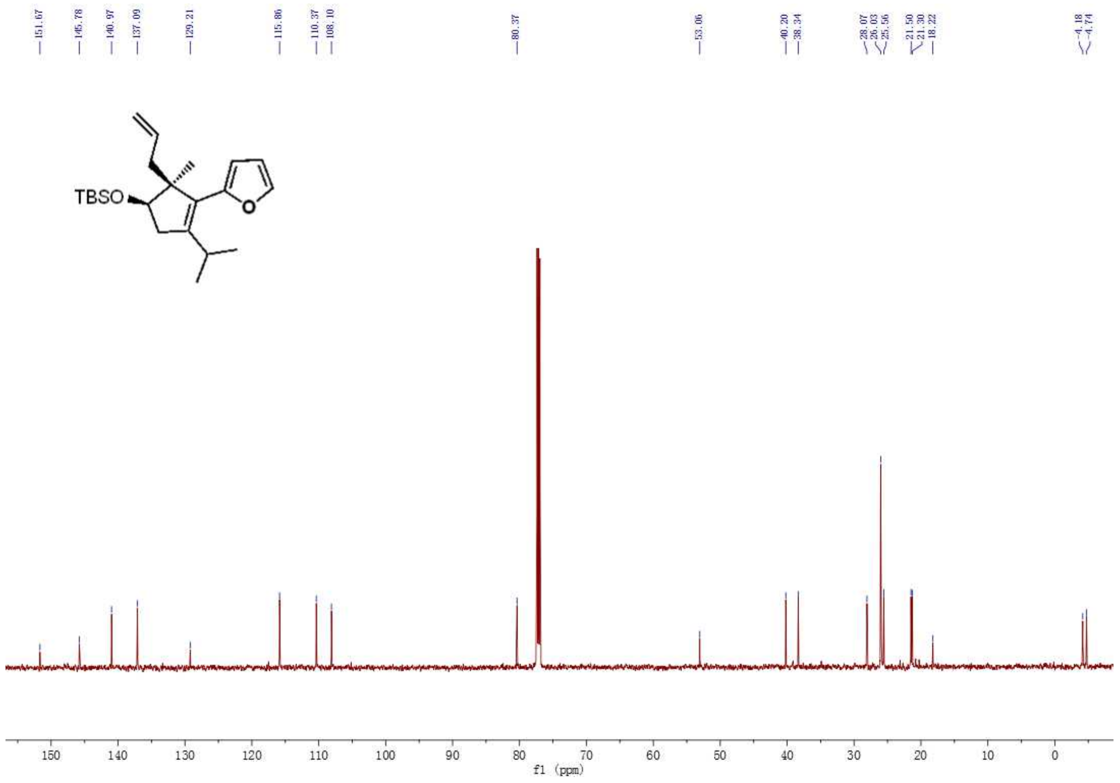

Supplementary Figure 49. ^{1}H and ^{13}C NMR of compound **17j**

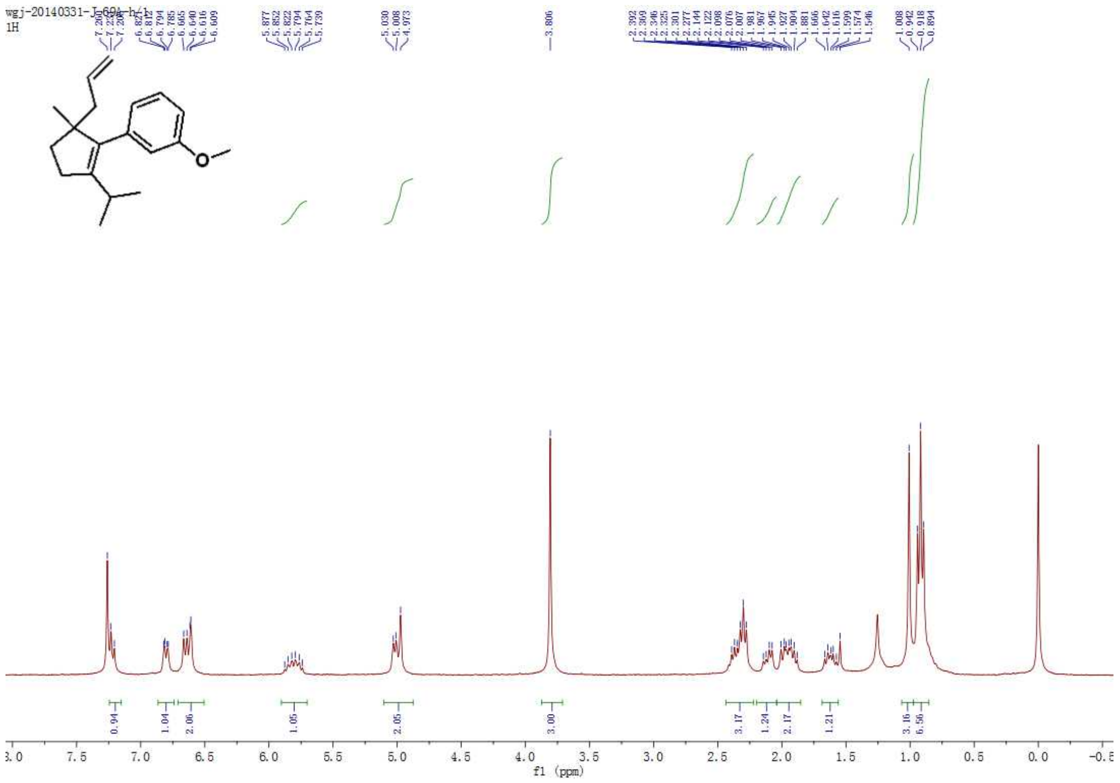

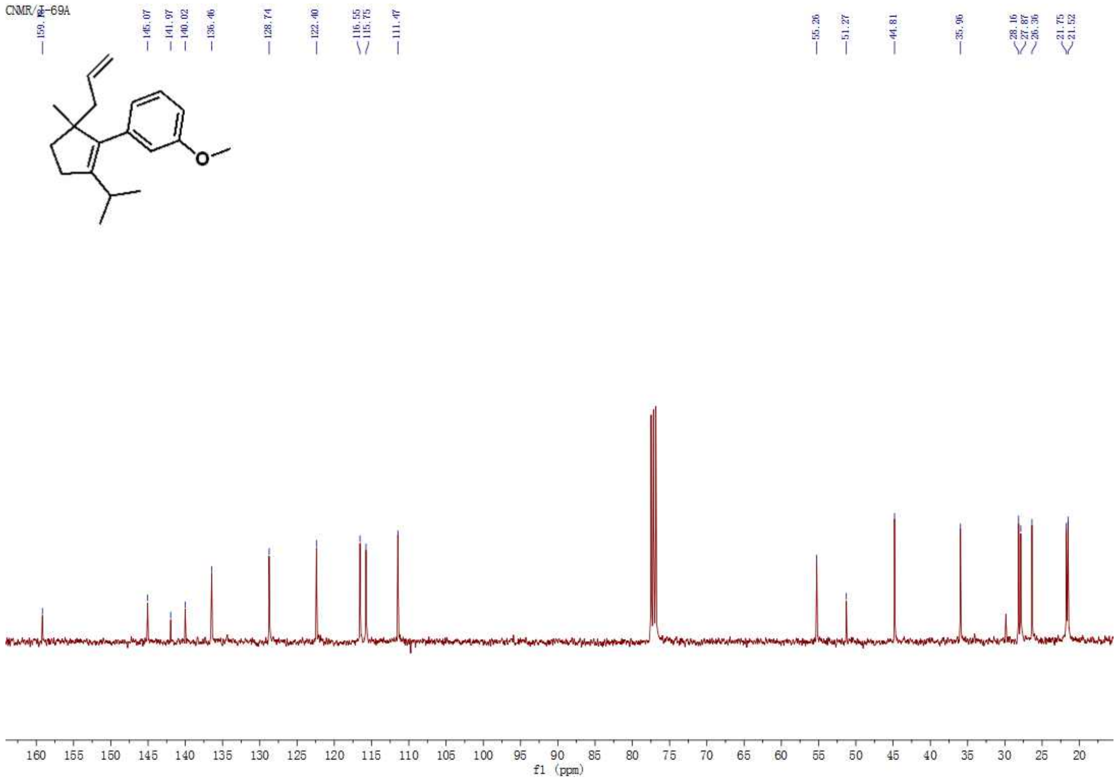

Supplementary Figure 50. ^{1}H and ^{13}C NMR of compound **17k**

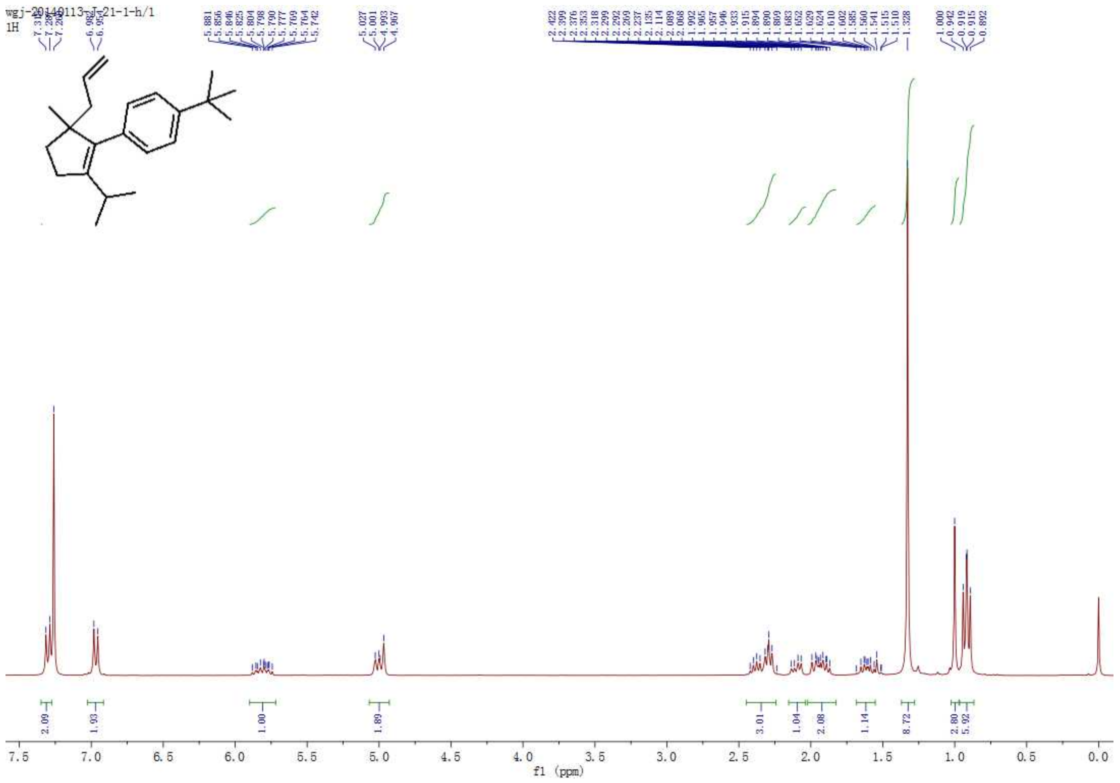

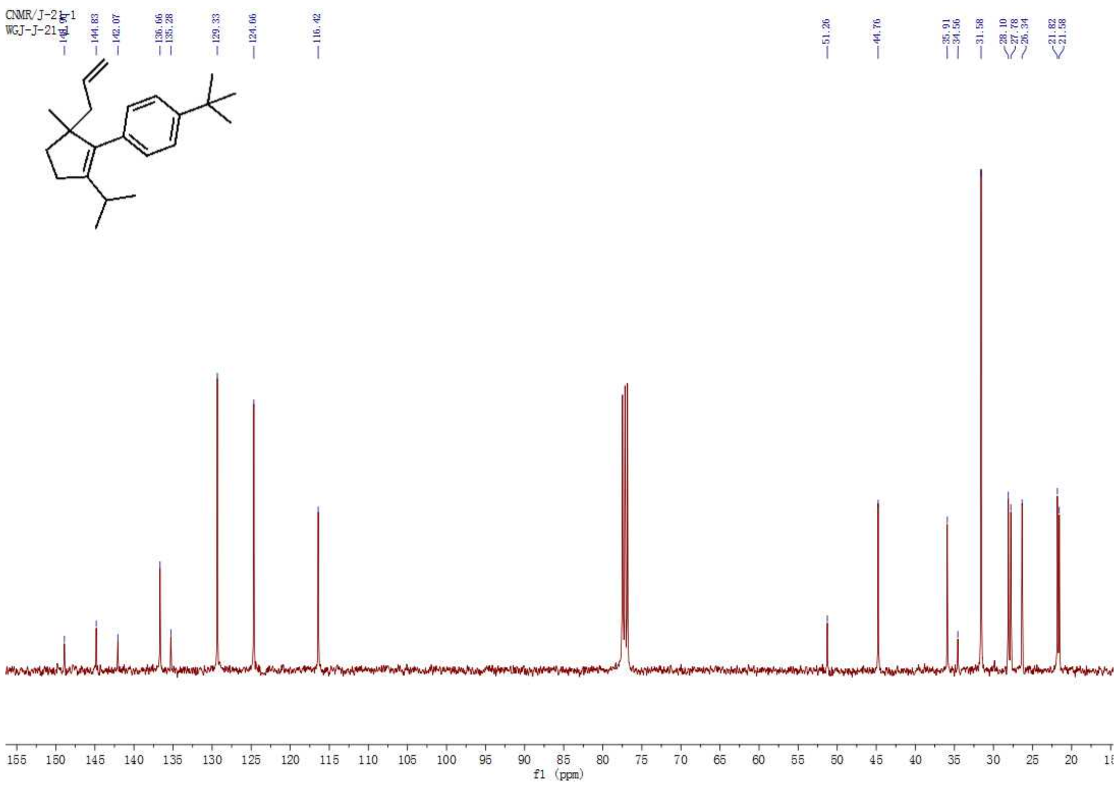

Supplementary Figure 51. ^{1}H and ^{13}C NMR of compound **17l**

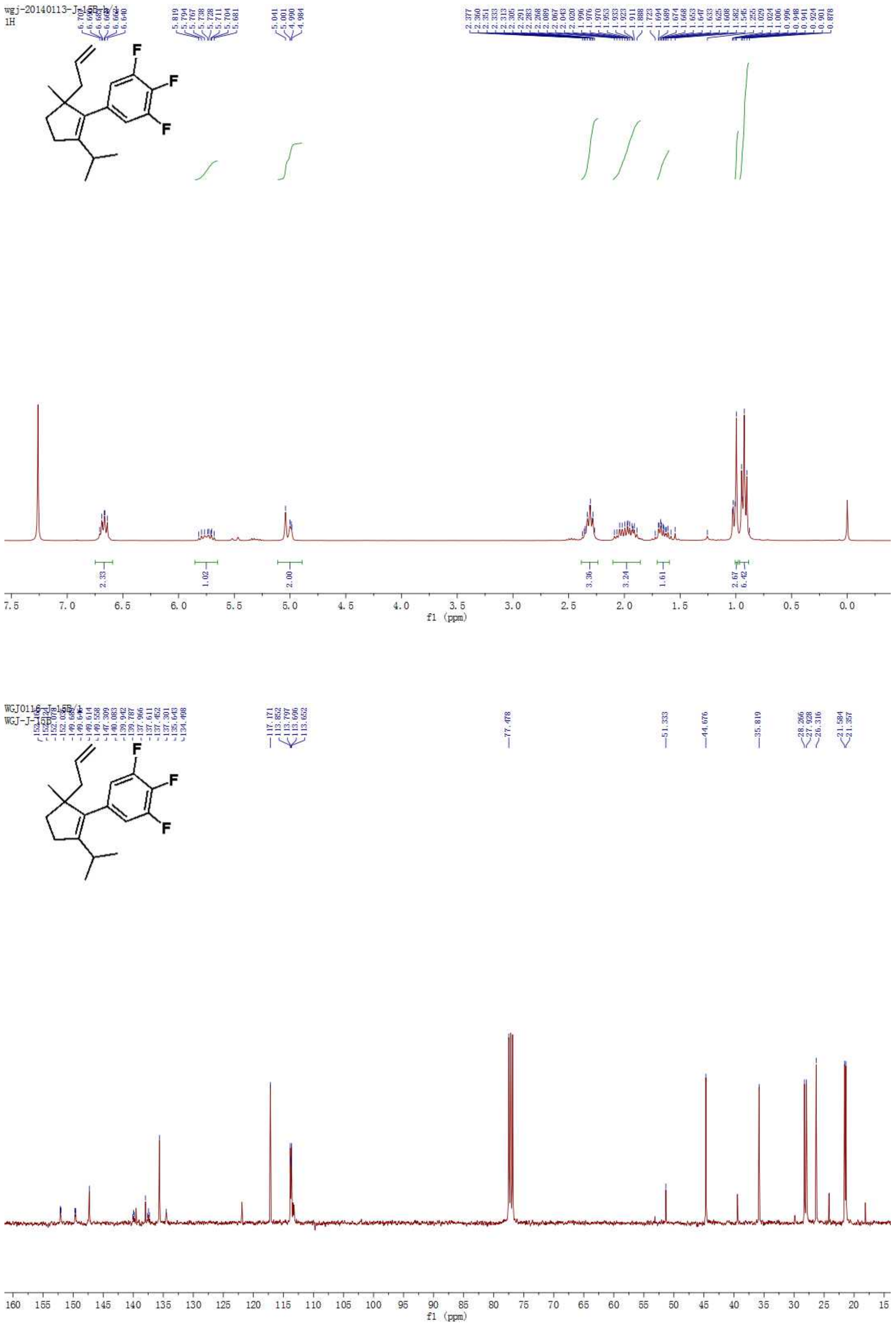

Supplementary Figure 52. ^{1}H and ^{13}C NMR of compound **17m**

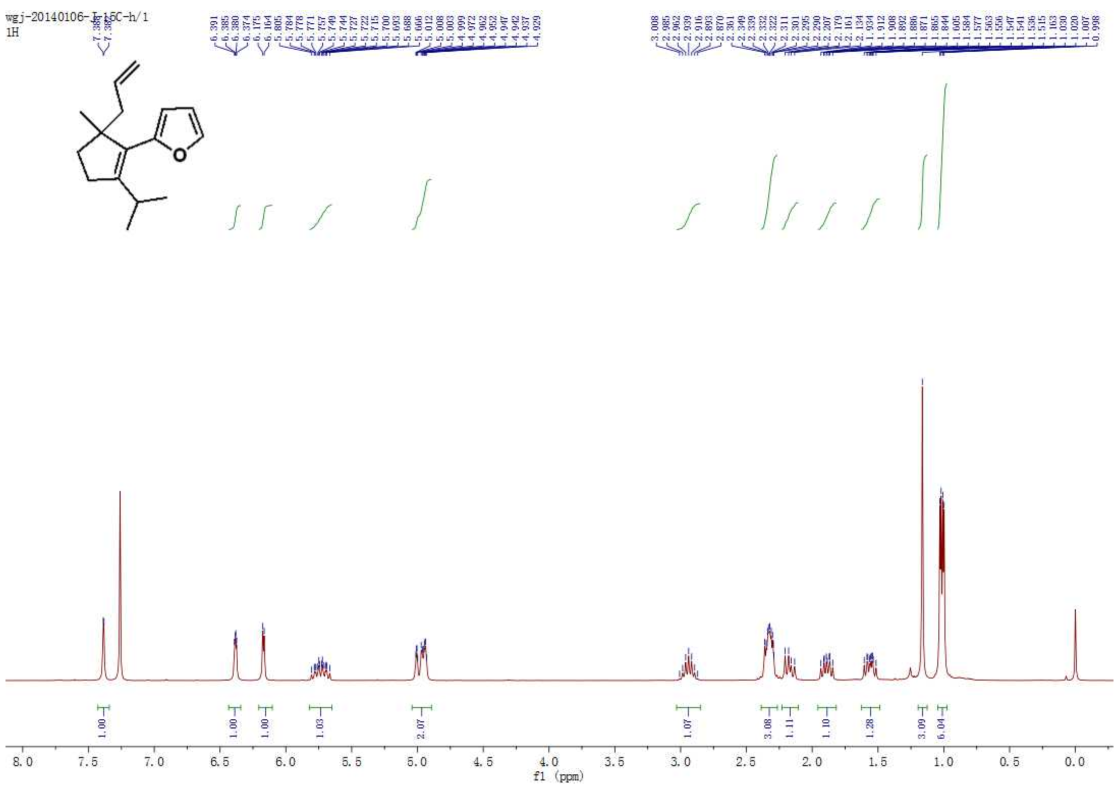

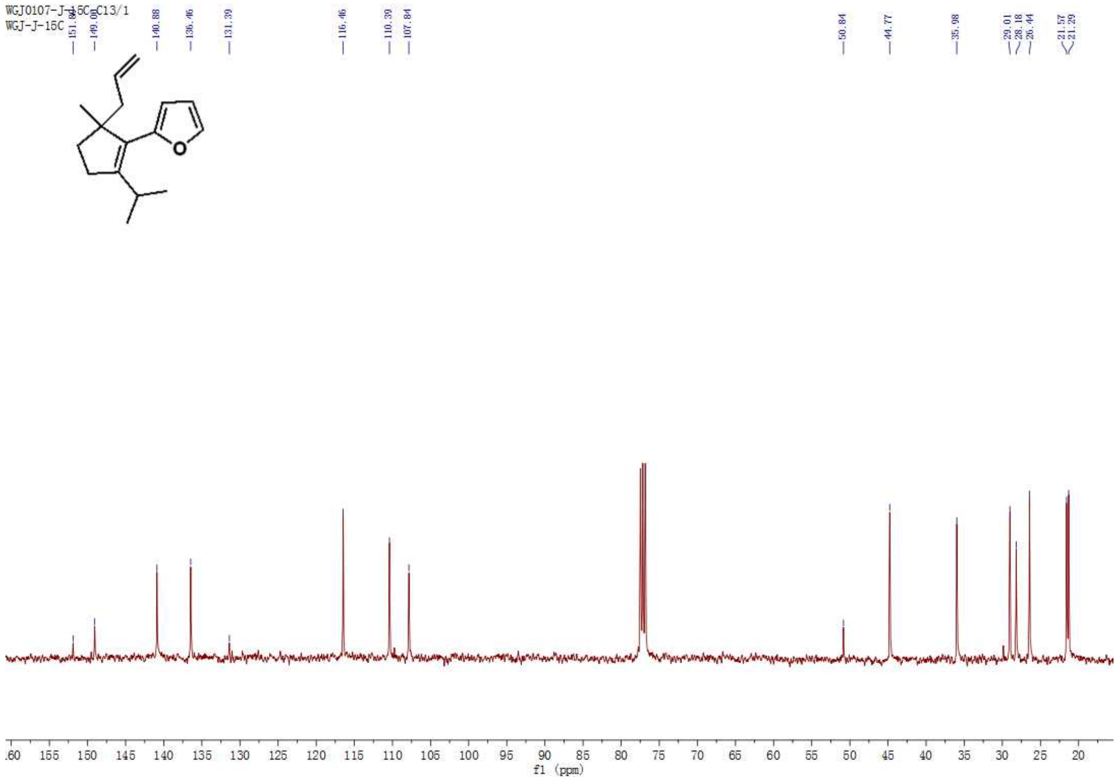

Supplementary Figure 53. ^{1}H and ^{13}C NMR of compound **17n**

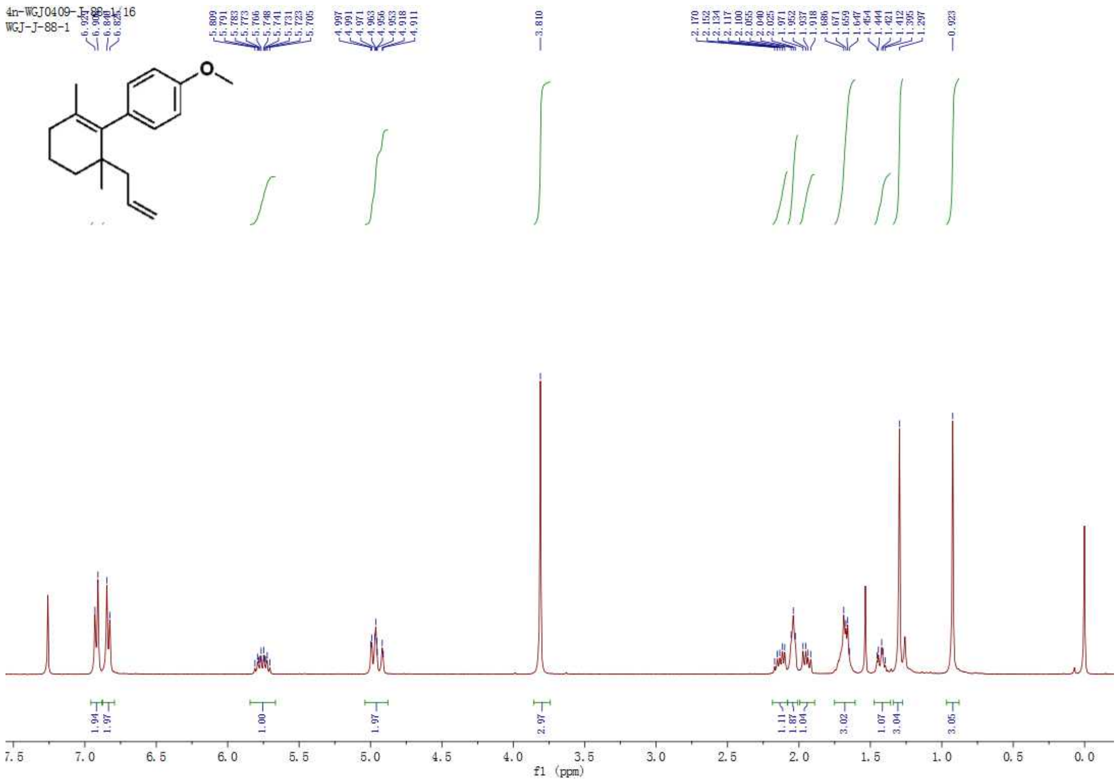

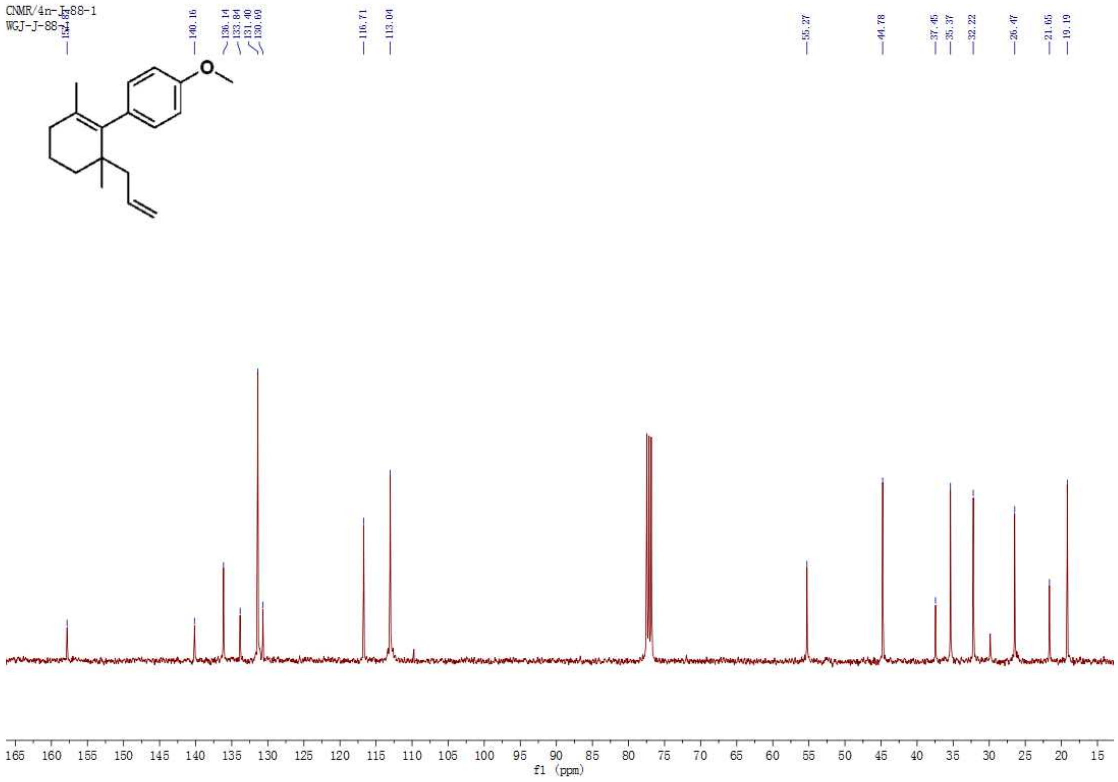

Supplementary Figure 54. ^{1}H and ^{13}C NMR of compound **17o**

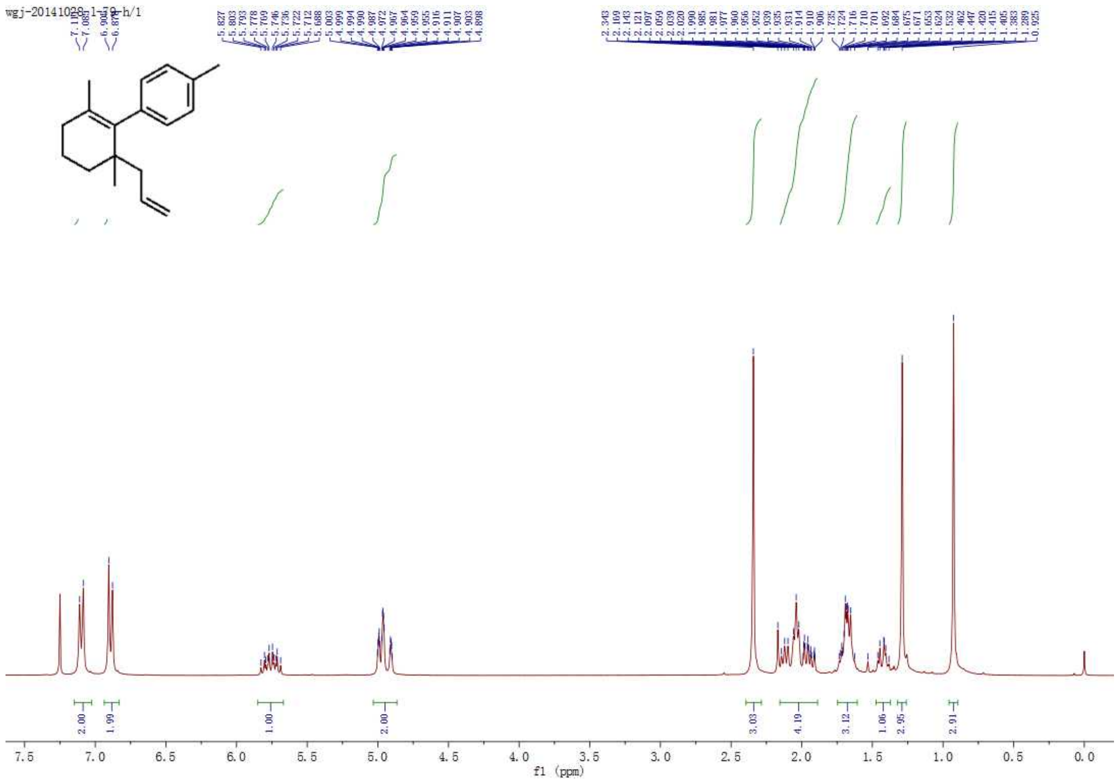

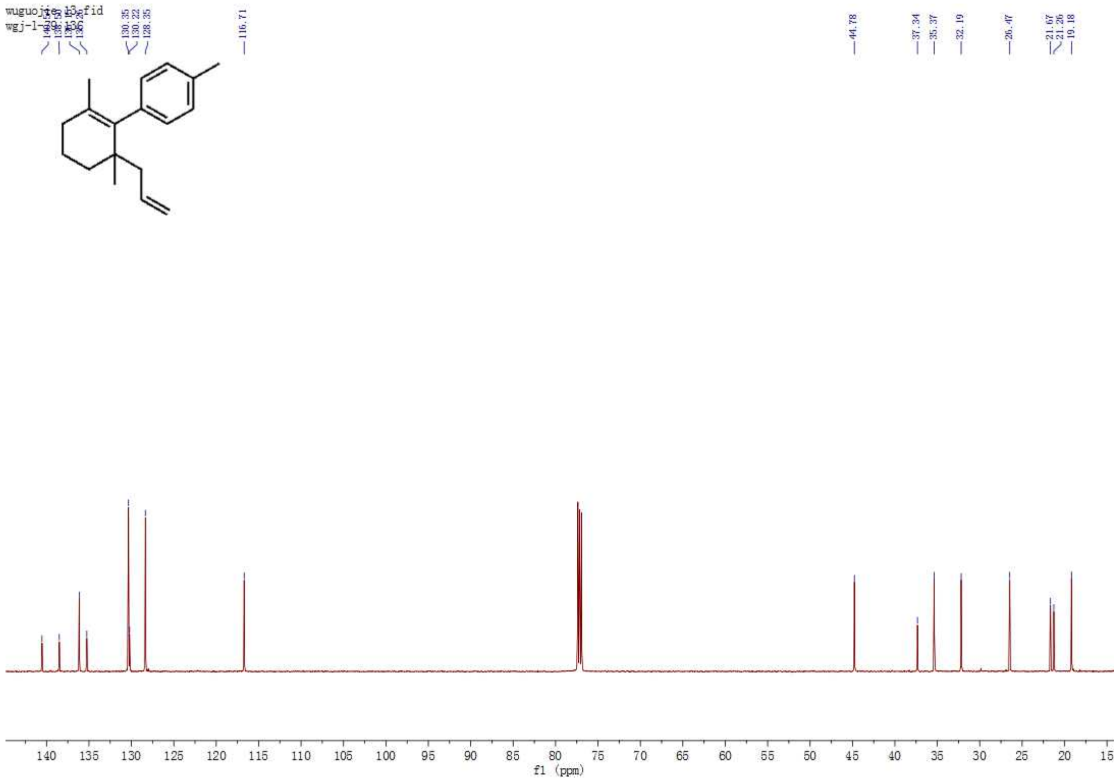

Supplementary Figure 55. ^{1}H and ^{13}C NMR of compound **17p**

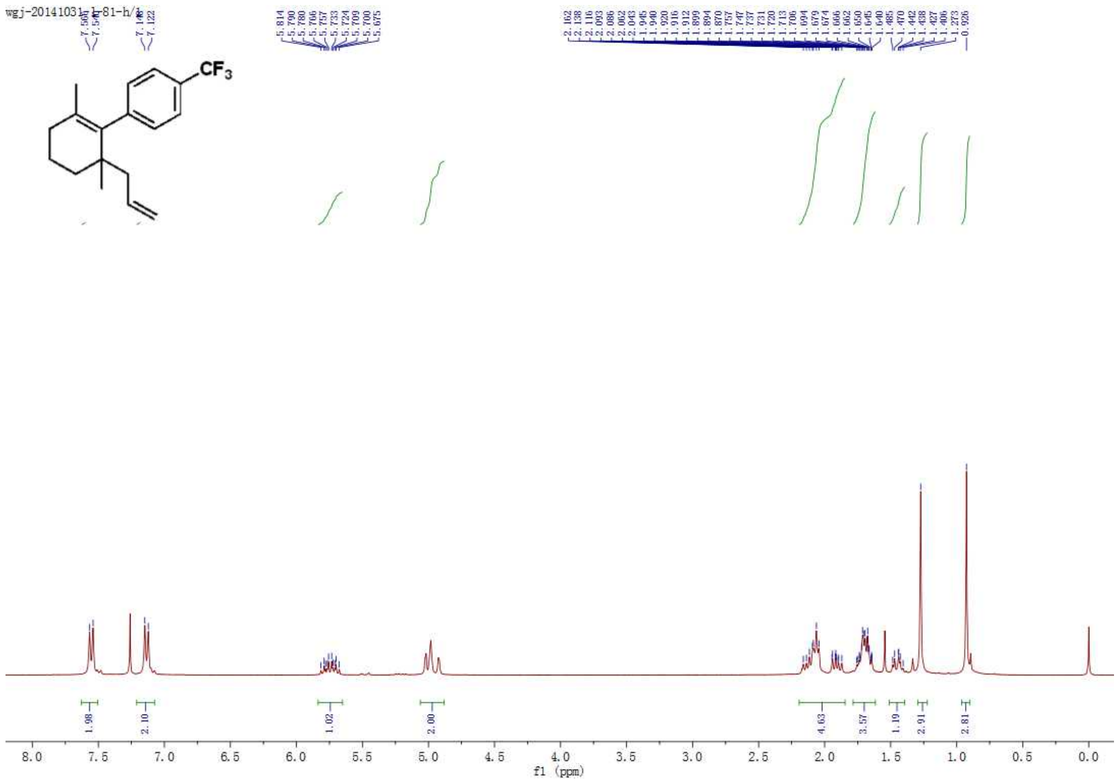

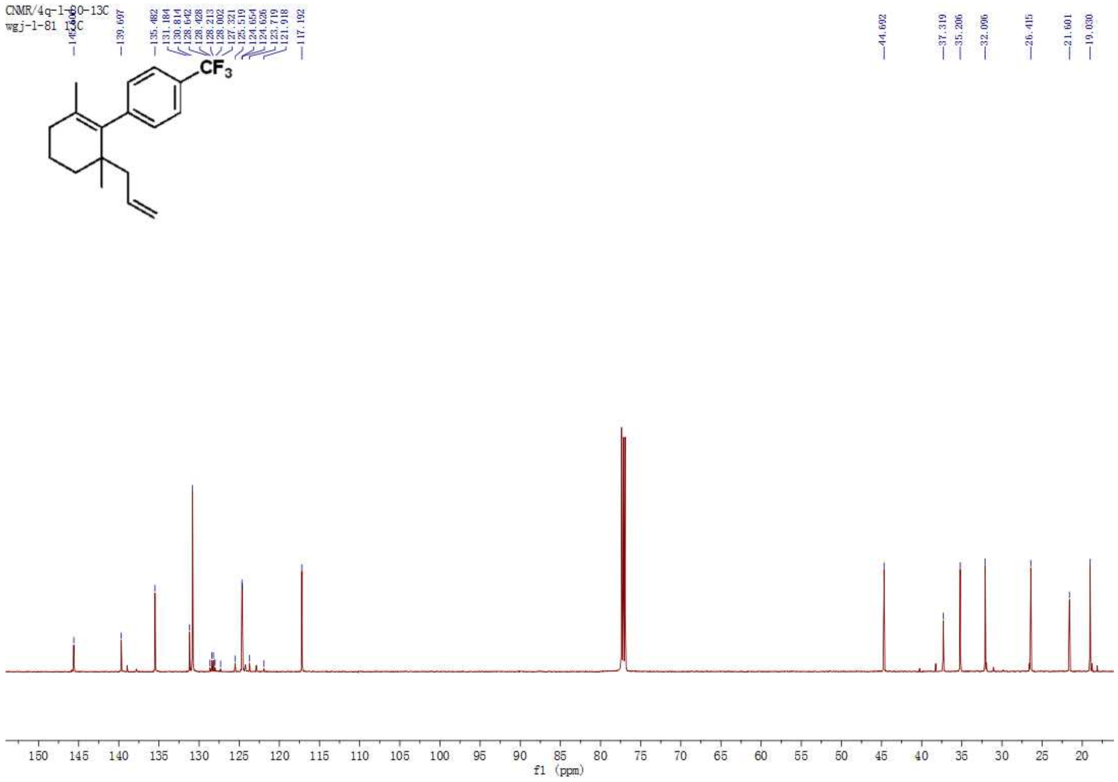

Supplementary Figure 56. ^{1}H and ^{13}C NMR of compound **17q**

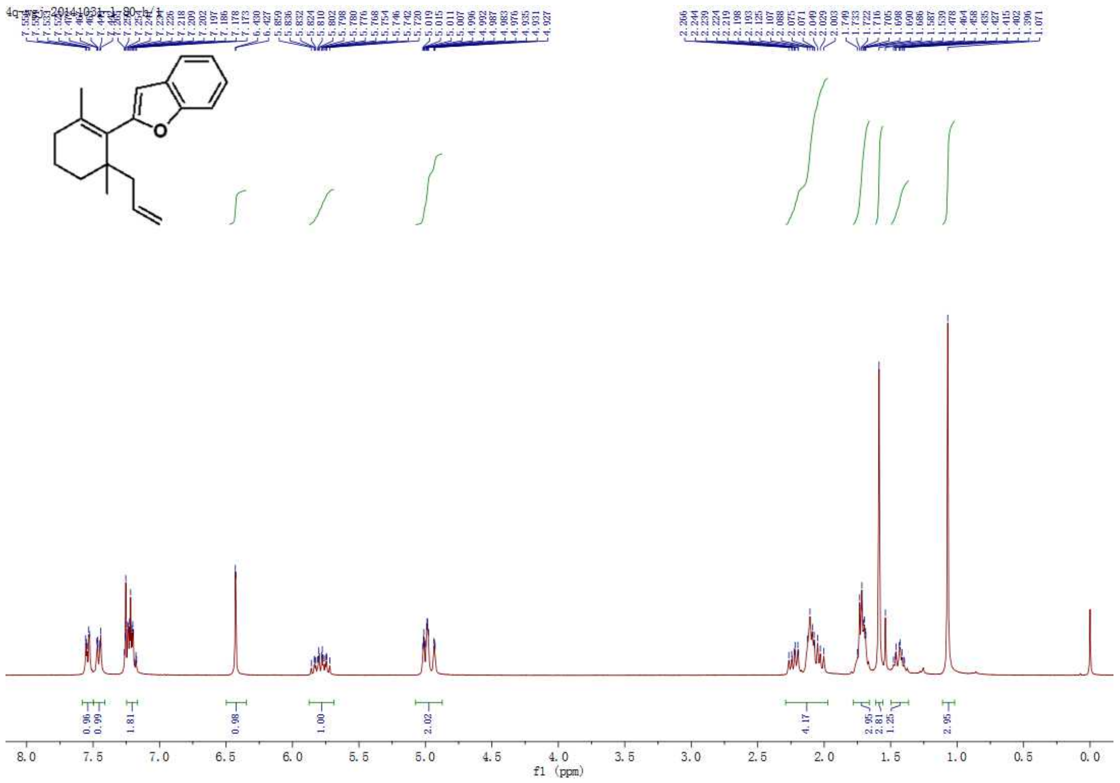

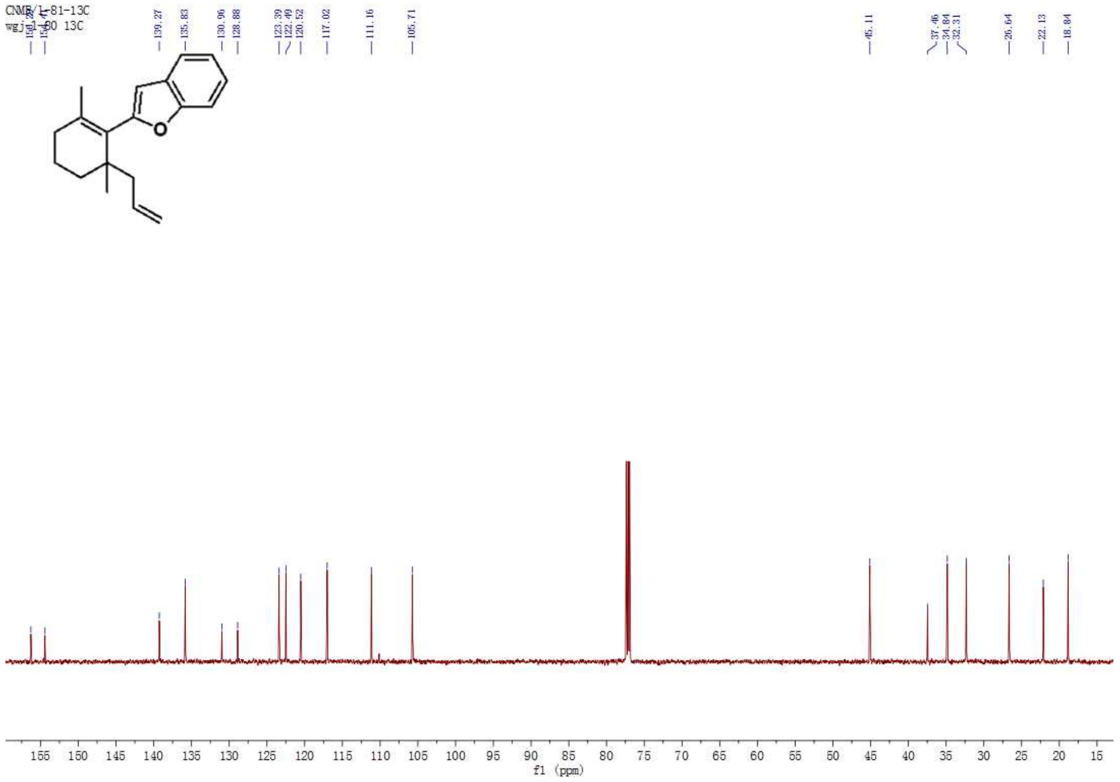

Supplementary Figure 57. ^{1}H and ^{13}C NMR of compound **17r**

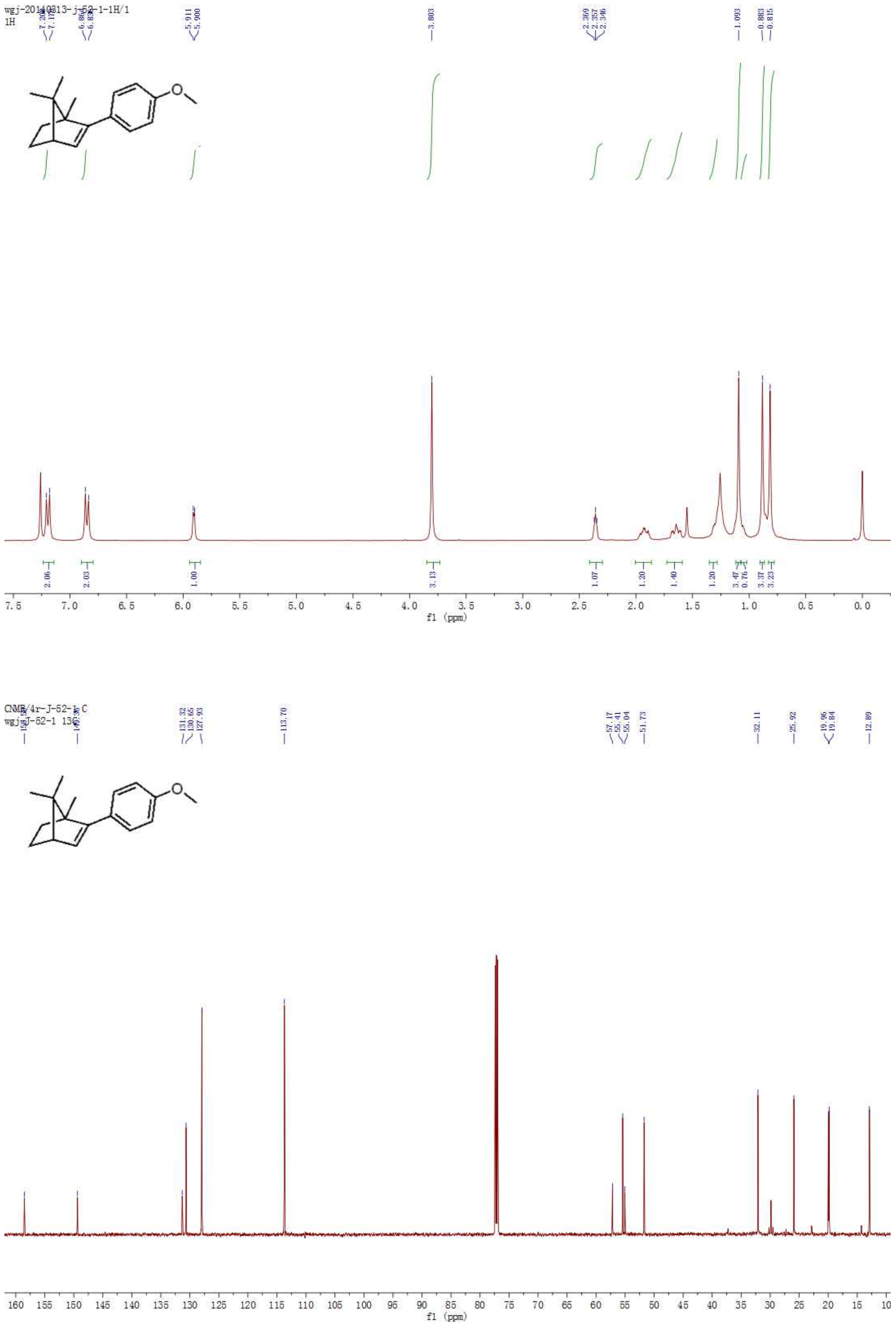

Supplementary Figure 58. ^{1}H and ^{13}C NMR of compound **17s**

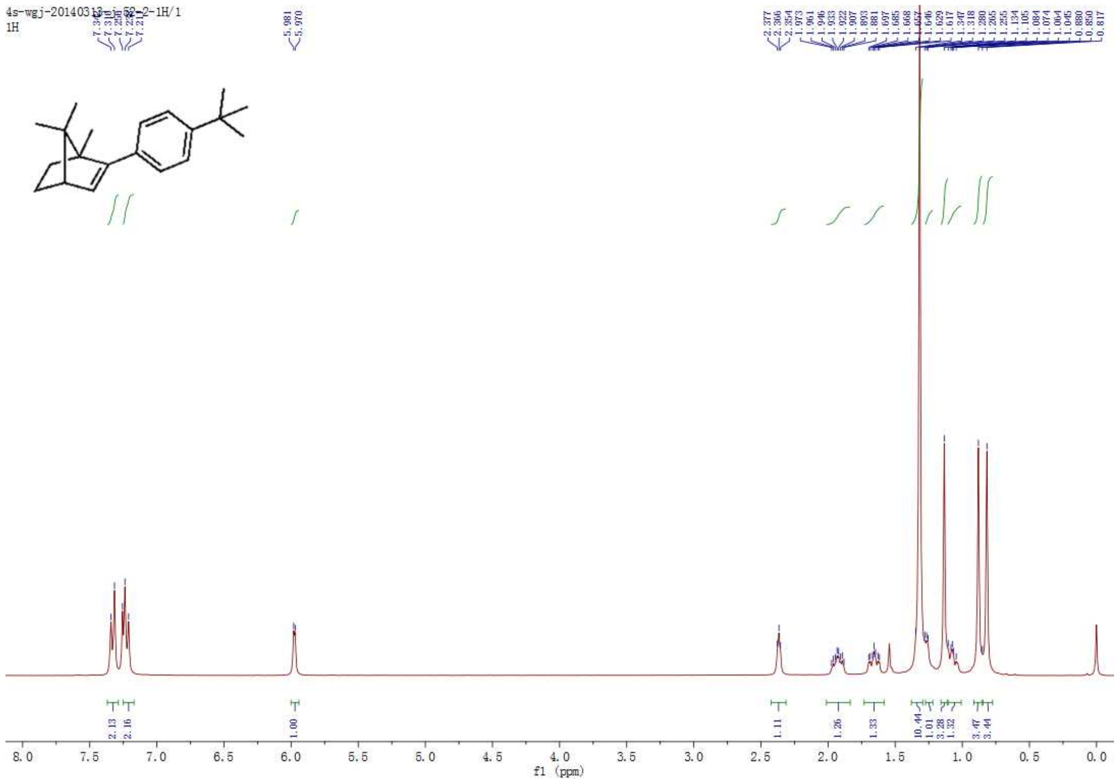

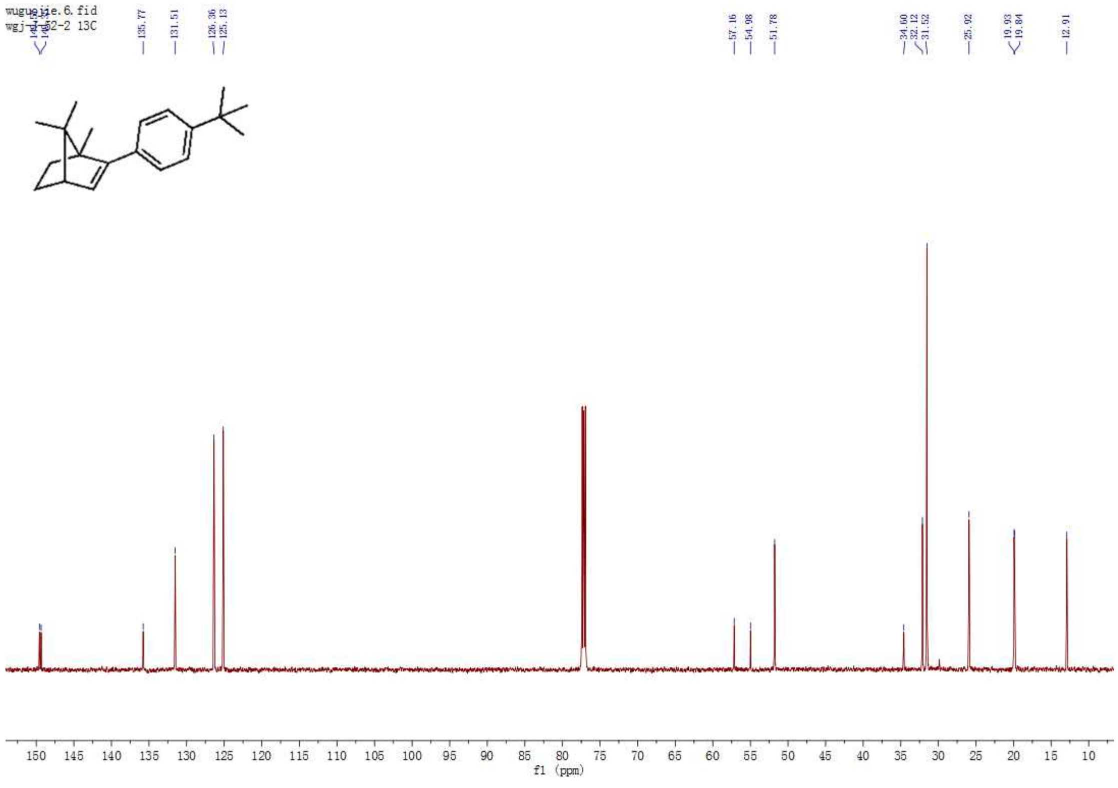

Supplementary Figure 59. ^{1}H and ^{13}C NMR of compound **17t**

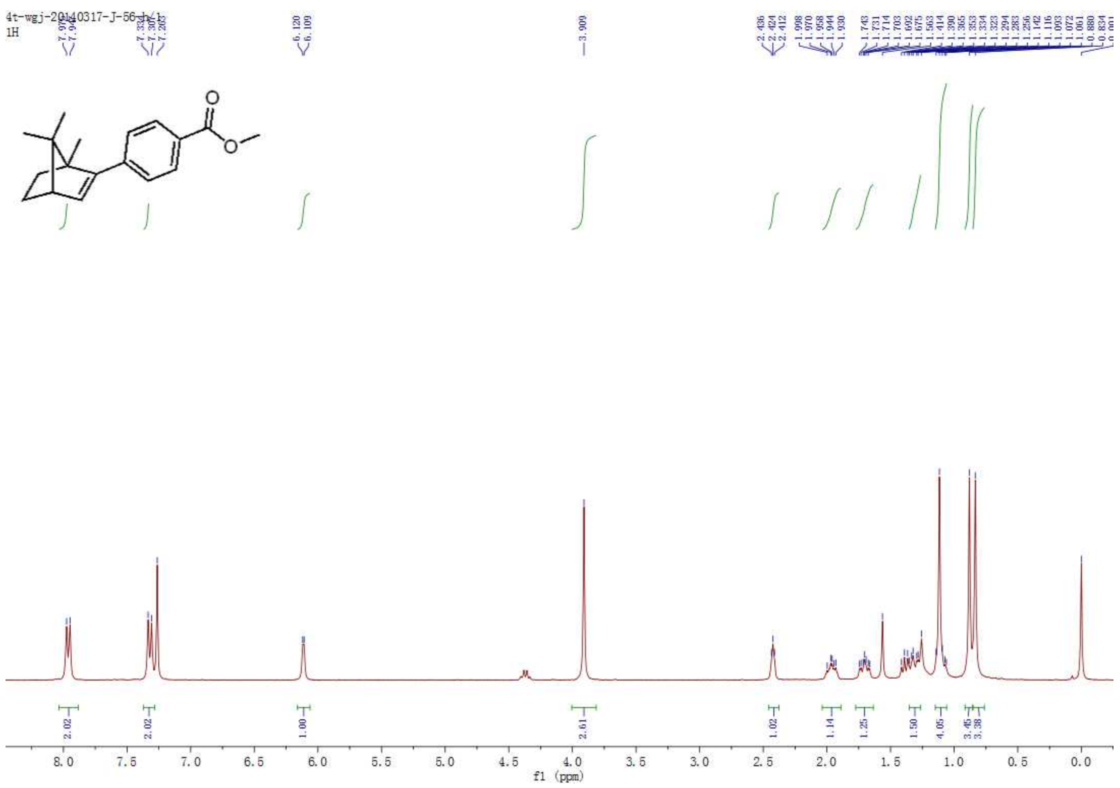

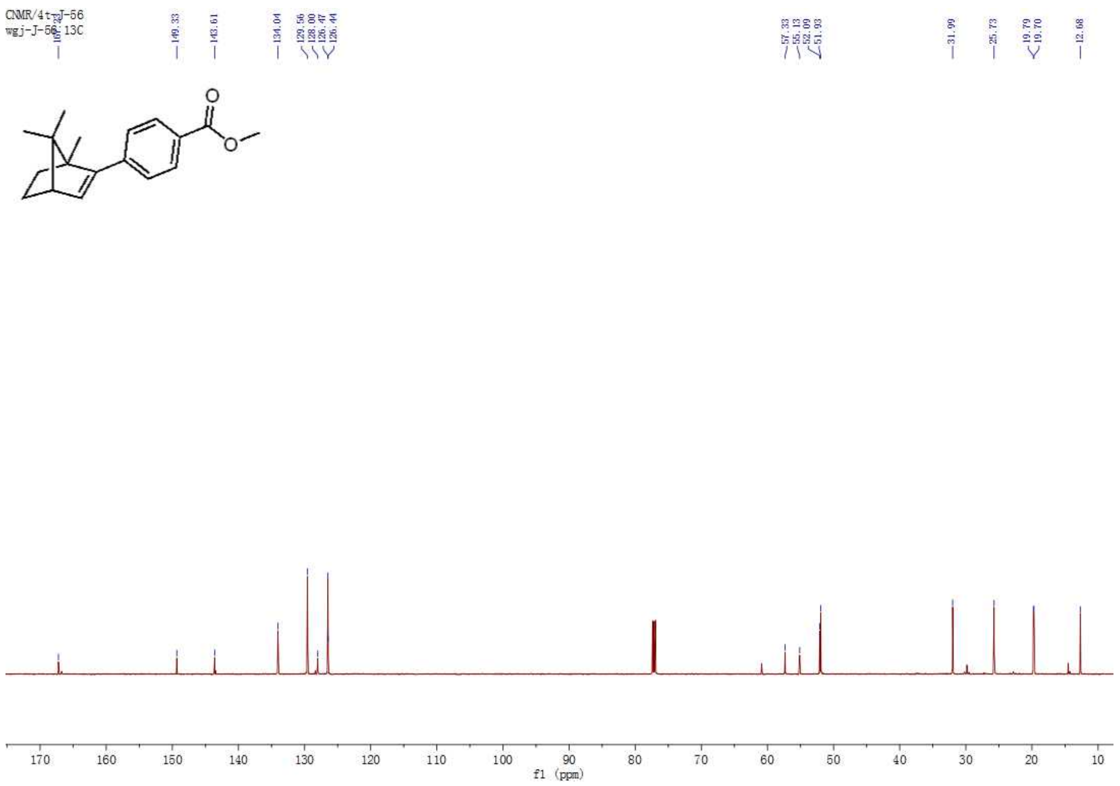

Supplementary Figure 60. ^{1}H and ^{13}C NMR of compound **17u**

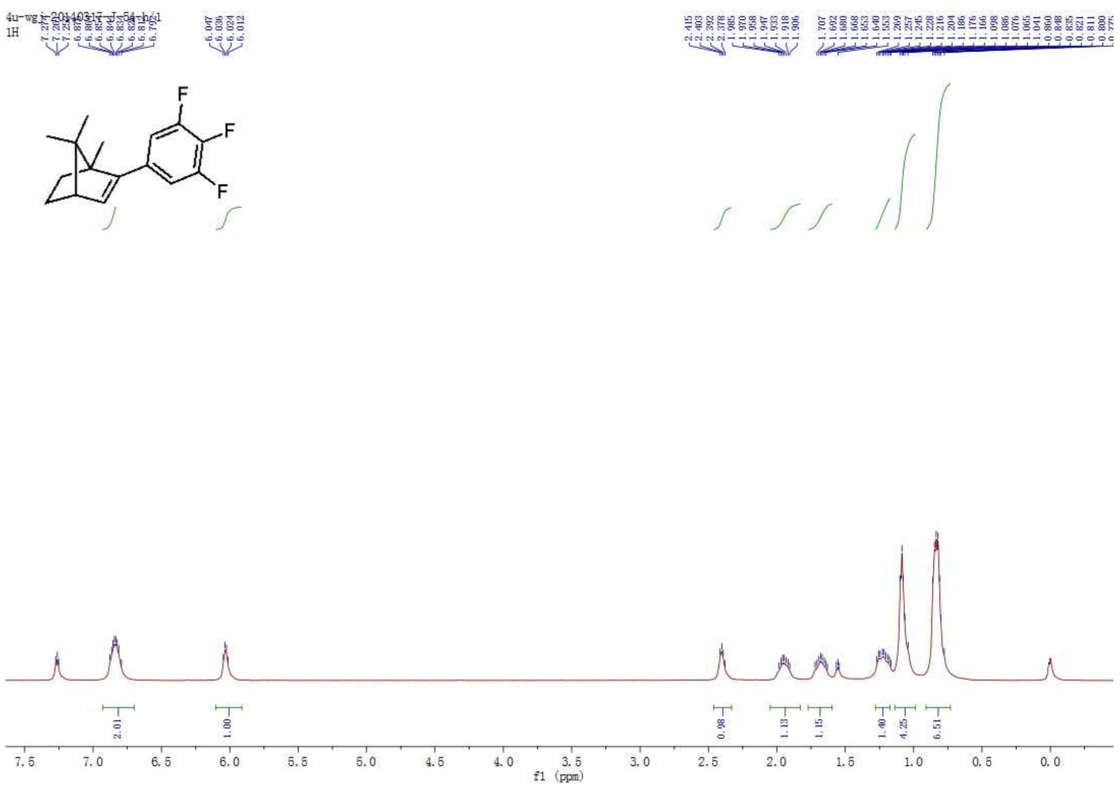

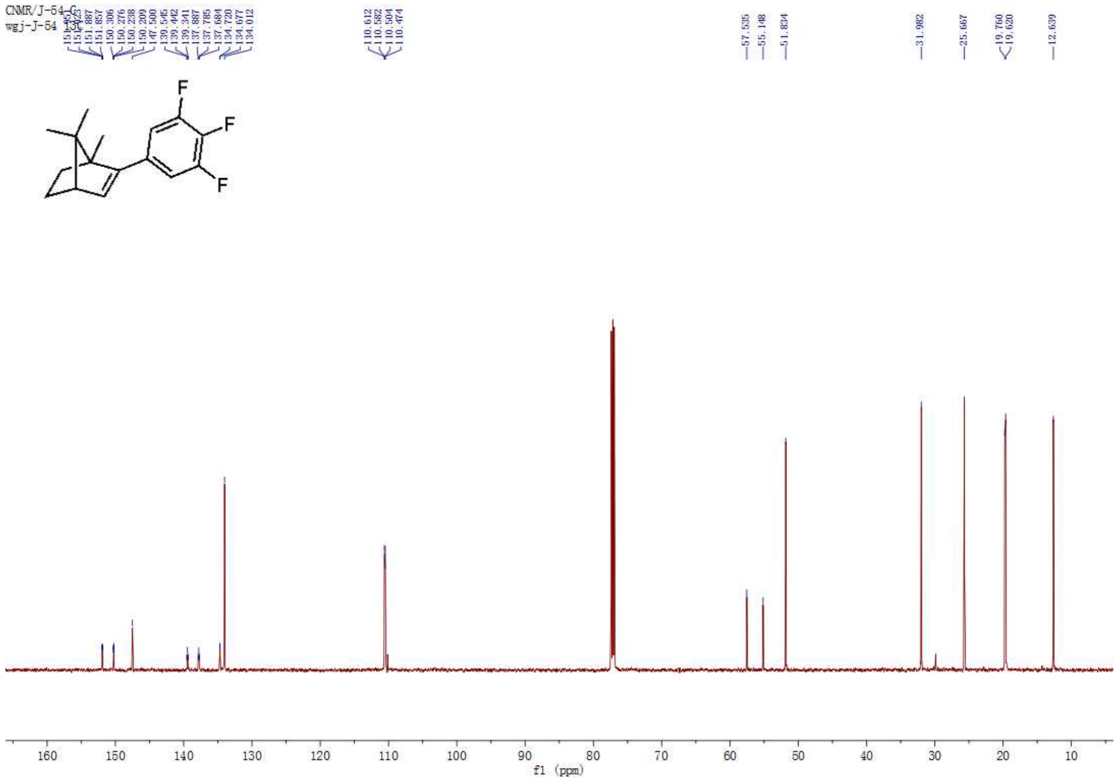

Supplementary Figure 61. ^{1}H and ^{13}C NMR of compound **17v**

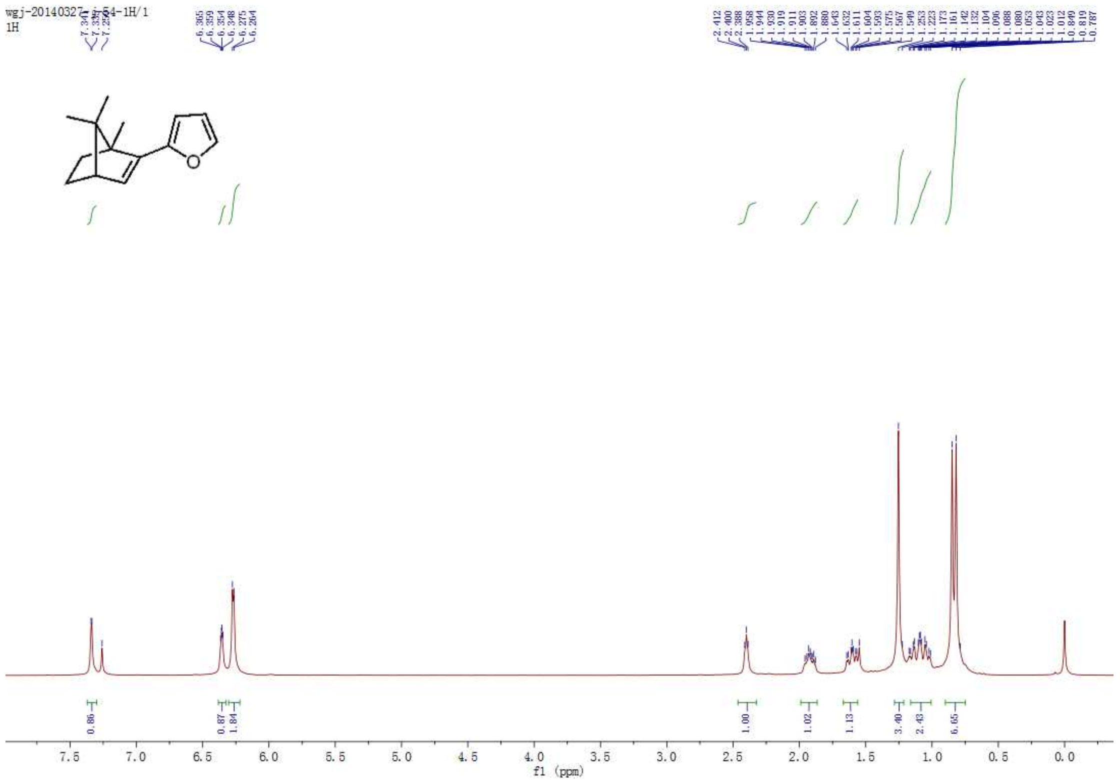

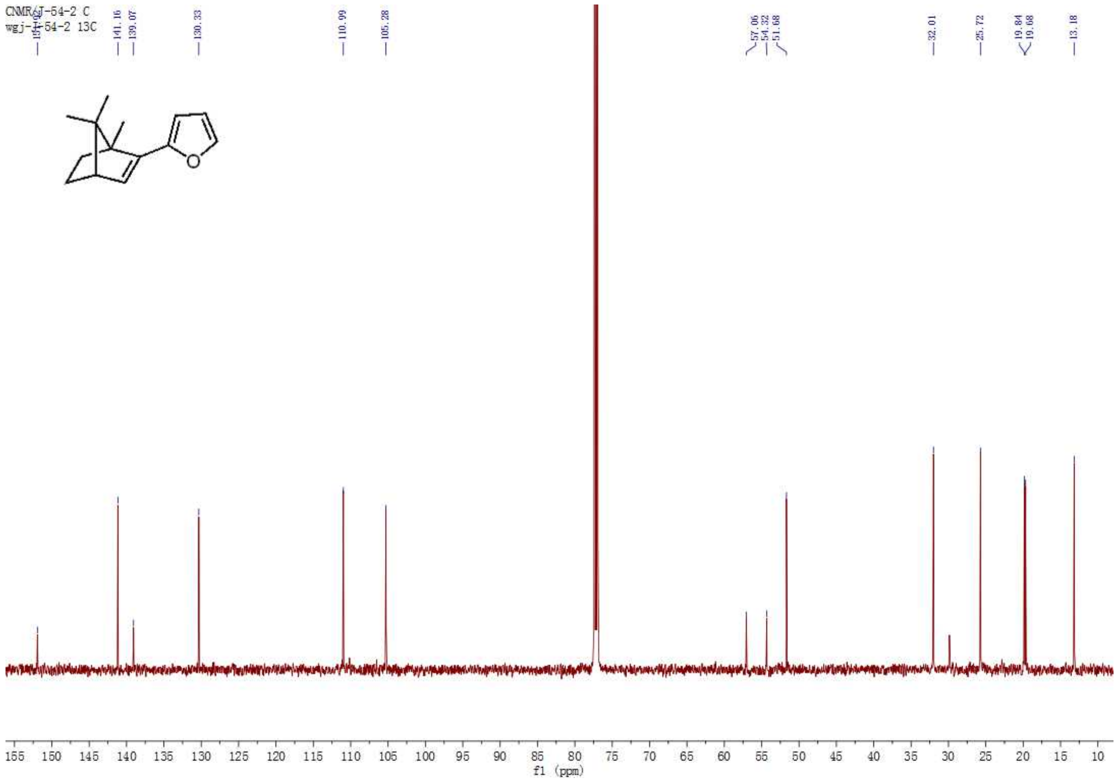

Supplementary Figure 62. ^{1}H and ^{13}C NMR of compound **17w**

9. Supplementary References

1. Marcotullio, M., Pagiotti, R., Maltese, F., Obara, Y., Hoshino, T., Nakahata, N. & Curini, M. *Planta Med.* **72**, 819 (2006).
2. Elamparuthi, E., Fellay, C., Neuburger, M. & Gademann, K. *Angew. Chem. Int. Ed.* **51**, 4071 (2012).
3. Marcotullio, M. C., Pagiotti, R., Campagna, V., Maltese, F., Fardella, G., Altinier, G. & Tubaro, A. *Nat. Prod. Res.* **20**, 917 (2006).
4. Agoeta, W. C. & Smith III, A. B. *J. Org. Chem.* **35**, 3856 (1970).
5. Duan, J. J. W., Lu, Z. & Wrobleski, S. T. *et al*. *Bioorg. Med. Chem. Lett.* **24**, 5721 (2014).
6. Liu, L. L. & Chiu, P. *Chem. Commun.* **47**, 3416 (2011).
7. Tsuji, J., Nisar, M., Shimizu, I. & Minami, I. *Synthesis* **12**, 1009 (1984).
8. Tsuji, J., Yamada, T., Minami, I., Yuhara, M., Nisar, M. & Shimizu, I. *J. Org. Chem.* **52**, 2988 (1987).
9. Craig II, R. A., Loskot, S. A., Mohr, J. T., Behenna, D. C., Harned, A. M. & Stoltz, B. M. *Org. Lett.* **17**, 5160 (2015).
10. Trost, B. M., Pissot-Soldermann, C., Chen, I. & Schroeder, G. M. *J. Am. Chem. Soc.* **126**, 4480 (2004).
11. Nishizawa, M., Iwamoto, Y., Takao, H., Imagawa, H. & Sugihara, T. *Org. Lett.* **2**, 1685 (2000).
12. Tessier, P. E., Nguyen, N., Clay, M. D. & Fallis, A. G. *Org. Lett.* **7**, 767 (2005).
